# Definition of clinically relevant intraoperative hypotension: A data-driven approach

**DOI:** 10.1371/journal.pone.0312966

**Published:** 2024-11-01

**Authors:** Mathias Maleczek, Daniel Laxar, Angelika Geroldinger, Andreas Gleiss, Paul Lichtenegger, Oliver Kimberger

**Affiliations:** 1 Clinical Division of General Anaesthesia and Intensive Care Medicine, Department of Anaesthesia, Intensive Care Medicine and Pain Medicine, Medical University of Vienna, Vienna, Austria; 2 Ludwig Boltzmann Institute for Digital Health and Patient Safety, Medical University of Vienna, Vienna, Austria; 3 Center for Medical Data Science, Institute of Clinical Biometrics, Medical University of Vienna, Vienna, Austria; Hospital de Clinicas de Porto Alegre, BRAZIL

## Abstract

**Background:**

Associations between intraoperative hypotension (IOH) and various postoperative outcomes were shown in retrospective trials using a variety of different definitions of IOH. This complicates the comparability of these trials and makes clinical application difficult. Information about the best performing definitions of IOH regarding 30-day mortality, hospital length of stay (hLOS), and postanesthesia care unit length of stay (PACU-LOS) is missing.

**Methods:**

A retrospective cohort trial was conducted using data from patients undergoing noncardiothoracic surgery. We split the obtained dataset into two subsets. First, we used one subset to choose the best fitting definitions of IOH for the outcomes 30-day mortality, hLOS, and PACU-LOS. The other subset was used to independently assess the performance of the chosen definitions of IOH.

**Results:**

The final cohort consisted of 65,454 patients. In the shaping subset, nearly all tested definitions of IOH showed associations with the three outcomes, where the risk of adverse outcomes often increased continuously with decreasing MAP. The best fitting definitions were *relative time with a MAP (mean arterial pressure) of <80 mmHg* for 30-day mortality, *lowest MAP for one minute* for hLOS, and *lowest MAP for one cumulative minute* for PACU-LOS. Testing these three definitions of IOH in the independent second subset confirmed the associations of IOH with 30-day mortality, hLOS, and PACU-LOS.

**Conclusions:**

Using a data-driven approach, we identified the best fitting definitions of IOH for 30-day mortality, hLOS, and PACU-LOS. Our results demonstrate the need for careful selection of IOH definitions.

**Clinical trial number:** n/a, EC #2245/2020

## Background

Intraoperative hypotension (IOH) is a common complication of anesthesia and serves as an indicator of numerous adverse events [1, 2]. Among these are myocardial injury, stroke, acute kidney injury and delirium [3–11]. Regarding mortality, conflicting evidence is found with a majority showing an association between IOH and mortality [4, 12–15]. The same applies to length of stay (LOS), where an association with IOH was shown in combination with a low mean alveolar concentration of anesthetic agents and low bispectral index (“triple low”) [16]. In contrast, a more recent study on patients undergoing surgery after hip fracture found no association between IOH and LOS [14, 17].

A possible cause of this heterogeneity is usage of many different definitions of IOH in all of the studies: Metastudies showed that over 140 different definitions of IOH were used while the best fitting definitions remain unknown [18–20]. The usage of different definitions of IOH evidently changes the incidence of IOH and therefore possible alters effects [18].

Therefore, *a prior* definition of the best fitting and thereby most associated IOH definition might be a more appropriate approach then using an eminence base approach. The main objective of this trial was to find the best fitting definitions of IOH and their associations with 30-day mortality, hospital LOS (hLOS), and postanesthesia care unit (PACU) LOS in a general surgical population at a large academic center. This can provide future research projects with the certainty which definition to use in order to find relevant results.

## Methods

### Study design

We conducted a retrospective cohort study using the Medical University of Vienna’s perioperative database which was previously used by the authors in a similar statistical approach to show an association between IOH and PONV [21]. All adult (≥ 18 years) patients undergoing non-emergency, non-cardiothoracic surgery between 1.8.2014 and 1.10.2020 with a complete record of intraoperative blood pressure and complete documentation regarding hLOS were included. All patients with missing information were excluded. For this retrospective study that used all available data within a predefined time period we expected at least 50,000 cases meeting the inclusion criteria. This was deemed sufficient for the planned statistical analyses.

### Ethics

The ethics committee (Borschkegasse 8b/6, 1090 Vienna, President: Prof. Zezula) of the Medical University of Vienna approved this study and waived the need for informed consent on 23.12.2020 (EKNr: 2245/2020). All research was performed in accordance with relevant guidelines and regulations, including the Declaration of Helsinki.

### Data sources

The Medical University of Vienna is a large tertiary center with approximately 50.000 surgical cases a year. The routinely used electronic health records system Philips IntelliSpace Critical Care and Anesthesia (Philips, Amsterdam, Netherlands) and the hospital information system (SAP, Walldorf, Germany) were used as data sources. Those databases contain all relevant data including vital signs, procedures, orders and outcomes. Before 2017, blood pressure values were available every two minutes, thereafter every 15 seconds. Information on patients’ deaths were obtained from the hospital information system and combined with data from the Federal Statistical Office about mortality.

The data were accessed on 20.4.2021; no data identifying the patient (i.e.: names) were accessed.

### Data processing

As anesthetic records contain artifacts possibly altering results, artifact cleaning was performed using the following six rules as used before [6, 21–23]: 1) Systolic pressure greater than or equal to 300 mmHg, 2) Systolic pressure lower than or equal to 20 mmHg, 3) Diastolic pressure lower than or equal to 5 mmHg, 4) Diastolic pressure greater than or equal to 225 mmHg, 5) Systolic pressure lower than or equal to diastolic pressure + 5 mmHg, and 6) Values lying outside of three standard deviations from a patient’s mean blood pressure.

After artifact filtering, blood pressure values were interpolated linearly to 15 second intervals, where necessary, to aid application of the definitions on a homogeneous sample. Intraoperative hypotension was defined using the following definitions based on the mean arterial pressure (MAP): 1) Absolute measures of IOH: a) Lowest MAP for 1, 3, 5 and 10 sustained minutes [6], b) Lowest MAP for 1, 3, 5 and 10 cumulative minutes per patient [6], c) Absolute time with an MAP under 50, 55, 60, 65, 70, 75 or 80 mmHg [6], and 2) Relative time with an MAP under 50, 55, 60, 65, 70, 75 or 80 mmHg compared to the duration of anesthesia. The particular MAP value for a certain duration of a hypotensive episode (1, 3, 5, or 10 min) was defined as the upper “low MAP” limit of the lowest MAP episode for the respective number of minutes [21].

Thirty-day mortality was defined as death within 30 days after the end of surgery independent of the cause of death. Data about time of death were combined from the hospital information system and the Federal Statistical Office. Hospital length of stay was calculated as the difference between date of discharge and date of admission of the case during which surgery took place. To ensure consistent data and equal weight for each patient in the analysis, we only included the first surgery of a patient in our analyses. PACU-LOS was defined as the difference between the date of discharge and the date of admission to the PACU. Patients admitted to the ICU immediately after surgery were excluded from this particular analysis. The LOS and PACU-LOS datasets were subset of the mortality dataset.

To correct for patients’ comorbidities, the weighted Charlson comorbidity index [24] was calculated using R [25] (R Foundation for Statistical Computing, Vienna, Austria, comorbidity package 0.5.3).

### Statistical analysis

The statistical framework of this study was described similarly before in [21]: The aims of the study were structured into two sequential steps for each of the three outcomes (30-day mortality, hLOS and PACU LOS): 1) to determine which definition of hypotension has the strongest association with 30-day mortality, hLOS, and PACU-LOS and 2) to estimate the association between the best fitting hypotension definition identified in Aim 1 and the outcomes: 30-day mortality, hLOS and PACU-LOS.

To prevent overfitting, the data had to be spit in two independent datasets of equal size by randomly allocating each patient to either one of the datasets after applying the exclusion criteria mentioned above: The first half was used as “shaping dataset” to identify the best fitting of the 22 definitions of IOH for each of the three outcomes. This was done using a univariable, a multivariable logistic regression model (for binary outcome 30-day mortality) and two linear regression models (for the metric outcomes hLOS and PACU-LOS).

The second half was used as an “estimation dataset” to show the associations of the best fitting definitions of IOH in an independent dataset.

For the 583 patients (0.9%) who died before discharge, during their hospital stay, LOS was set to infinity, and the 1%-trimmed hLOS was used as the outcome in all further analyses. Thus, all results for LOS refer to the 99% ‘best’ patients (i.e., patients with a shorter hLOS). The 1% trimmed hLOS and PACU-LOS were log-transformed for use as dependent variables in the models in order to stabilize the residual distribution; the results were back-transformed to the original scale for graphical presentation. Each of the multivariable models included one of the definitions of IOH as well as the following adjustment variables: sex, age, BMI, duration of surgery, time from hospital admission to surgery, surgical specialty, ASA classification and comorbidity score. Those covariables were chosen as they are commonly seen as predictors of mortality and length of stay. Definitions of IOH, duration of surgery, time from hospital admission to surgery, and comorbidity score were represented by natural cubic spline bases using three degrees of freedom; age and BMI were represented in the same way using two degrees of freedom. For the definitions of IOH “Absolute time with a MAP under 50, 55, 60, 65, 70, 75 or 80 mmHg” and “relative time with a MAP under 50, 55, 60, 65, 70, 75 or 80 mmHg”, an additional, binary variable indicating whether the value of the definition of hypotension was equal to zero was included. This was necessary to account for spikes at zero in the distributions of these IOH definitions.

Definitions of hypotension, BMI, and duration of surgery were winsorized at the 99^th^ percentile.

For the definitions of IOH a spline fit was used in each model in order to capture more nuanced effects than just linear ones. Therefore, a definition’s effect on an outcome is best represented graphically. Effect sizes were deduced from the respective spline fit for clinically relevant points: MAP values of 50, 65 and 75mmHg, durations of 5 & 10 minutes for absolute values, and 5 & 10% for relative values.

For each of the multivariable models, the performance was assessed by calculating the Brier score (for the binary mortality outcome) and the Mean Squared Error (MSE) (for metric outcomes) using 10-fold cross-validation with 40 repetitions. The “best-fitting” definition of IOH was determined by either the smallest cross-validated Brier score or the smallest cross-validated MSE, as appropriate.

In order to investigate the uncertainty of this selection of an optimal IOH definition we report a 0.95 confidence set on models for each outcome, defined as the set of models (i.e. IOH definitions) where in the list of increasing Akaike weights the cumulative weights exceed 0.95 [26]. For the second step, we estimated the association between hypotension and each outcome by fitting a logistic or linear regression model, as appropriate, on the “estimation dataset” with the best-fitting definition of IOH identified in step 1 and the adjustment variables (parameterized as in step 1) as independent variables. The independence of the shaping and estimation datasets ensured that the estimation of the association between the best-fitting definition of IOH and the respective outcome was not affected by a multiple comparison bias. Finally, we compared the contribution of the best-fitting definition of IOH against the contribution of the adjustment variables included in the multivariable model by calculating the increase in cross-validated Brier scores or in cross-validated MSE values, as appropriate, on the estimation dataset, when excluding one of the variables from the model. Descriptive statistics are presented as the median and interquartile range (IQR) or as the mean and standard deviation (SD) as appropriate.

A sensitivity analysis was conducted to evaluate the results’ robustness by using 1.5% and 2% as trimming cut-offs for hLOS and using 4 degrees of freedom for the spline fit of IOH definitions.

All data processing and statistical calculations were carried out using Python 3.9 (with pandas 1.3.4 and NumPy 1.19.2 packages; Centrum voor Wiskunde en Informatica Amsterdam, Netherlands) and R 4.1.2 (R Foundation for Statistical Computing, Vienna, Austria) [27–29].

## Results

**Fig 1:**
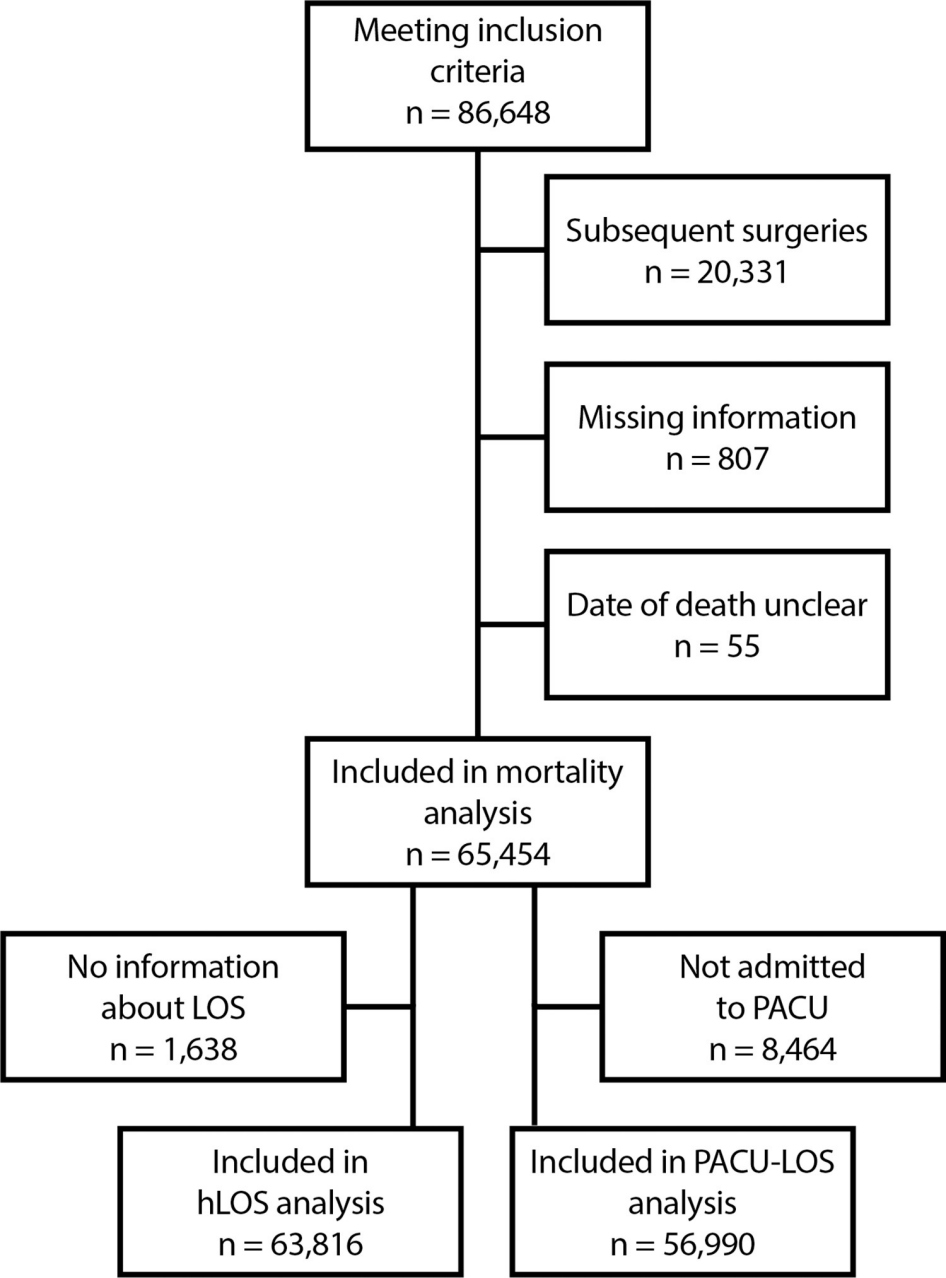
Inclusion of patients. The figure shows the flowchart of patient inclusion. Abbreviations: hLOS: hospital length of stay; PACU: postanesthesia care unit; PACU-LOS: postanesthesia care unit length of stay; n, number. Please see S2.4. Table in S2 File for details of missing information.

Of all available cases, 86,648 surgeries met the predefined inclusion criteria. After excluding subsequent surgical cases per patient, cases with missing information about patient demographics (i.e., sex, ASA classification or body mass index (BMI)), and cases with uncertain dates of death, we finally included 65,454 patients in the analysis on mortality (general dataset) and 63,816 patients in the analysis on hLOS (Fig 1). By further excluding all patients not admitted to a PACU, we finally included 56,990 patients in the analysis of PACU LOS (Fig 1).

The median age of the cohort was 53 (IQR: 37–68) years, and 37,039 (56.6%) patients were female. Of all included patients, 580 (0.9%) died within 30 days after surgery. The median hospital LOS was 5.1 (IQR: 3.1–10.3) days, and the median PACU-LOS was 2.1 (IQR: 1.6–2.9) h. Randomly splitting the dataset into a shaping dataset and an estimation dataset led to two sets with 32,727 patients each. All demographic details are shown in Table 1.

**Table 1:** Demographic details.

	Mortality	LOS	PACU LOS
**Number**	65454	63816	56990
**Age (Quartile)**	52.8 (36.8, 67.9)	52.9 (36.8, 67.9)	51.5 (35.9, 67.0)
**BMI (Quartile)**	25.7 (22.8, 29.5)	25.7 (22.8, 29.5)	25.7 (22.8, 29.6)
**Female sex (%)**	37039 (56.6%)	35892 (56.2%)	32941 (57.8%)
**30-day mortality (%)**	580 (0.9%)	569 (0.9%)	282 (0.5%)
**Duration of surgery [min] (Quartile)**	101.2 (61.0, 161.5)	102.0 (61.0, 162.2)	94.0 (58.5, 144.5)
**Comorbidity score (Quartile)**	0.0 (0.0, 1.0)	0.0 (0.0, 1.0)	0.0 (0.0, 1.0)
**LOS Hospital [d] (Quartile)**	5.1 (3.1, 10.3)	5.1 (3.1, 10.4)	4.3 (3.0, 8.6)
**Time to surgery [d] (Quartile)**	1.0 (0.8, 1.3)	1.0 (0.8, 1.2)	1.0 (0.8, 1.2)
**ASA classification n (%)**			
**ASA 1**	19425 (29.7%)	18876 (29.6%)	18246 (32.0%)
**ASA 2**	29777 (45.5%)	28992 (45.4%)	26664 (46.8%)
**ASA 3**	14915 (22.8%)	14631 (22.9%)	11509 (20.2%)
**ASA 4**	1337 (2.0%)	1317 (2.1%)	571 (1.0%)
**Comorbidities n (%)**			
**Myocardial infarction**	324 (0.5%)	324 (0.5%)	219 (0.4%)
**Congestive heart failure**	1182 (1.8%)	1169 (1.8%)	892 (1.6%)
**Peripheral vascular disease**	1543 (2.4%)	1535 (2.4%)	1064 (1.9%)
**Cerebrovascular disease**	1648 (2.5%)	1634 (2.6%)	716 (1.3%)
**Dementia**	239 (0.4%)	238 (0.4%)	202 (0.4%)
**COPD**	2154 (3.3%)	2136 (3.3%)	1684 (3.0%)
**Rheumatic disease**	261 (0.4%)	254 (0.4%)	223 (0.4%)
**Peptic ulcer disease**	130 (0.2%)	128 (0.2%)	85 (0.1%)
**Liver disease**	1285 (2.0%)	1273 (2.0%)	938 (1.6%)
**Diabetes**	3149 (4.8%)	3127 (4.9%)	2538 (4.5%)
**Diabetes with complications**	448 (0.7%)	445 (0.7%)	374 (0.7%)
**Hemiplegia**	102 (0.2%)	100 (0.2%)	67 (0.1%)
**Renal disease**	2017 (3.1%)	2004 (3.1%)	1612 (2.8%)
**Solid tumor**	11237 (17.2%)	11046 (17.3%)	8772 (15.4%)
**Leukemia**	197 (0.3%)	195 (0.3%)	106 (0.2%)
**Metastatic tumor**	1541 (2.4%)	1533 (2.4%)	936 (1.6%)
**AIDS**	99 (0.2%)	98 (0.2%)	92 (0.2%)
**Surgical area, n (%)**			
**Urology, gynecology, general surgery**	24024 (36.7%)	23236 (36.4%)	21274 (37.3%)
**Maxillofacial, ENT, derma**	9395 (14.4%)	9261 (14.5%)	8717 (15.3%)
**Orthopedics, trauma**	17255 (26.4%)	16816 (26.4%)	15857 (27.8%)
**Neurosurgery**	3590 (5.5%)	3571 (5.6%)	1241 (2.2%)
**Non-OR anesthesia, obstetrics**	9935 (15.2%)	9705 (15.2%)	8767 (15.4%)
**Robotic surgery**	1255 (1.9%)	1227 (1.9%)	1134 (2.0%)

LOS: length of stay, PACU: postoperative care unit, BMI: body mass index, ASA: American association of anesthesiologist score, min: minutes, d: days, COPD: chronic obstructive pulmonary disease, AIDS: Acquired Immunodeficiency Syndrome, n: number, ENT: ear-nose-throat, non-or: non operating room

In the general dataset, the median duration with a MAP < 65 mmHg was 4.3 (IQR: 0.0–20.3) minutes, and the median lowest MAP sustained for 5 minutes was 67 (IQR: 62–75) mmHg. Additional details can be found in Table 2.

**Table 2:** Definitions in the general dataset, alive and dead after 30 days, mean of variables is shown.

	Overall	30 day survivor	30 day nonsurvivor
**Number**	65454	64874	580
**Age (Quartile)**	52.8 (36.8, 67.9)	52.6 (36.7, 67.7)	73.6 (63.5, 81.6)
**BMI (Quartile)**	25.7 (22.8, 29.5)	25.7 (22.8, 29.6)	24.2 (21.4, 27.7)
**Duration of surgery [min] (Quartile)**	101.2 (61.0, 161.5)	101.0 (61.0, 161.0)	134.0 (75.7, 222.3)
**Comorbidity score (Quartile)**	0.0 (0.0, 1.0)	0.0 (0.0, 1.0)	2.0 (0.0, 3.0)
**LOS Hospital [d] (Quartile)**	5.1 (3.1, 10.3)	5.1 (3.1, 10.2)	15.0 (7.4, 24.9)
**Time to surgery [d] (Quartile)**	1.0 (0.8, 1.3)	1.0 (0.8, 1.2)	2.0 (0.8, 8.4)
**Female SEX**	37039 (56.6%)	36772 (56.7%)	267 (46.0%)
**ASA classification n (%)**			
**ASA 1**	19425 (29.7%)	19420 (29.9%)	5 (0.9%)
**ASA 2**	29777 (45.5%)	29709 (45.8%)	68 (11.7%)
**ASA 3**	14915 (22.8%)	14568 (22.5%)	347 (59.8%)
**ASA 4**	1337 (2.0%)	1177 (1.8%)	160 (27.6%)
**IOH definitions [mmHg] median (Quartile)**			
**Lowest MAP sustained for**			
**1 minute**	62.2 (56.4, 70.5)	62.2 (56.5, 70.5)	58.5 (52.0, 67.0)
**3 minutes**	64.9 (59.5, 72.6)	65.0 (59.5, 72.6)	61.8 (56.0, 70.0)
**5 minutes**	67.0 (61.7, 74.6)	67.0 (61.8, 74.6)	64.7 (59.4, 73.0)
**10 minutes**	71.0 (65.3, 79.0)	71.0 (65.4, 79.0)	69.0 (63.0, 78.0)
**15 minutes**	74.0 (68.0, 82.6)	74.0 (68.0, 82.6)	72.0 (66.0, 81.8)
**Lowest MAP sustained for**			
**1 cumulative minute**	62.0 (56.0, 70.3)	62.0 (56.0, 70.4)	57.5 (51.1, 67.0)
**3 cumulative minutes**	64.0 (58.2, 72.0)	64.0 (58.2, 72.0)	60.0 (54.0, 68.9)
**5 cumulative minutes**	65.1 (59.8, 73.3)	65.2 (59.8, 73.3)	61.5 (56.0, 70.5)
**10 cumulative minutes**	67.6 (62.0, 76.0)	67.6 (62.0, 76.0)	64.0 (58.2, 73.0)
**15 cumulative minutes**	69.3 (63.3, 78.2)	69.4 (63.4, 78.2)	66.0 (60.0, 75.0)
**Absolute time [min] with a MAP**			
**under 50 mmHg**	0.0 (0.0, 0.0)	0.0 (0.0, 0.0)	0.0 (0.0, 0.2)
**under 55 mmHg**	0.0 (0.0, 0.0)	0.0 (0.0, 0.0)	0.0 (0.0, 3.1)
**under 60 mmHg**	0.0 (0.0, 5.0)	0.0 (0.0, 5.0)	2.4 (0.0, 13.8)
**under 65 mmHg**	4.2 (0.0, 20.2)	4.2 (0.0, 20.2)	11.4 (0.0, 35.1)
**under 70 mmHg**	16.2 (0.0, 45.8)	16.2 (0.0, 45.7)	28.1 (3.7, 74.8)
**under 75 mmHg**	33.0 (7.5, 73.8)	33.0 (7.5, 73.2)	51.2 (12.7, 115.4)
**under 80 mmHg**	49.2 (18.5, 97.5)	49.0 (18.5, 97.0)	76.2 (22.9, 152.7)
**Relative time [min] with a MAP**			
**under 50 mmHg**	0.0 (0.0, 0.0)	0.0 (0.0, 0.0)	0.0 (0.0, 0.0)
**under 55 mmHg**	0.0 (0.0, 0.0)	0.0 (0.0, 0.0)	0.0 (0.0, 0.0)
**under 60 mmHg**	0.0 (0.0, 0.0)	0.0 (0.0, 0.0)	0.0 (0.0, 0.1)
**under 65 mmHg**	0.0 (0.0, 0.2)	0.0 (0.0, 0.2)	0.1 (0.0, 0.2)
**under 70 mmHg**	0.2 (0.0, 0.4)	0.2 (0.0, 0.4)	0.2 (0.0, 0.5)
**under 75 mmHg**	0.4 (0.1, 0.6)	0.4 (0.1, 0.6)	0.4 (0.1, 0.7)
**under 80 mmHg**	0.6 (0.2, 0.8)	0.6 (0.2, 0.8)	0.6 (0.3, 0.8)

LOS: length of stay, PACU: postoperative care unit, BMI: body mass index, ASA: American association of anesthesiologist score, min: minutes, d: days, n: number, MAP: mean arterial pressure, IOH: intraoperative hypotension

The shaping dataset was used to find the best fitting definition (step 1). Most of the definitions were negatively associated with mortality, hLOS, and PACU-LOS in univariable models. Exceptions were definitions using relative time with a MAP below a certain value for all outcomes where an opposite positive or nonmonotone association was shown in univariable analysis. Hosmer-Lemeshow tests were conducted for all multivariate models for mortality and showed non-significant results (smallest p-value: 0.142).

**Fig 2:**
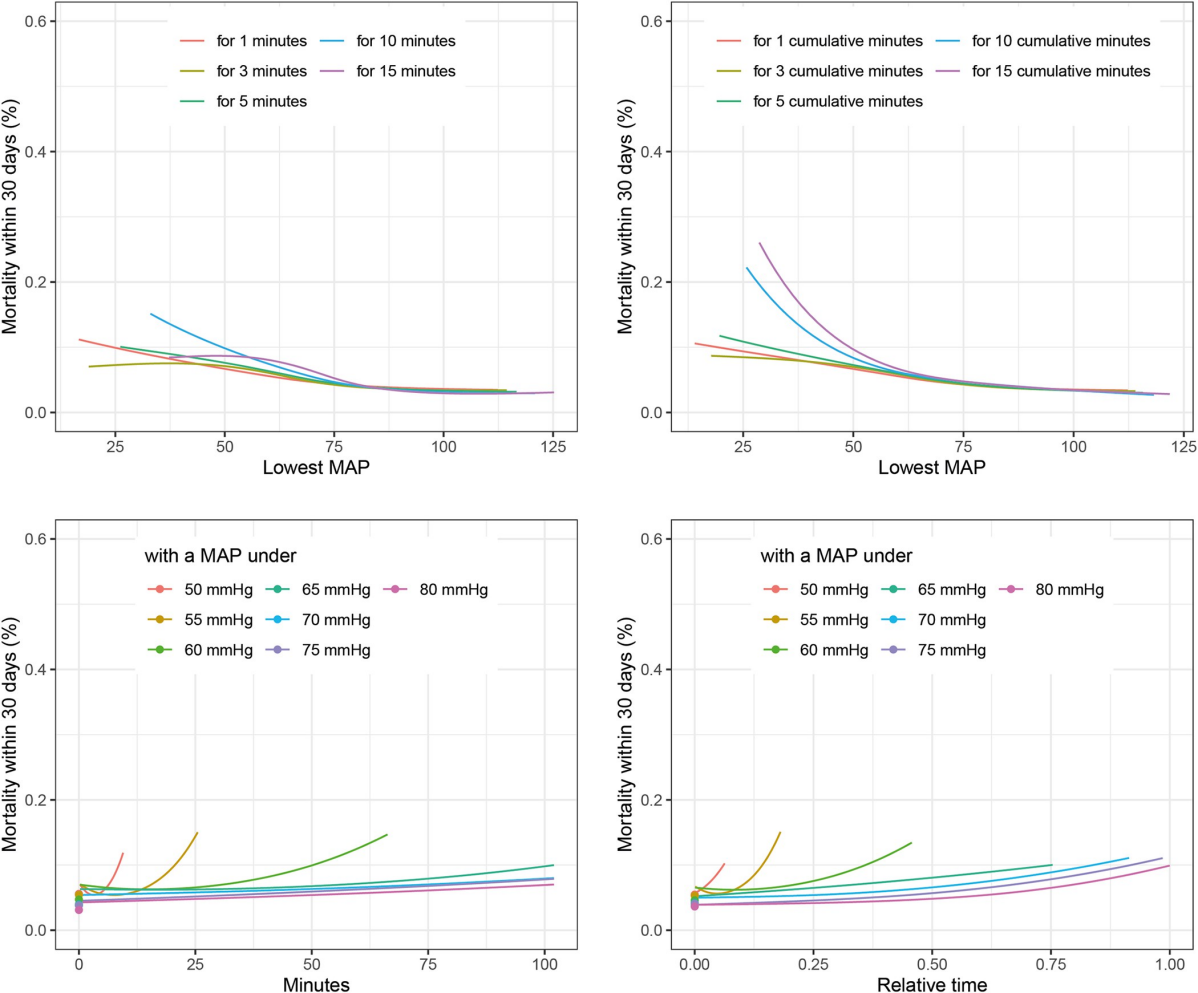
Association of blood pressure definitions with 30-day mortality. The figure shows the associations of different definitions of intraoperative hypotension with 30-day all-cause mortality. The average patient of the included cohort (Male, 53 years, BMI: 26, 1.7 h surgery, One day to surgery, Specialty: Urology, Gynecology or General surgery, Charlson morbidity score: 0, ASA 2) was used for adjustment. BMI: body mass index, MAP: mean arterial pressure.

Correcting for sex, age, BMI, duration of surgery, time from hospital admission to surgery, surgical specialty, ASA classification and comorbidity score showed similar trends as in the univariate analysis for most definitions of IOH (Fig 2). However, some definitions of IOH–especially absolute and relative time with a MAP below a certain level–showed no association. This is due to a long surgical duration being an important confounder for mortality and LOS.

Comparison of the different definitions of IOH using the Brier score and MSE showed different definitions of IOH that were best fitting for the three outcomes in the shaping dataset. Details can be found in Table 3. For 30-day mortality, “*Relative time with a MAP under 80 mmHg”* showed the best association in the shaping dataset. “*Lowest MAP for one minute”* had the highest association with hLOS, closely followed by “lowest MAP for one cumulative minute”. For PACU-LOS, we found the strongest association with *“lowest MAP for one cumulative minute”*, closely followed by *“lowest MAP for one minute”*. Brier scores and MSE values can be found in S1 File.

**Table 3:** Model fits.

	Mortality	LOS	PACU LOS
	**Brier**	MSE	MSE
**Lowest MAP sustained for**			
**1 minute**	0.0082492	**0.9545902**	0.3253831
**3 minutes**	0.0082482	0.9547002	0.3264418
**5 minutes**	0.0082515	0.9549230	0.3277762
**10 minutes**	0.0082444	0.9558962	0.3311165
**15 minutes**	0.0082391	0.9565503	0.3347769
**Lowest MAP sustained for**			
**1 cumulative minute**	0.0082489	0.9545948	**0.3252330**
**3 cumulative minutes**	0.0082503	0.9546291	0.3261551
**5 cumulative minutes**	0.0082524	0.9547680	0.3271016
**10 cumulative minutes**	0.0082537	0.9552866	0.3293193
**15 cumulative minutes**	0.0082543	0.9563053	0.3319592
**Absolute time [min] with a MAP**			
**under 50 mmHg**	0.0082653	0.9578553	0.3433382
**under 55 mmHg**	0.0082524	0.9575305	0.3430270
**under 60 mmHg**	0.0082468	0.9581017	0.3434005
**under 65 mmHg**	0.0082384	0.9583522	0.3428872
**under 70 mmHg**	0.0082369	0.9584708	0.3415926
**under 75 mmHg**	0.0082368	0.9579326	0.3384180
**under 80 mmHg**	0.0082416	0.9564761	0.3332404
**Relative time [min] with a MAP**			
**under 50 mmHg**	0.0082614	0.9580839	0.3440897
**under 55 mmHg**	0.0082490	0.9579228	0.3438590
**under 60 mmHg**	0.0082452	0.9583299	0.3438186
**under 65 mmHg**	0.0082471	0.9583150	0.3433043
**under 70 mmHg**	0.0082462	0.9580672	0.3417224
**under 75 mmHg**	0.0082373	0.9575332	0.3385582
**under 80 mmHg**	**0.0082359**	0.9563751	0.3331340

Best performing definitions are marked in bold. LOS: Length of stay, PACU: Post anesthesia care unit, LOS: length of stay, PACU: postoperative care unit, MAP: mean arterial pressure, IOH: intraoperative hypotension, min: minutes

**Fig 3:**
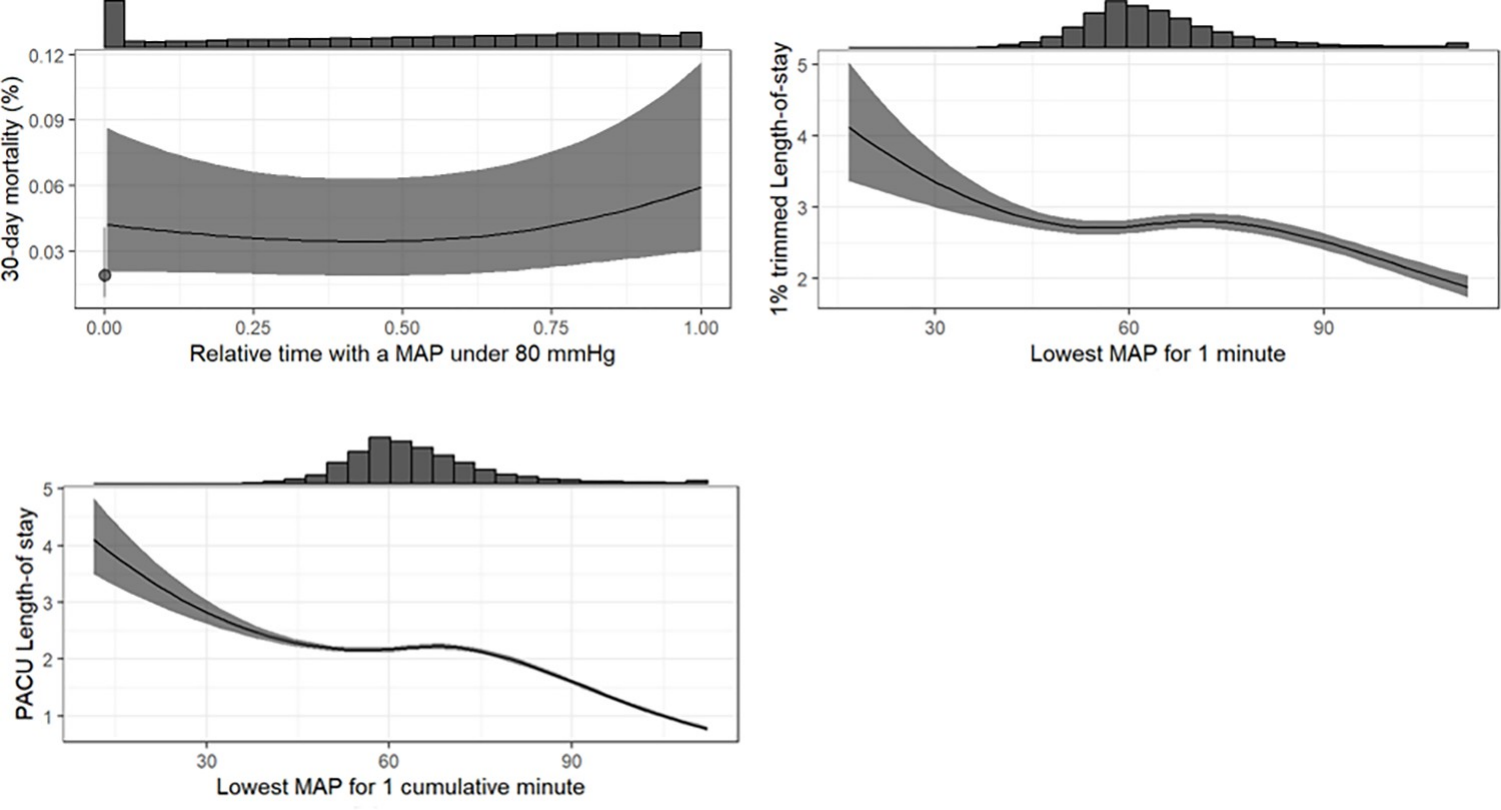
Selected characterizations in estimation dataset. The figure shows the selected characterizations of IOH in the estimation dataset including confidence intervals. MAP: mean arterial pressure, PACU: postanesthesia care unit.

**Fig 4:**
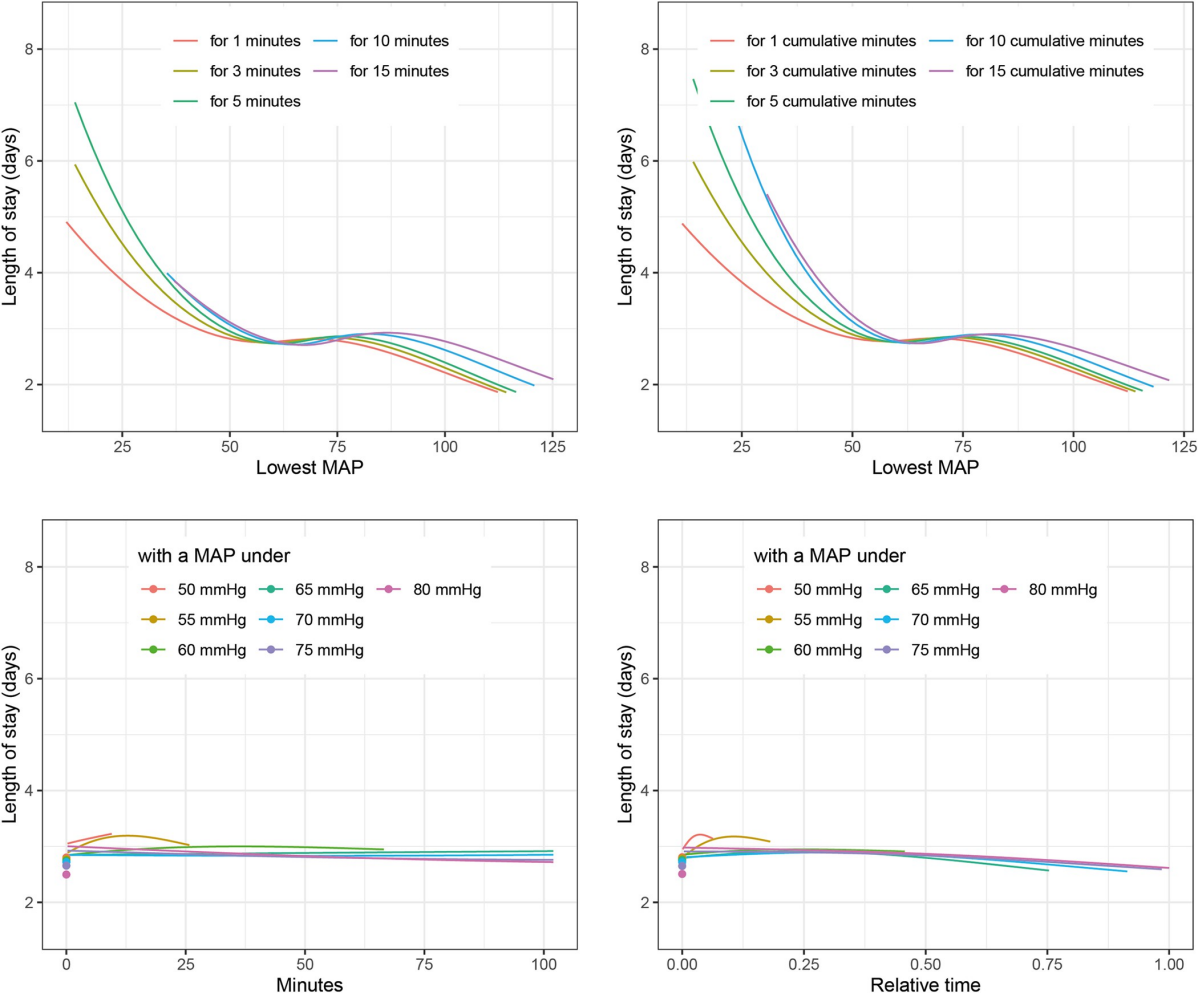
Association of blood pressure definitions with hospital length of stay. The figure shows the associations of different characteristics of intraoperative hypotension with hospital length of stay. The average patient of the included cohort (Male, 53 years, BMI: 26, 1.7 h surgery, One day to surgery, Specialty: Urology, Gynecology or General surgery, Charlson morbidity score: 0, ASA 2) was used for adjustment. BMI: body mass index, MAP: mean arterial pressure.

**Fig 5:**
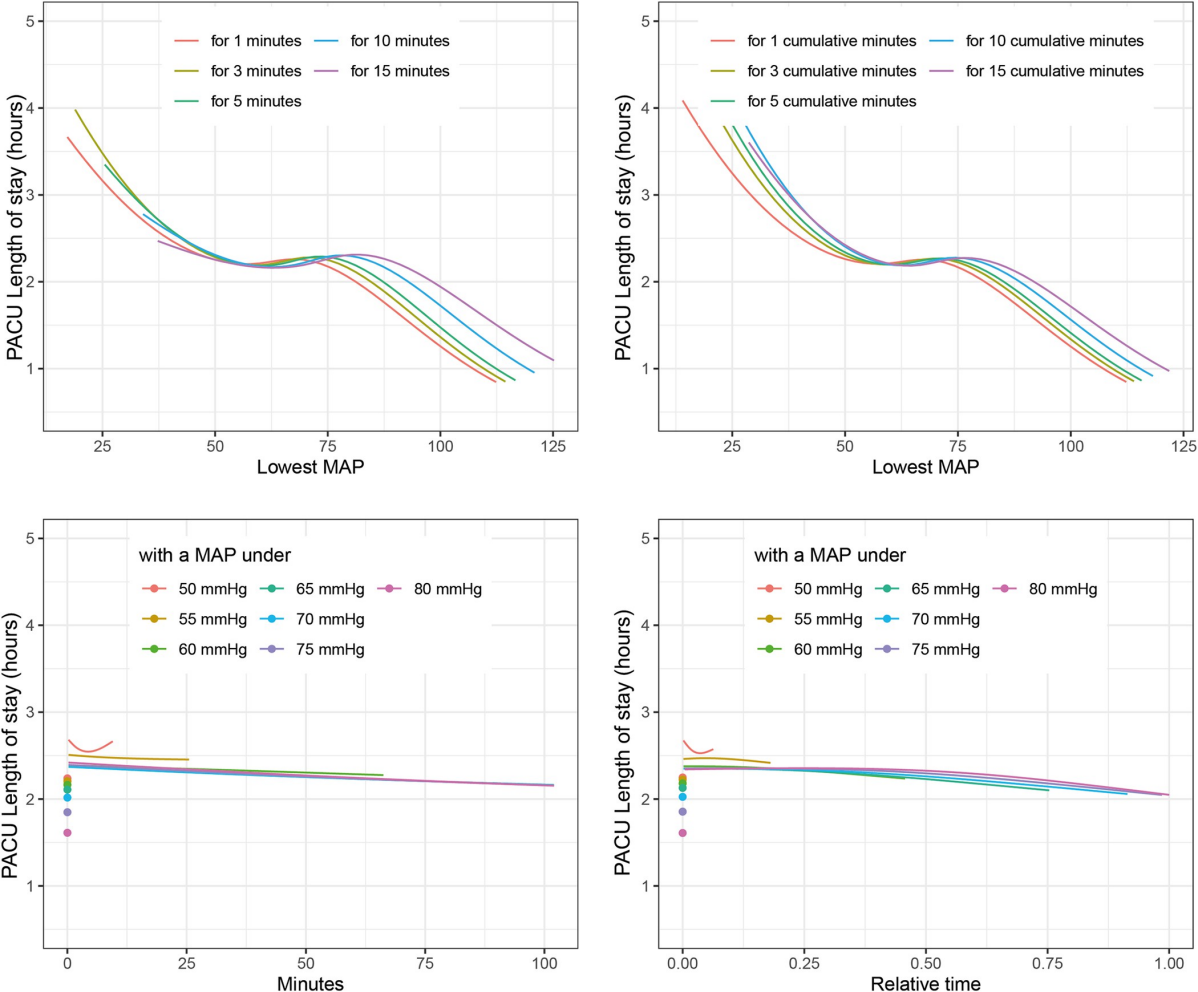
Association of blood pressure definitions with PACU length of stay. The figure shows associations of different definitions of intraoperative hypotension with postanesthesia care unit length of stay. The average patient of the included cohort (Male, 53 years, BMI: 26, 1.7 h surgery, One day to surgery, Specialty: Urology, Gynecology or General surgery, Charlson morbidity score: 0, ASA 2) was used for adjustment. BMI: body mass index, MAP: mean arterial pressure, PACU: postanesthesia care unit.

Testing these best fitting definitions of IOH in the estimation dataset using the above-described multivariable analysis showed associations between all three outcomes and IOH. (Fig 3) The risk of 30-day mortality was lowest when the MAP during surgery was above 80 mmHg. When the MAP was below 80 mmHg for more than 50% of the duration of surgery, the risk of 30-day mortality increased steadily with every percent point of time below 80 mmHg. (Fig 2). The length of hospital stay showed a sigmoid curve with the highest LOS values in those with a *“lowest MAP for 1 minute”* below 45 mmHg, reaching a plateau between 50 and 75 mmHg before further decreasing for higher values of *“lowest MAP for 1 minute”* (Fig 4). *The* PACU-LOS showed very similar behavior but for *“lowest MAP for 1 cumulative minute”* (Fig 5).

Effect sizes and confidence intervals were calculated for clinically relevant values of MAP (50mmHg, 65mmHg, 75mmHg) and time (5 & 10 minutes) as well as 5% & 10% of time. (S2.1-S2.3 Tables in S2 File). In addition to evaluating the association of IOH with mortality, hLOS, and PACU-LOS, we assessed the degree of influence of IOH and adjustment variables on these outcomes using the Brier score and MSE. While mortality and hLOS were mainly influenced by the ASA score (mortality) and duration of surgery (hLOS), respectively, PACU LOS was mostly influenced by duration of surgery and IOH.

Interactions between age and ASA status with the chosen IOH definition was calculated and showed no significant association for mortality. Regarding hLOS and PACU-LOS differences could be shown. (S2.5 & S2.6 Figs in S2 File).

The 0.95 confidence set on the model for mortality contains eight models: Beside the selected Relative time with a MAP under 80mmHg also Relative time with a MAP under 75mmHg, Absolute time with a MAP under 80 mmHg, Absolute time with a MAP under 75 mmHg, Absolute time with a MAP under 70 mmHg, Absolute time with a MAP under 65 mmHg, Lowest MAP for 15 minutes and Lowest MAP for 10 minutes are within the 0.95 confidence set. In contrast, the 0.95 confidence set for hLOS contains only three IOH definitions (Lowest MAP for 1 minute, Lowest MAP for 1 cumulative minute, and Lowest MAP for 3 cumulative minutes) and that for PACU-LOS contains only the selected Lowest MAP for 1 cumulative minute.

The conducted sensitivity analyses showed only small changes in the characterizations’ order proving the stability of the shown results: Changing from 1% to 1.5% or 2% trimming for hLOS lead to “Lowest MAP for one cumulative minute” being ranked first place (second in main analysis) and “Lowest MAP for one minute” second (first in main analysis). Regarding mortality the sensitivity analysis using four degrees of freedom instead of three for the spline fit of IOH definitions showed larger deviations: Using 3 degrees of freedom, “Relative time with a MAP under 80” was the best fitting characterization; “Lowest MAP for 15 minutes” ranked 6th place. Using 4dfs “Lowest MAP for 15 minutes” performed best and “Relative time with a MAP under 80” is 4th place.

## Discussion

In this trial, it was showed that most definitions of IOH are associated with all three investigated outcomes. Mortality and length of stay are endpoints influenced by many factors. Intraoperative hypotension seems to be one of those factors for mortality, as described in previous trials [12, 14, 30]. A recent meta-analysis evaluating 29 trials with a total of 130,862 patients showed that IOH increased mortality with an odds ratio of nearly 2. However, the absence of a universal definition of hypotension complicated the analysis [19, 20]. This demonstrates the need for a data-driven selection of a best fitting definition of IOH, which we present in the current study. Therefore, we equally split our data into a shaping dataset and an estimation dataset. After identifying the best fitting definitions in the shaping dataset, we tested their performance in the estimation dataset. A wide variety of IOH definitions was studied including some, that might not even be considered hypotensive by regular standards or only in patients, that had existing hypertensive pathologies.

In the used cohort of non-cardiothoracic patients, we found that “*Relative time with a MAP under 80 mmHg”* was the best fitting definition of IOH regarding mortality. Importantly, our findings suggest a much higher cutoff MAP value for mortality discrimination and therefore contradicts the 55 and 65 mmHg reported in previous studies [1, 5, 12]. This “normotensive” definition of IOH–originally added to the analysis to test currently used definitions–raises questions regarding causality. It is important to keep in mind, that due to the retrospective nature of this study, only an association can be shown rather than a clear cause and effect relationship. Likely, at least this characterization of IOH is not a direct risk factor for mortality, but a factor associated with mortality. Another factor pointing in that direction is that more severe hypotension (ie. time with a MAP < 55mmHg) was associated less with mortality than “Relative time with a MAP under 80 mmHg”. The inclusion of patients with a history of hypertension could be another limitation: Some authors suggest personalized blood pressure thresholds. Although the Charlson comorbidity score was included into the model, this potential bias cannot be excluded. Another factor suggesting that unmeasured factors might have a significant impact is the small effect size regarding all outcomes.

When investigating the Brier score to test the variables influencing the model most, ASA classification is the most influential parameter, whereas IOH seems to be equally influential as patients’ age or BMI. The Brier score has been chosen a priori as selection criterion based on our experiences in our previous work where similar selection results were obtained [21, 31]. The MSE for the continuous LOS outcomes was then chosen as the natural counterpart to the Brier score for this type of outcome. While age and BMI cannot be modified by the treating anesthesiologists, IOH can be prevented. However, randomized prospective trials predicting IOH showed no differences in outcome [32–35]. This poses the interesting question of whether the association of IOH with worse outcomes is, at least to some extent, an epiphenomenon of patient condition and comorbidity. In contrast to mortality, the association of IOH with LOS has been investigated in only a few studies–mostly in special patient populations such as those receiving hip surgery or abdominal surgery [14, 17, 36]. Again, a consensus on the definition of IOH is missing, especially in a general surgical population. Data on the association of IOH and PACU-LOS were missing entirely to the authors’ best knowledge.

Recently, a meta-analysis investigating the influence of intraoperative hypotension an postoperative outcomes was published including ten prospective randomized trials [15]. The authors could show, that there is no influence of IOH on mortality. Of note, the meta-analysis included cardiac and non-cardiac surgery showing even a small but significant reduction of LOS in the non-cardiac group. The authors introduce the concept of protective intraoperative hemodynamics and hypothesize that treatment of IOH could worsen outcomes by introducing other harmful side effects. The present trial further supports the idea of associations of IOH and outcome being an epiphenomenon to a certain extent.

Regarding our LOS analyses, we found *Lowest MAP for one minute* to be the best fitting IOH definition for hLOS and *“Lowest MAP for one cumulative minute”* to be the best fitting IOH definition for PACU-LOS. In contrast, most previous studies used a minimum duration threshold of up to 15 minutes to define hypotensive episodes [19].

When looking at the Brier Scores and MSE in detail, only small differences within one category of IOH definition (i.e. Time with MAP below 1, 3, or 5 minutes) could be found. In contrast good discrimination was seen between categories of IOH definitions (Time with MAP below vs. MAP for x minutes). Therefore, it seems to be more important which IOH definition is used rather than what exact threshold to choose.

While IOH had less influence than length of surgery and duration from hospital admission to surgery on hLOS, it had as much influence as length of surgery on PACU-LOS. Thus, we hypothesize that, in addition to the known mid-term effects, such as kidney injury and myocardial injury, IOH might trigger both short-term and medium-term effects, such as postoperative nausea and vomiting (PONV), delirium, and pain [20, 37, 38].

Multivariable regression analyses revealed a sigmoid relationship between IOH and both hLOS and PACU-LOS (Figs 4 and 5). These results showing further improvement of postoperative outcomes even after reaching levels of normotension may seem surprising, but are in line with previously published studies showing at least no correlation of moderate hypertension and postoperative outcomes [6, 12, 39]. Possibly this may be another indication that the association of IOH with postoperative outcome is to a certain, yet unknown extent more of an epiphenomenon than an immediate causality. Future–prospective–studies will have to proof clear causalities and research if a time with a MAP below 80mmHg maybe a new definition of IOH.

In this study, we found a three-phase association of IOH with postoperative outcomes as follows: 1) below a MAP of approximately 60 mmHg, hLOS and PACU-LOS decreased with increasing MAP, followed by 2) a plateau between 60 and 75 mmHg, and 3) above a MAP of 75 mmHg, hLOS and PACU-LOS further decreased with increasing MAP. This negative sigmoid association of intraoperative MAP with outcomes is in line with recent, seemingly conflicting, studies. Some found associations of an intraoperative MAP lower than 55 to 65 mmHg with worse postoperative outcomes [5, 6, 40, 41], whereas others reported a much higher cutoff of 75 to 80 mmHg to be associated with worse postoperative outcomes [1]. When focusing on 30-day mortality, we did not observe this sigmoid effect. However, we report a continuous increase in the risk of 30-day mortality with decreasing MAP, leading to “*Relative time with a MAP under* 80 mmHg” as the definition most associated with this endpoint. Taken together, our findings emphasize the importance of a proper selection of IOH definitions.

### Limitations

The most obvious limitation of this trial is the small effect size regarding all outcomes: When looking into these results in further detail, one must assume that both length of stay and mortality are influence by many factors. As surgical performance can hardly be quantified retrospectively, no correction for this huge confounder is possible. Therefore, it is obvious, that IOH only has limited effect compared to other factors. Although this trial included a large number of patients, it was conducted at a single tertiary academic center, thus limiting generalizability to other settings or populations. Differences in patient populations, but also in surgical practices and perioperative management may introduce bias when applying the results to other centers. Especially the exclusion of certain patient groups like emergency surgery or cardiothoracic surgeries has to be taken into consideration. However, inclusion of these special populations would have potentially skewed the results.

It was decided to include only the first of possible subsequent surgeries, but not to exclude patients receiving multiple surgeries in general. This was done as subsequent surgeries in one admission typically have to be seen as complication with a possible influence on mortality and length of stay. Therefore, exclusion of those patients would have posed a risk of bias.

In this study, we used MAP values and a set of characterizations of IOH taken from previous literature to define intraoperative hypotension [6, 42]. This could lead to misclassification, especially in patients requiring individualized blood pressure targets. As the available information about preexisting arterial hypertension in our database was very limited and also unequally distributed regarding details like extent and treatment of hypertension, it was decided to not include those data in the models to avoid the introduction of potential additional bias. Furthermore, usage of absolute MAP thresholds is common in literature [20, 41, 42].

Artifact cleaning is a crucial point when conducting retrospective research in anesthesia as it was shown that the choice of artifact filtering method can alter results [23]. We chose a cut-off filter that has the highest sensitivity in MAP [43].

Handling missing data can introduce bias, and while multiple imputation may provide an unbiased estimate, it can also introduce inaccuracy. However, as only 1% of cases were excluded to missing data (Fig 1, S2.4 Table in S2 File) a measurable improvement of accuracy or bias reduction is not to be expected. Although the analysis was adjusted for several confounding variables, some residual confounding factors remain like fluid balance or vasopressor use introducing a certain degree of bias. The effect of the various treatment options for hypotension on patient outcome is a very interesting topic in itself as this would indicate to which extent intraoperative hypotension is an epiphenomenon or a modifiable factor.

Another possible limitation of the approach of the present methodology is overfitting: Despite our data-splitting approach and the use of cross-validation techniques, generalizability to different settings (especially centers with other case-mixes) may thus be limited to some extent. Prospective trials and external validation need to confirm the presented findings. The low duration of IOH reflects a high awareness in the treating staff further limiting the effect size. Furthermore, collecting data about patient death is challenging, especially when extending beyond hospital discharge. We therefore used data from the Federal Statistical Office, including all patients who died within the state borders. Therefore, patients who were transferred to other countries or died abroad could not be identified using this method. Another possible limitation is the merging of the two databases; patients were identified by their social security number (including date of birth) and names, which theoretically excluded the possibility of errors. However, some patients had to be excluded due to unrealistic dates of death. By rigorous data cleaning and checking for inconsistencies, we tried to prevent these possible flaws. Despite all those efforts, possible missing data or errors in manual entries by the healthcare professionals remain a possible source of error. Furthermore, a causal interpretation of our results is conditional on the assumption that all relevant confounders were included in our models.

### Conclusion

In this retrospective trial, *“Cumulative time with a MAP <80 mmHg”* was the best fitting definition of intraoperative hypotension for associations with mortality, while *“Lowest MAP for one minute”* performed best for hospital length of stay, and *“Lowest MAP for one cumulative minute”* for PACU-length of stay. All these definitions showed an association with the outcome in question when tested in an independent dataset. Our findings demonstrate that the selection of the best fitting definition of IOH strongly depends on the investigated outcome. This can direct selection of IOH definitions for future research which has to show if this found association is based on a causation or is merely an epiphenomenon; an information needed by clinicians to further improve their patient care.

## Supporting information

S1 File Performance of all IOH characterizations. (DOCX)

S2 File Effect sizes, sensitivity analysis, missing information. (DOCX)

S3 File STROBE checklist. (DOCX)

## Abbreviations

IOH: intraoperative hypotension
hLOS: hospital length of stay
PACU-LOS: postanesthesia care unit length of sta
MAP: mean arterial pressure
ICU: intensive care unit
IQR: inter-quartile range
SD: standard deviation
BMI: body mass index
ASA: American society of anesthesiologists
