# Supplementary material for: Definition of clinically relevant intraoperative hypotension: A data-driven approach
Source: PLoS One. 2024 Nov 1;19(11):e0312966. doi: 10.1371/journal.pone.0312966 (PMC11530086; doi:10.1371/journal.pone.0312966)

# Supplementary material 1

This supplement shows all uni- and multivariable models including Brier scores and MSE respectively. P<0.0001 is shown as p=0, histograms on top of the curves show the distribution of values, grey areas indicate 95% confidence intervals.

## Mortality

Plots of uni- and multivariate modells in the shaping dataset:


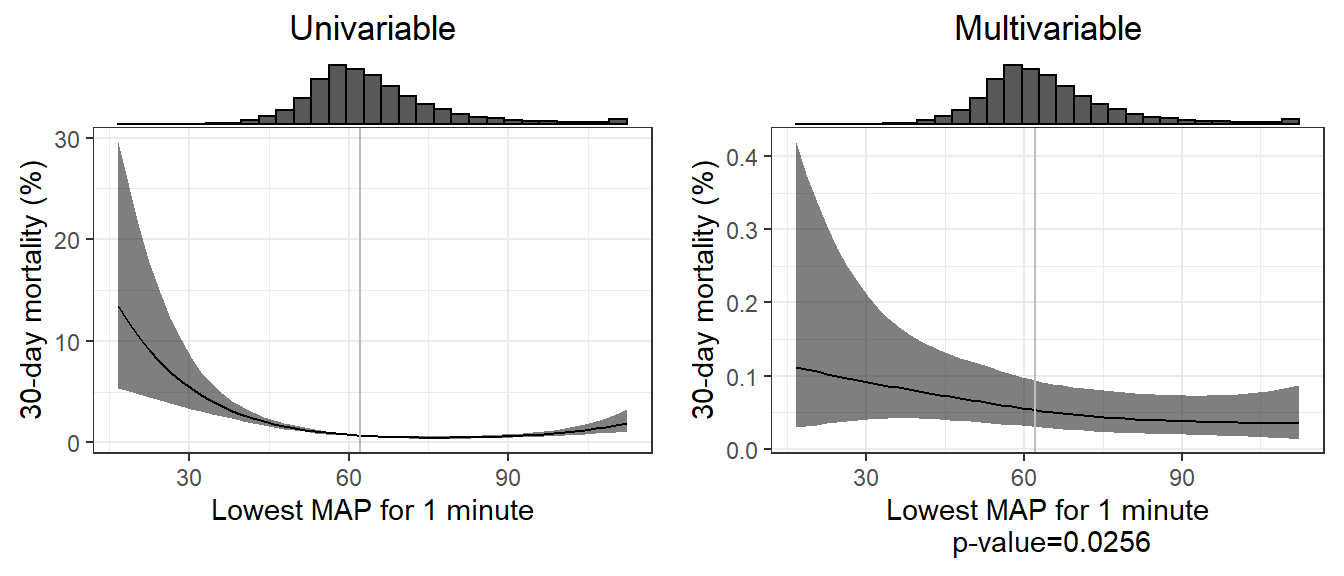

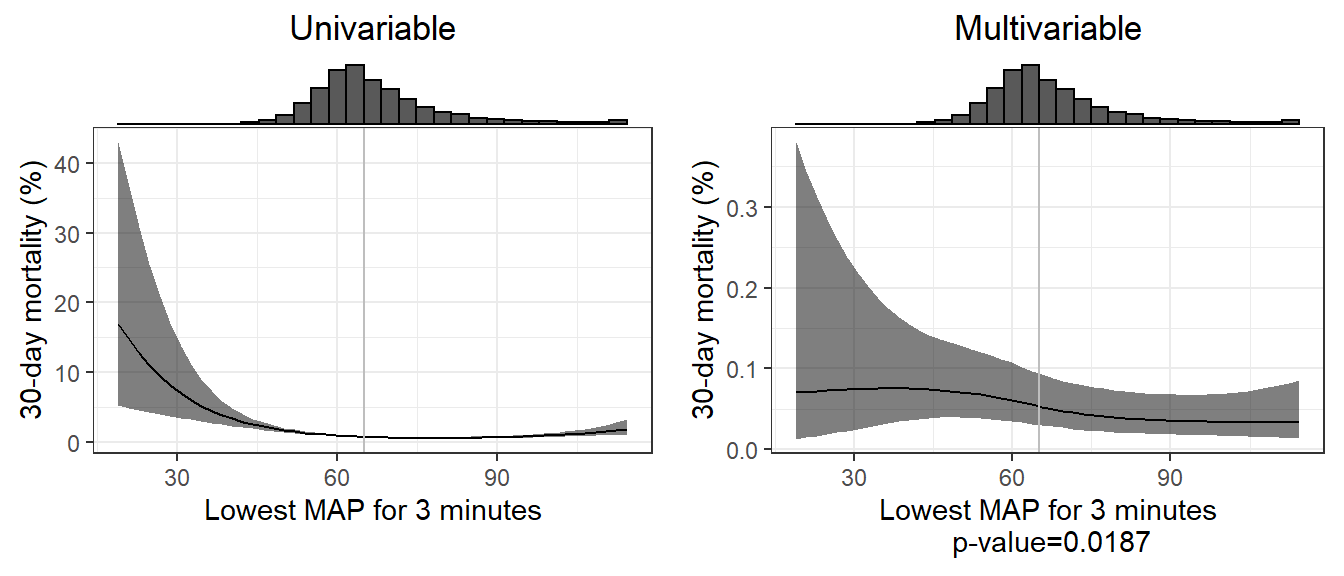

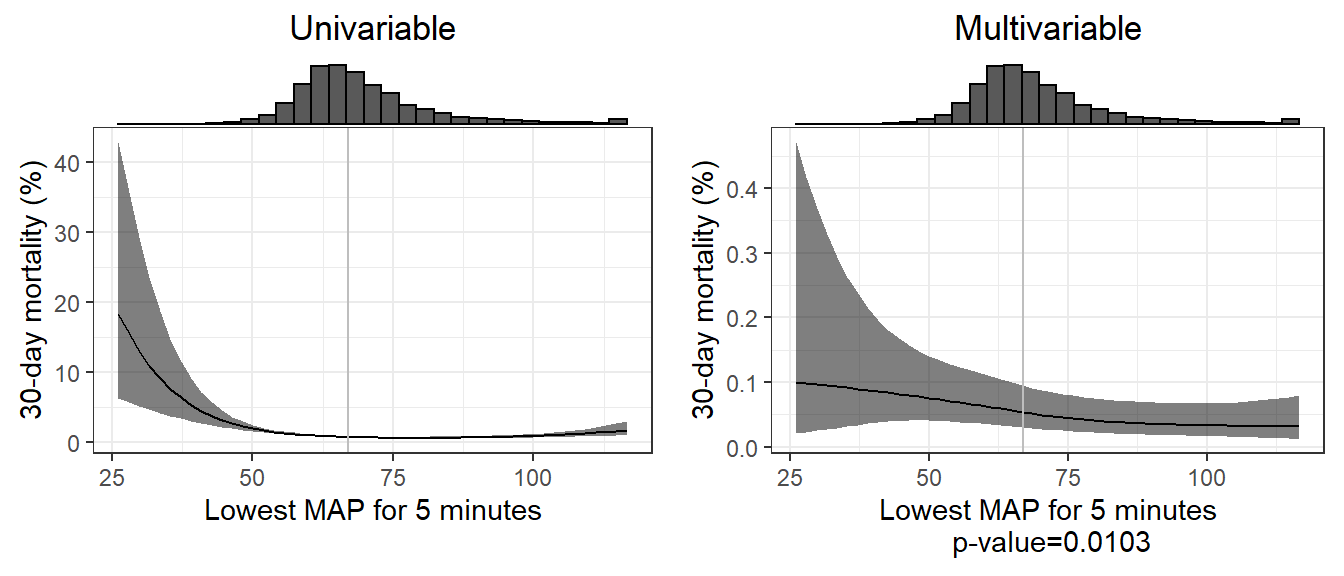

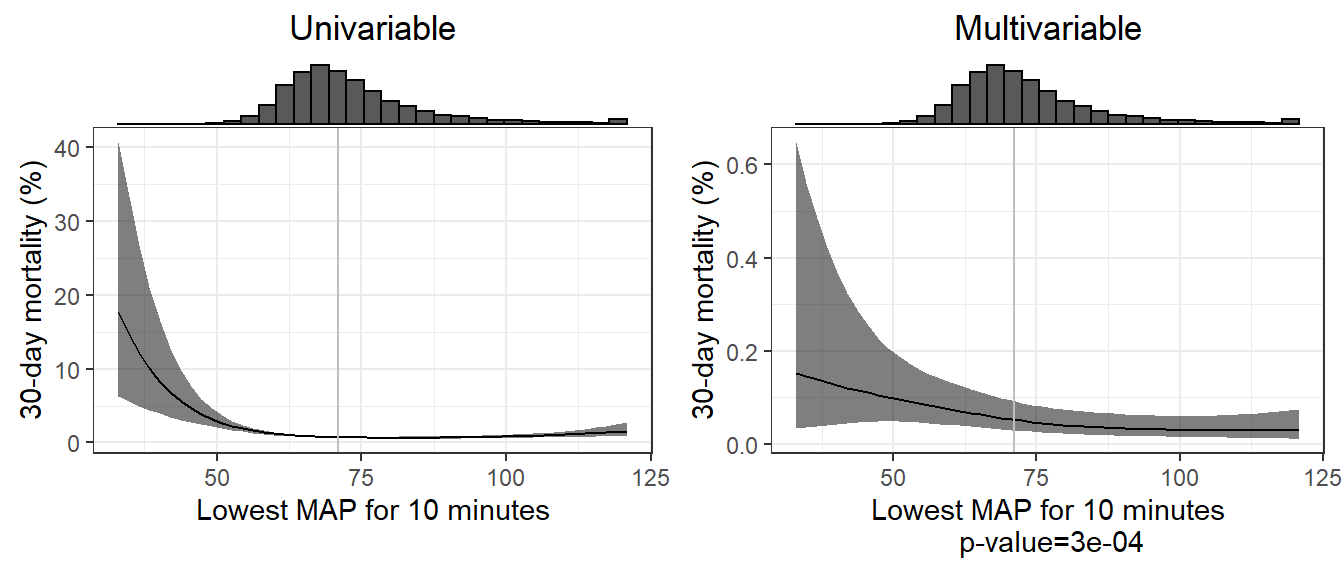

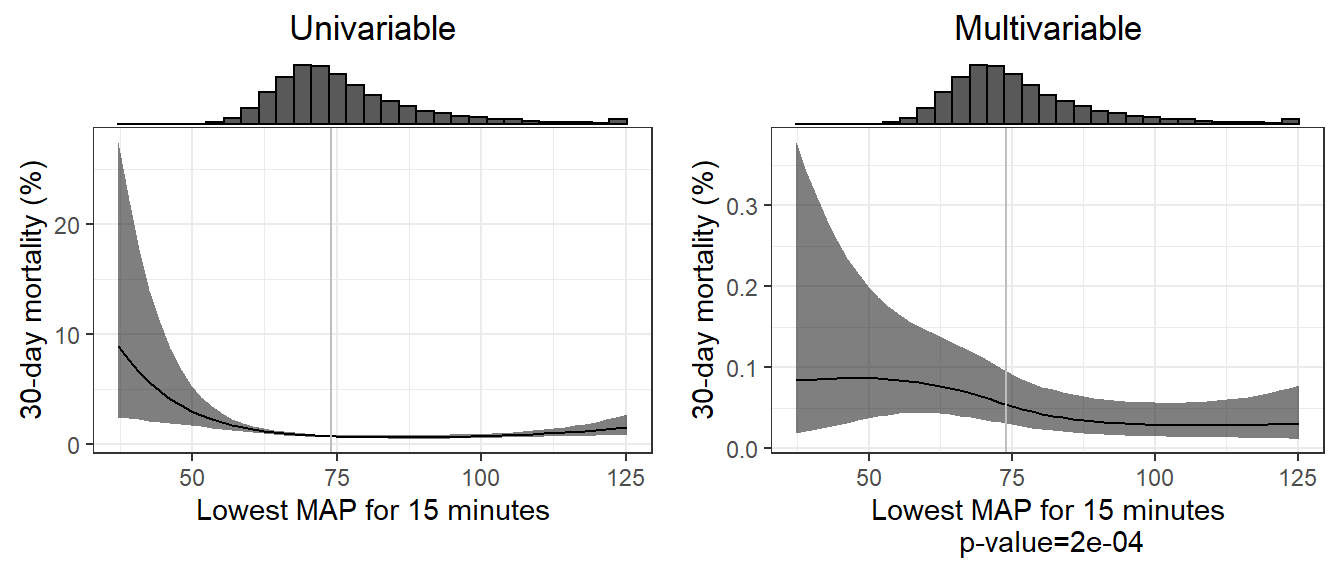

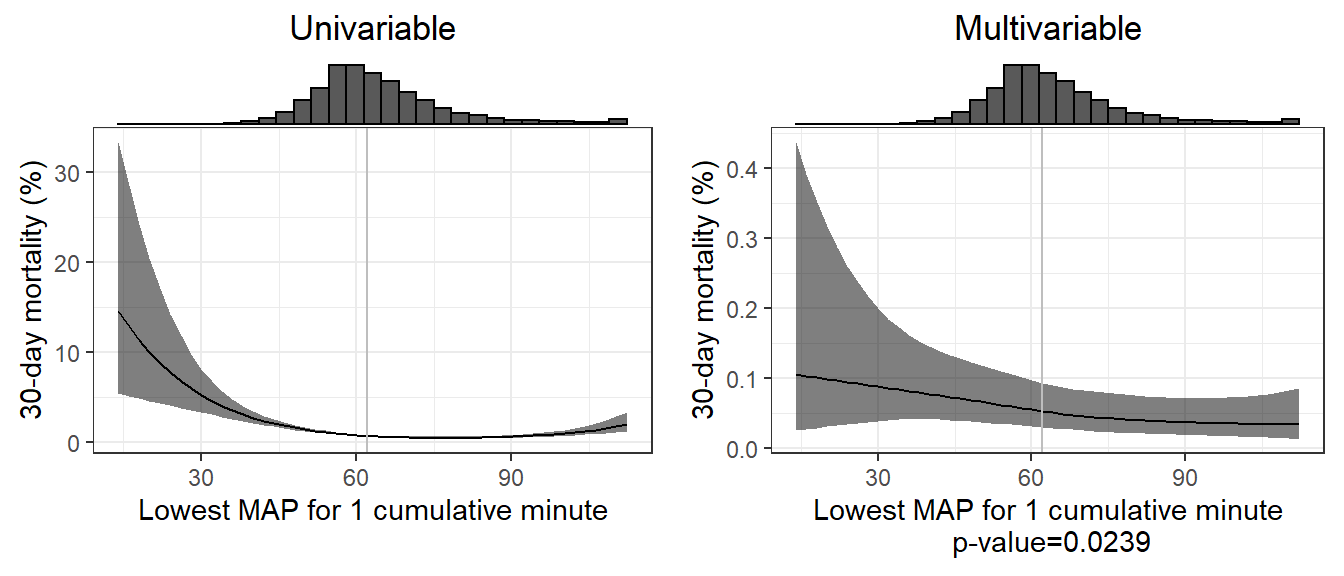

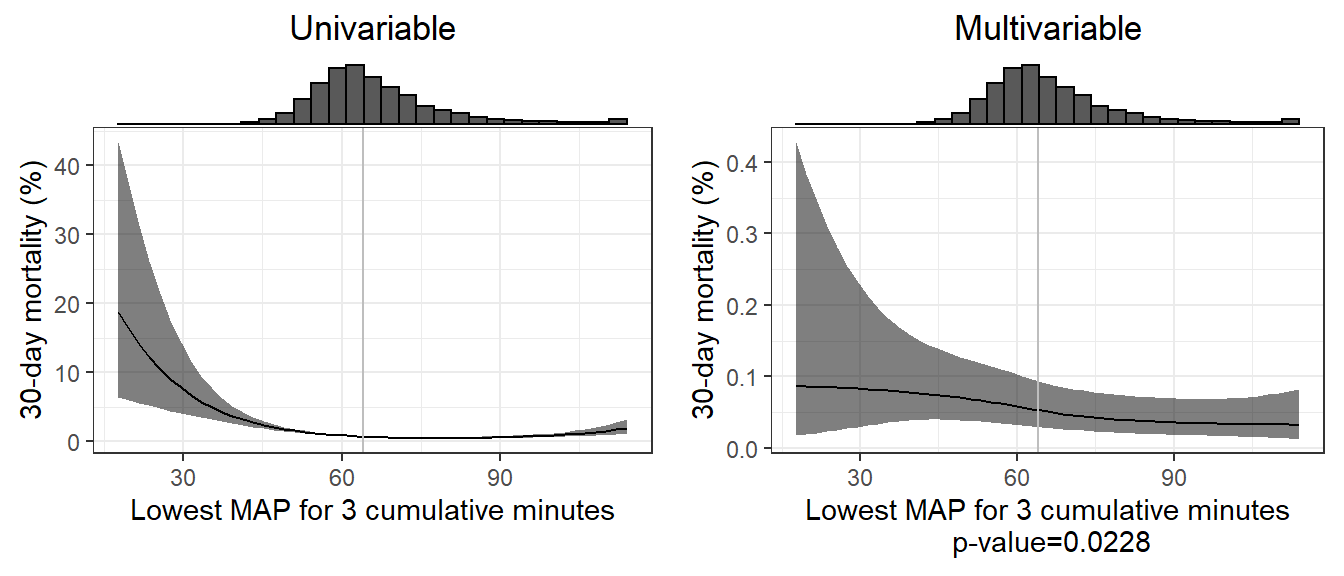

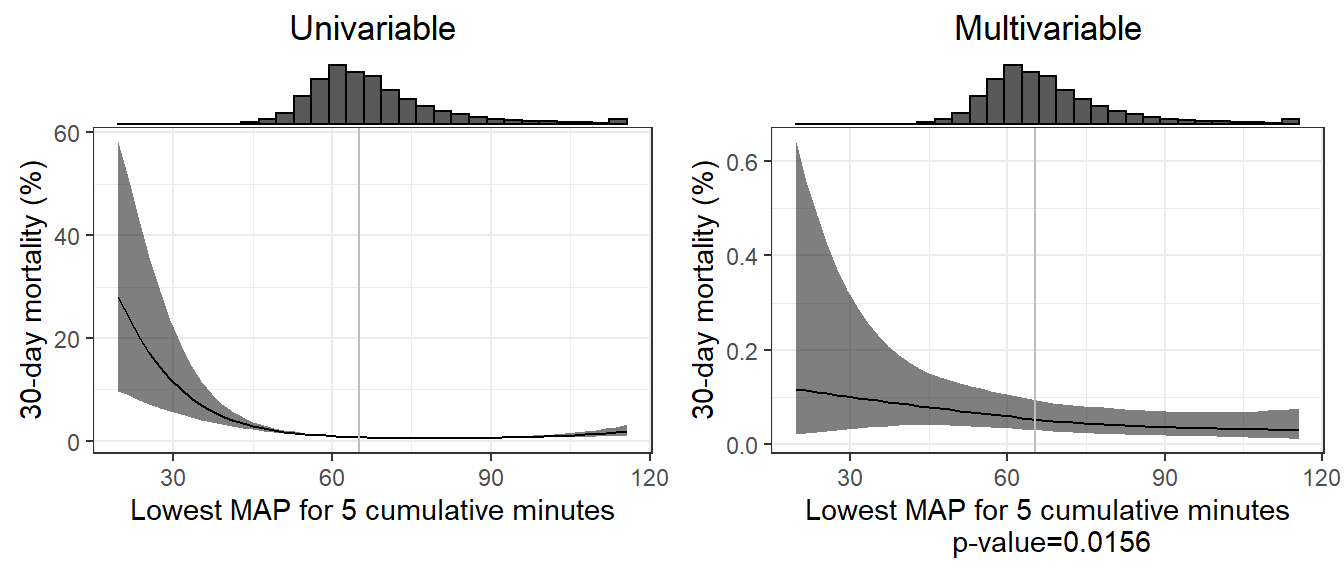

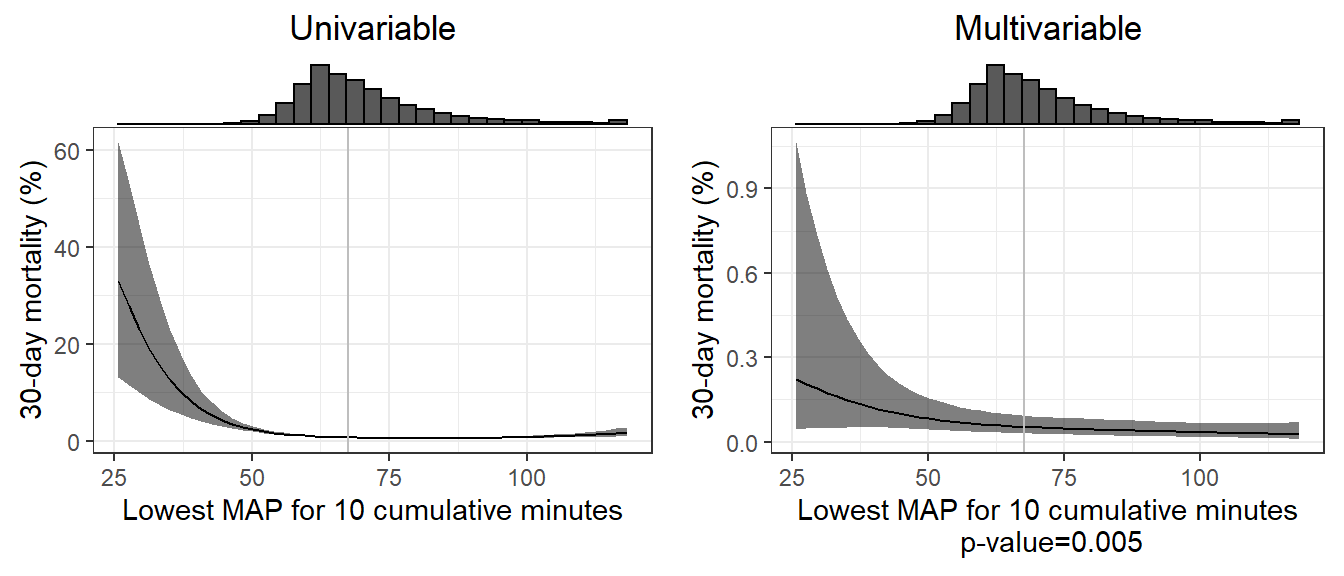

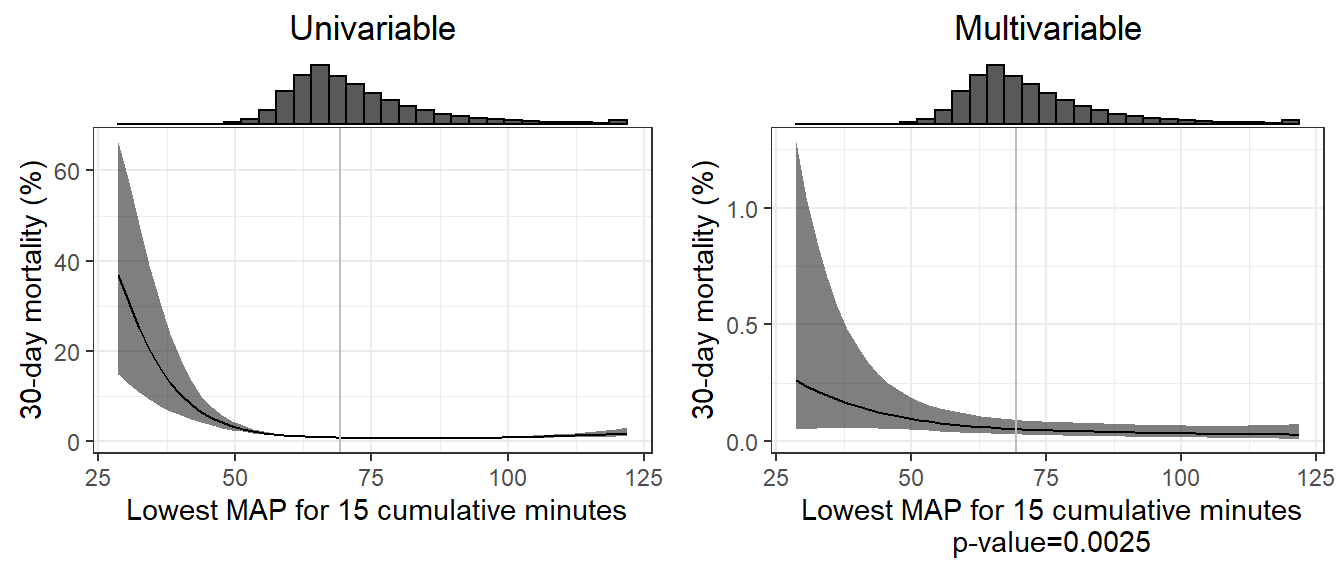

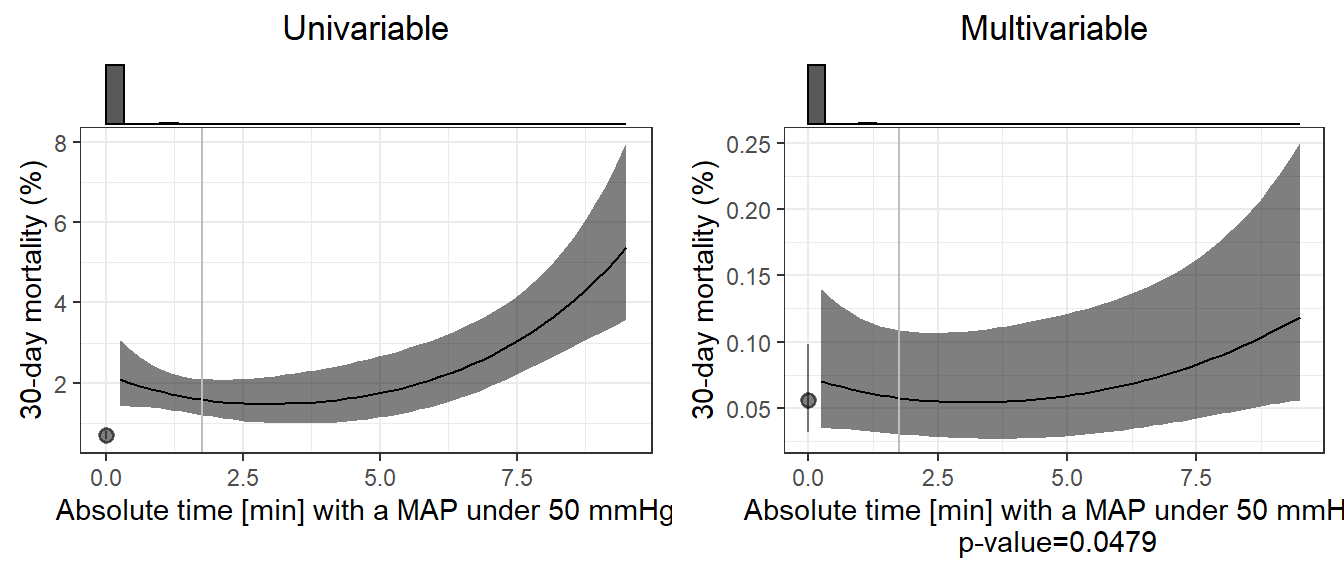

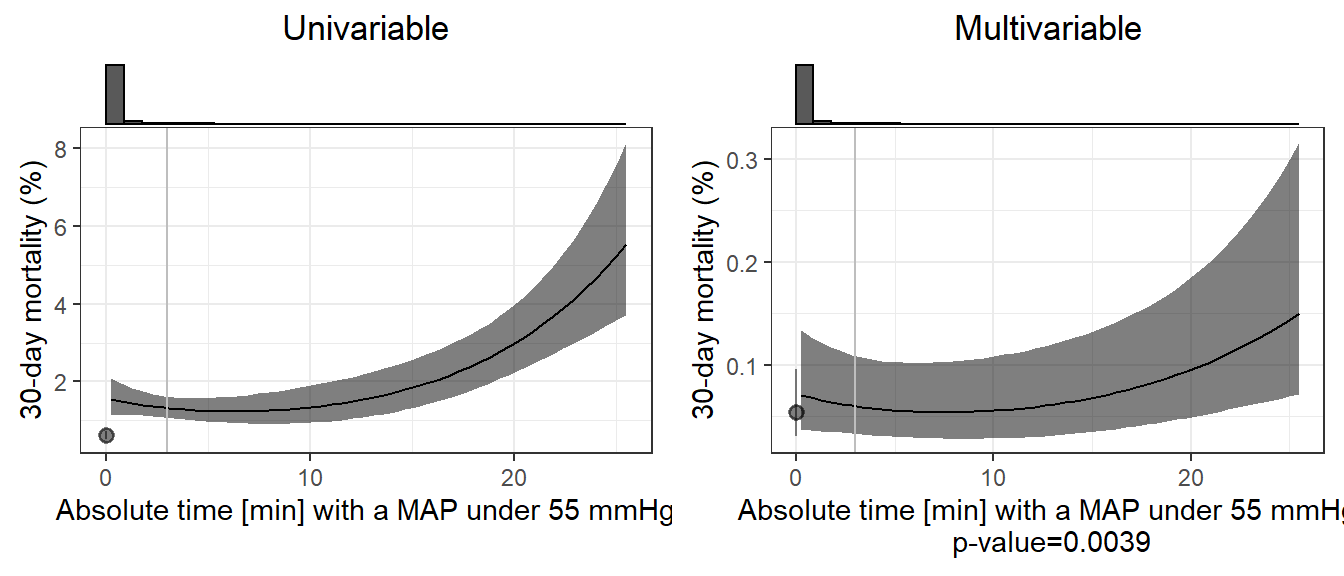

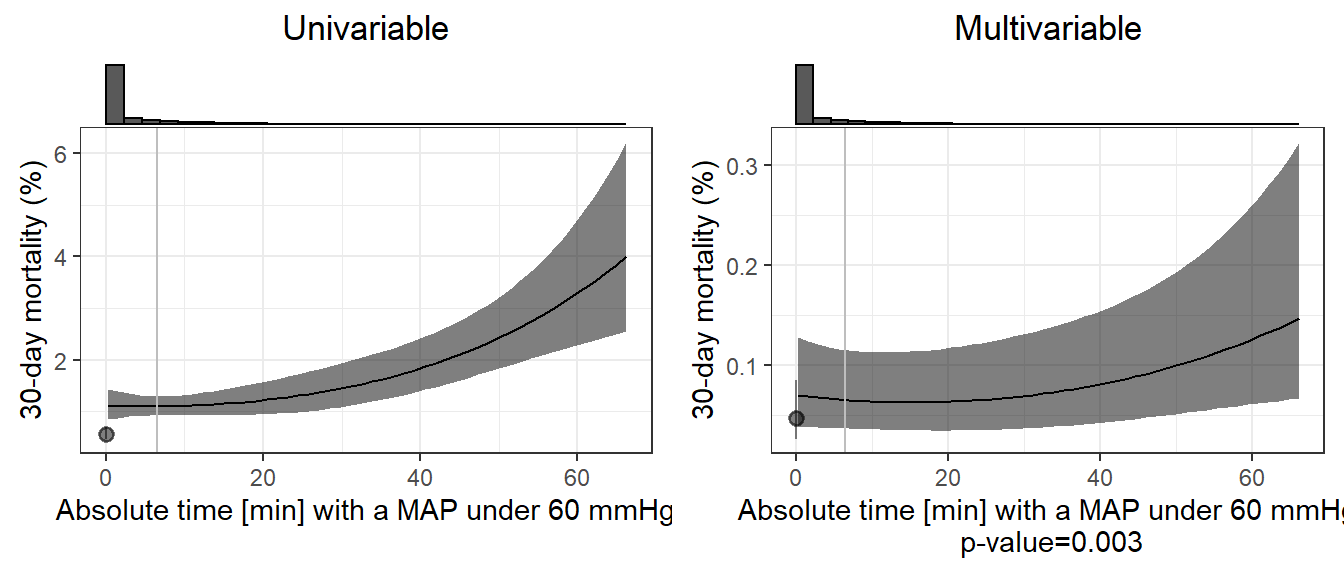

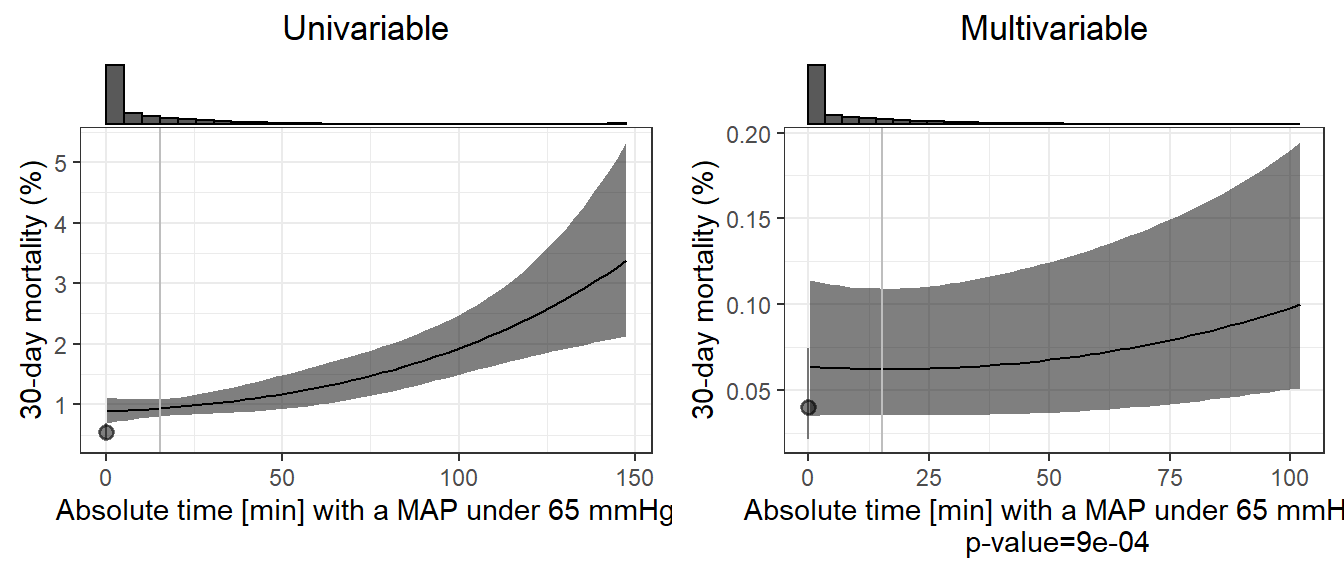

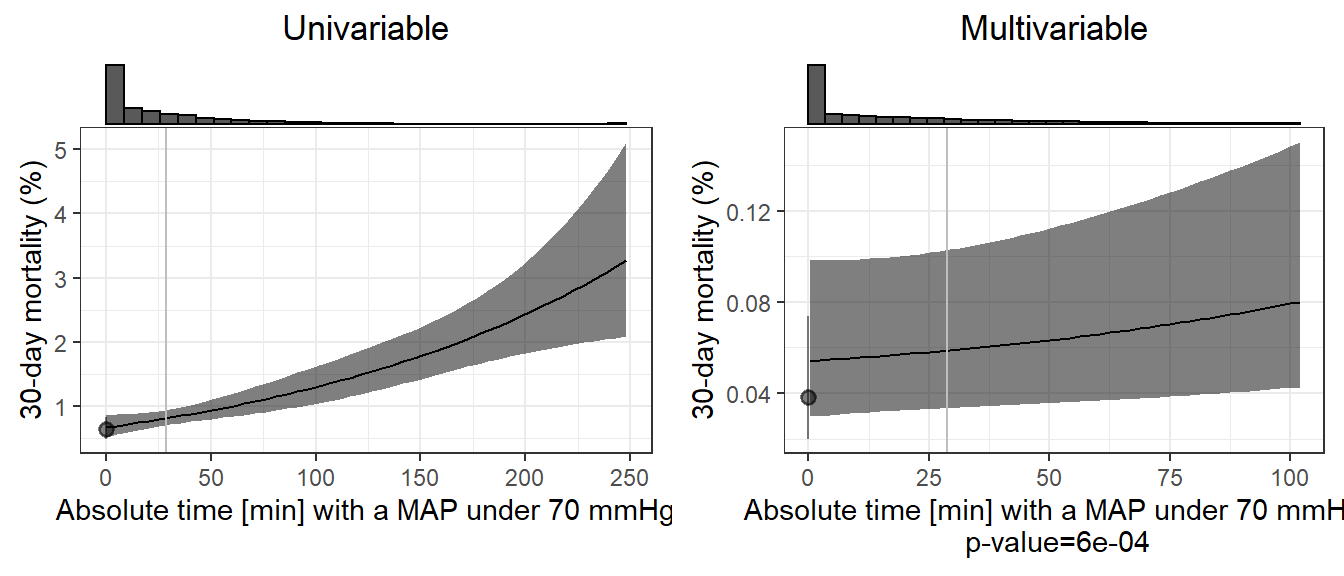

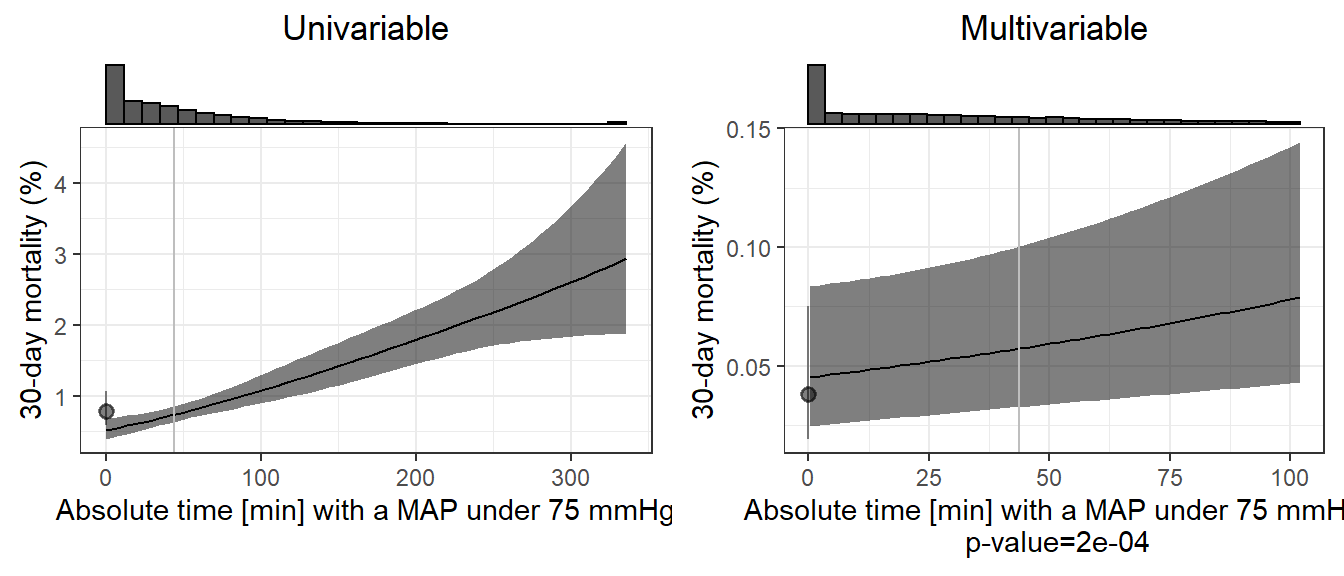

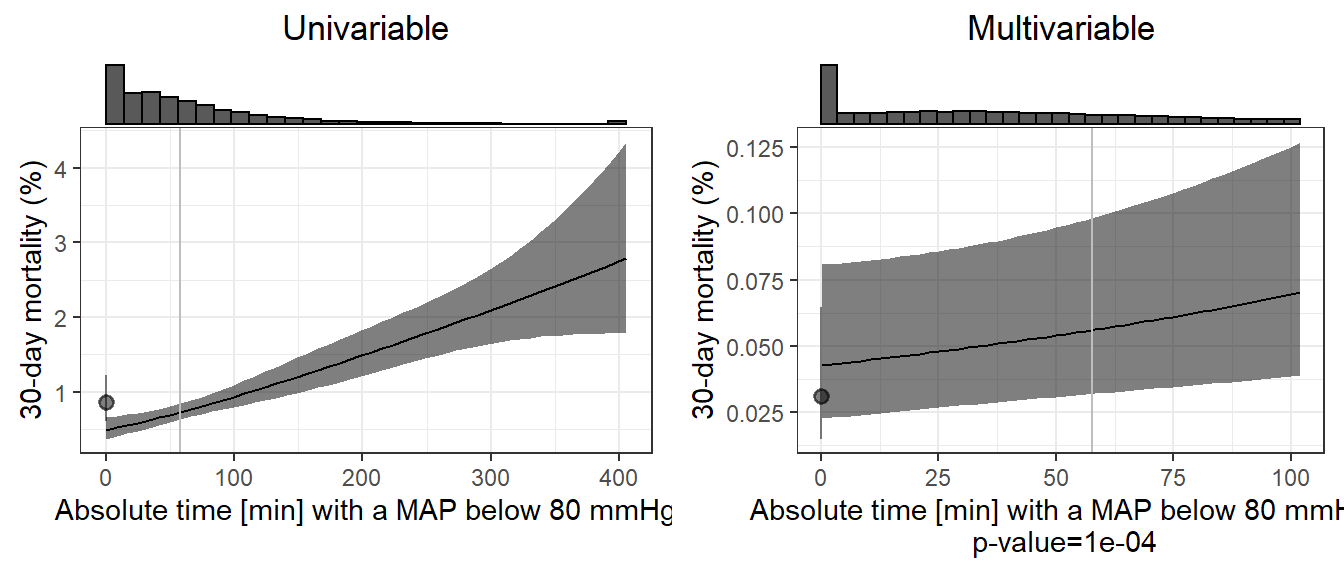

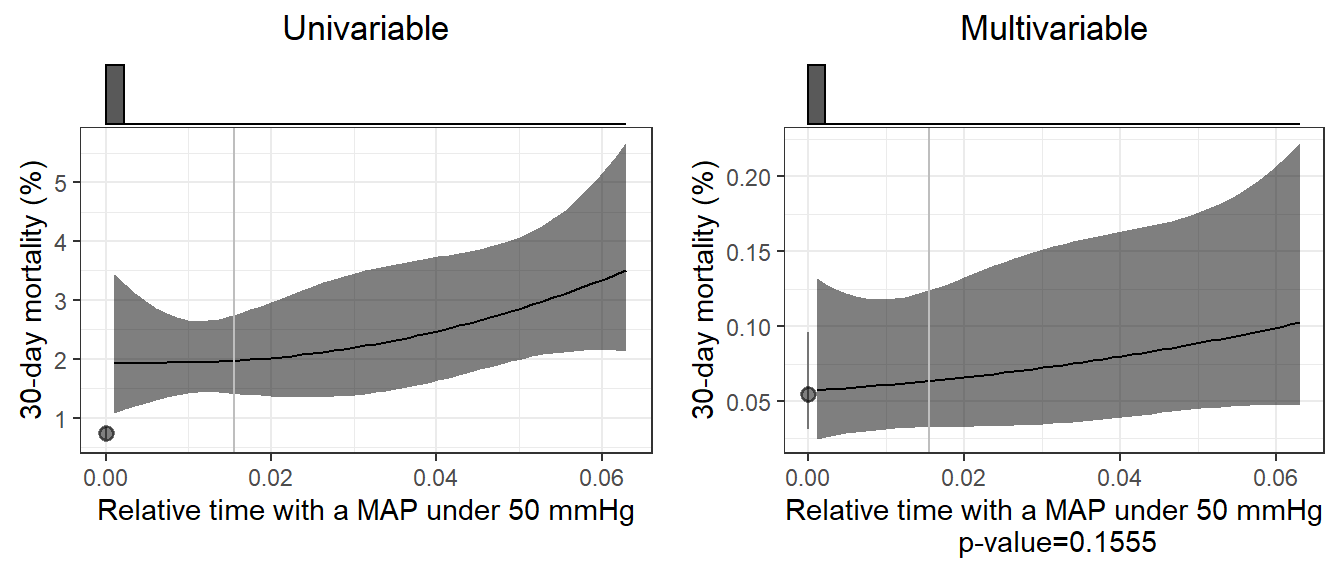

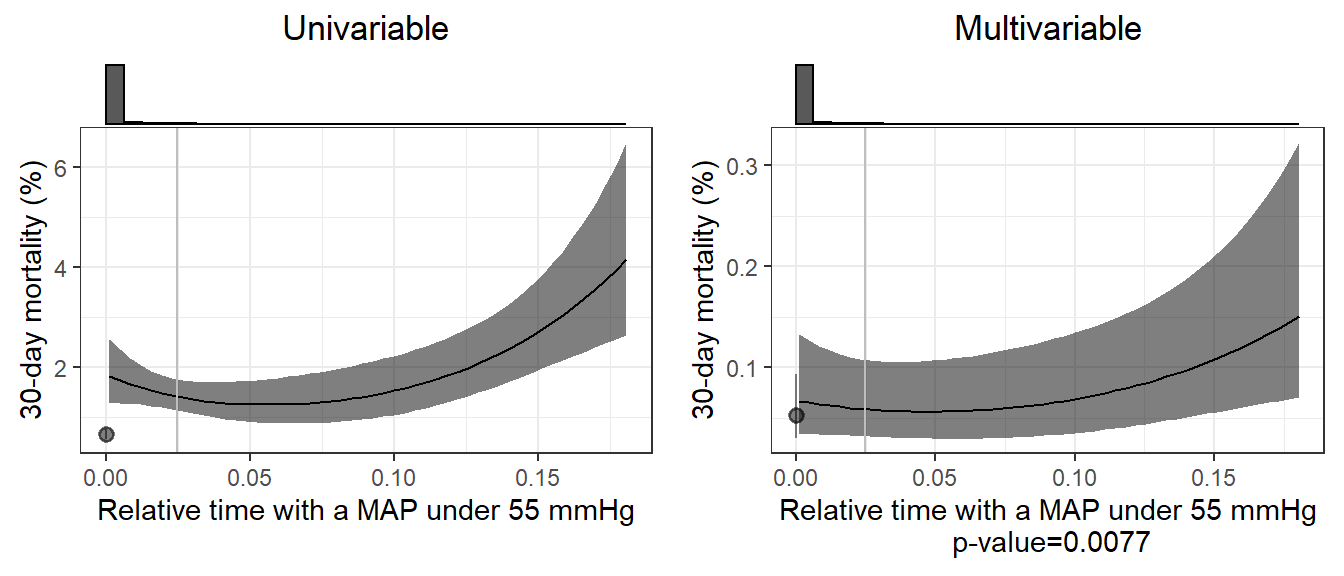

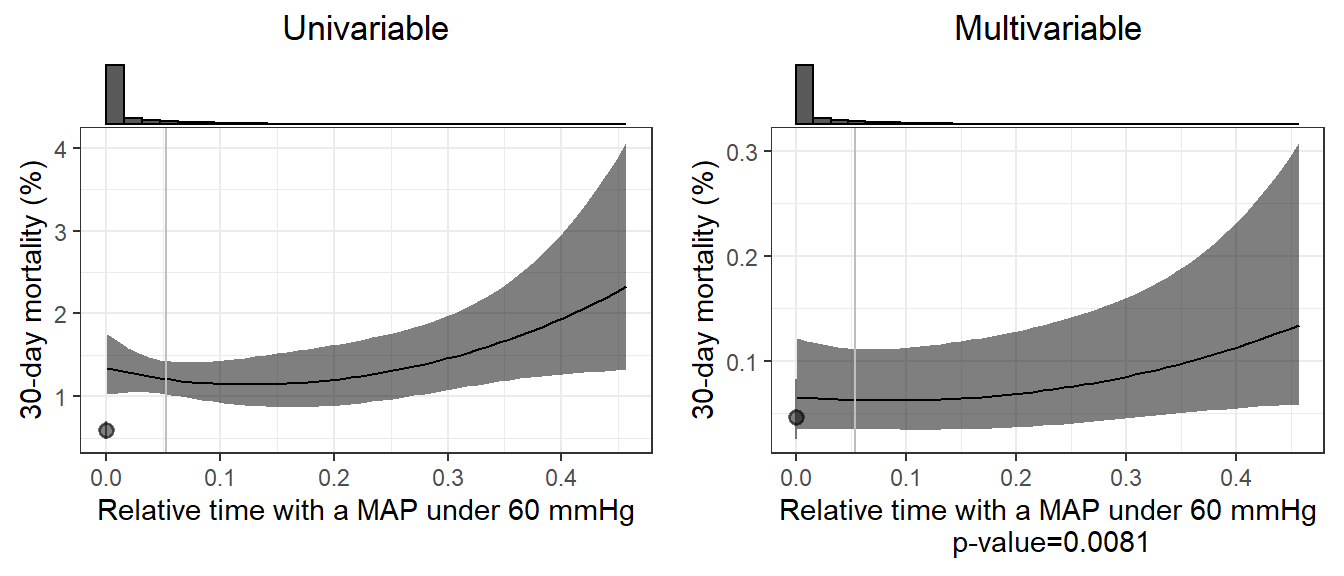

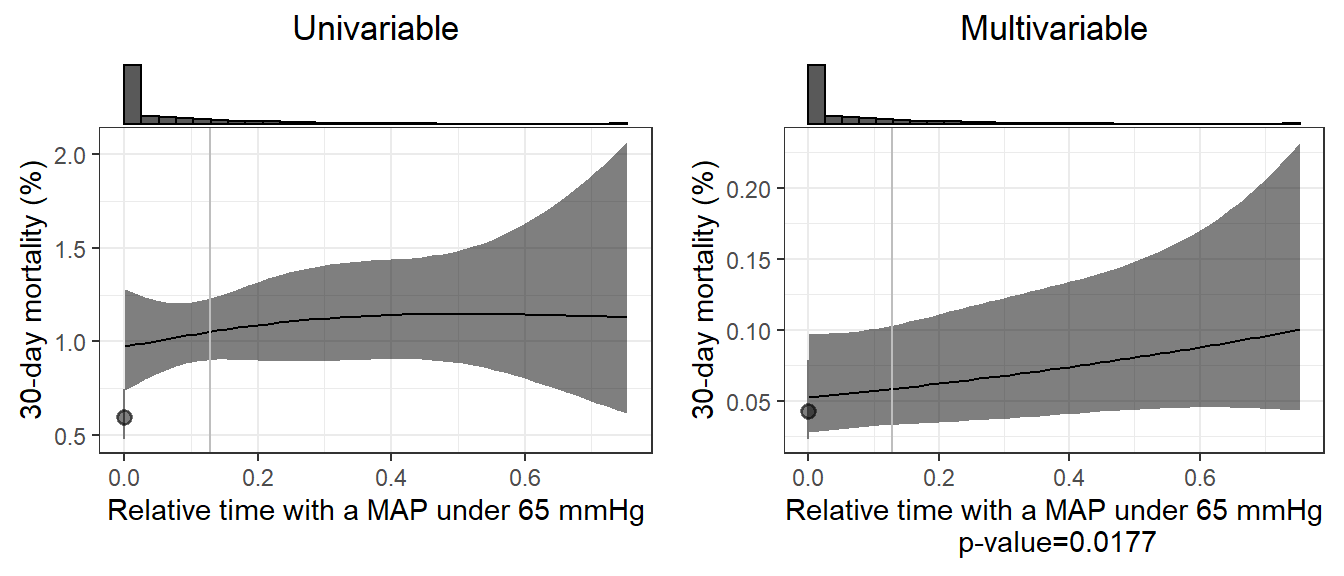

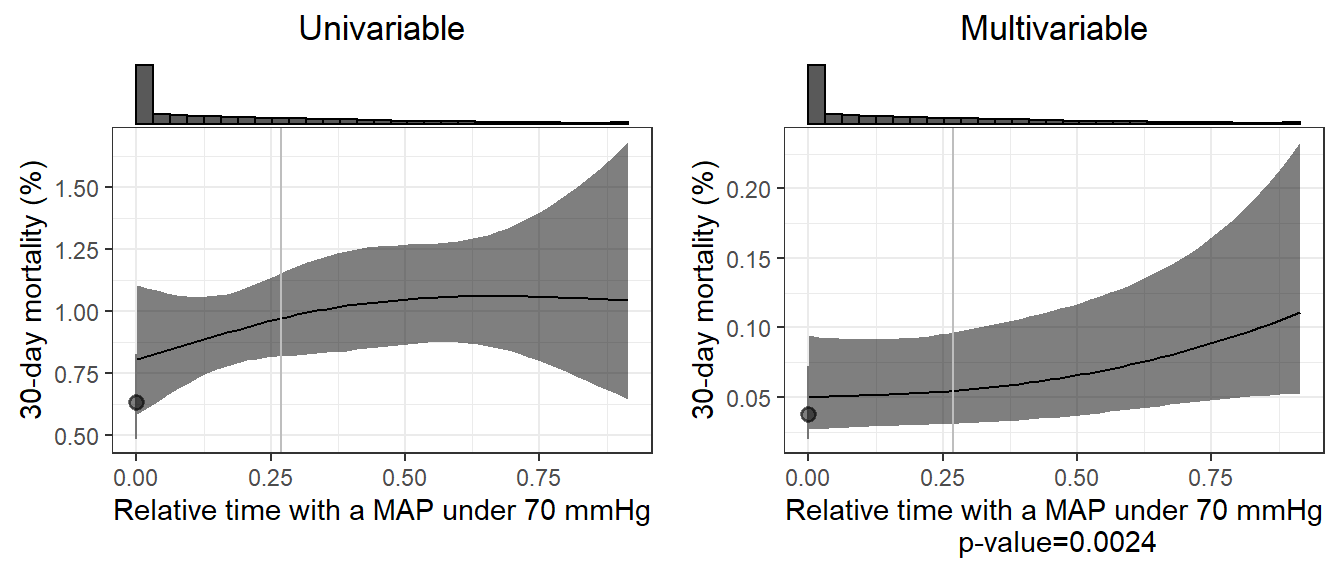

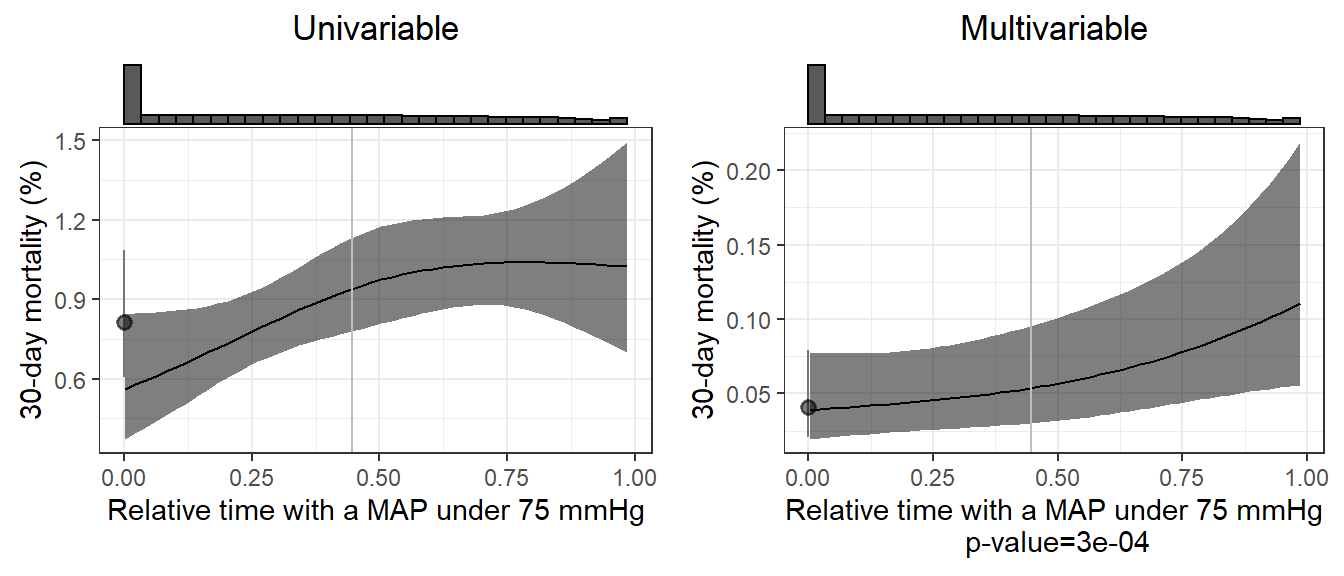

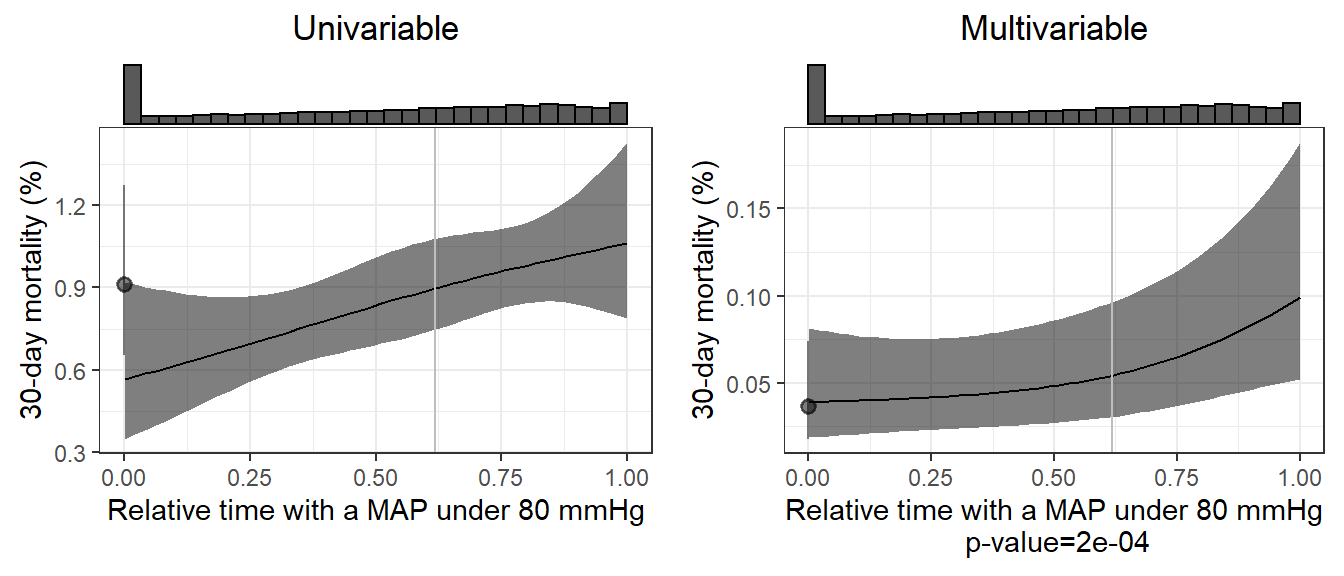


**Brier score of all characterizations:**


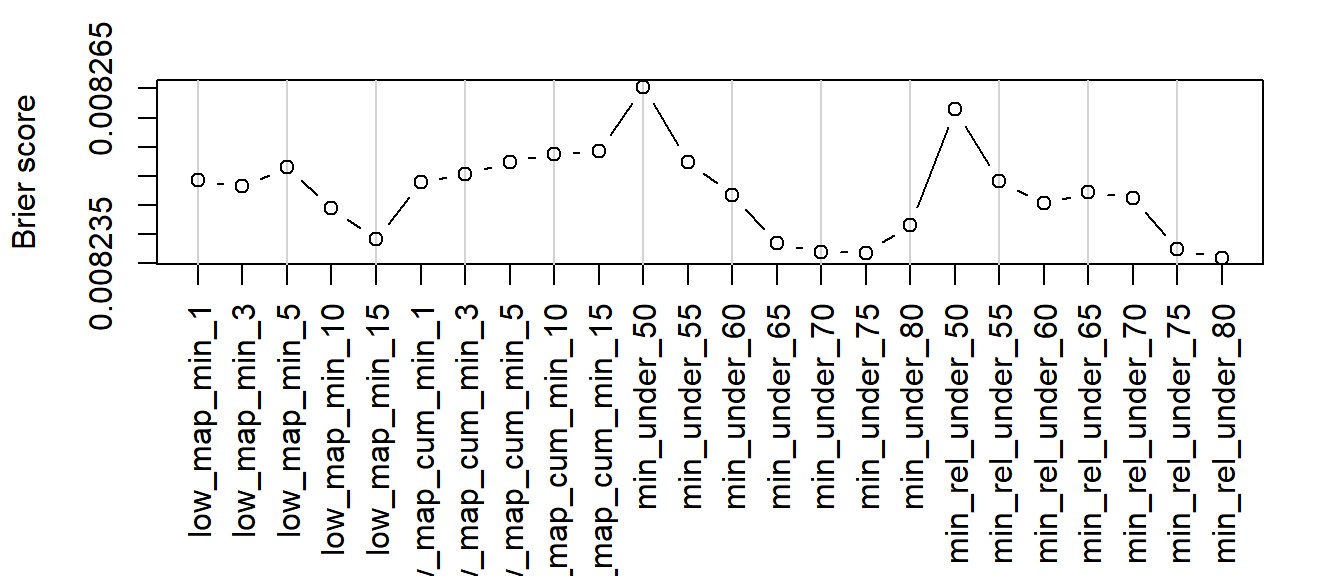
The characterization Relative time with a MAP under 80 shows the best fit in terms of the Brier score.

**Multivariate model’s plot**

The selected characterization “Relative time with a MAP under 80” is tested in the shaping dataset. Results are shown below. Grey areas are 95% Confidence intervals.


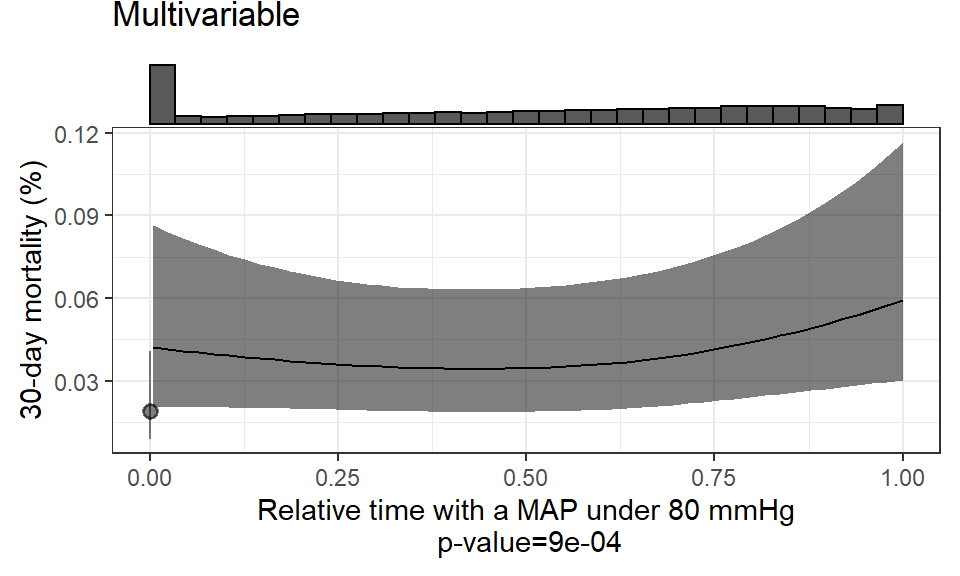


**Brier scores of the covariates**

Below, Brier scores of covariates from the modell using “Relative time with a MAP under 80**”** in the Shaping dataset are shown.Gender_0W: female gender, ns: spline, dauerOPh: duration of surgery [h], e_to_surgery: time to surgery, OPgroup: Surgeries speciality, ASA_Status: ASA status, hypo: IOH characterization.


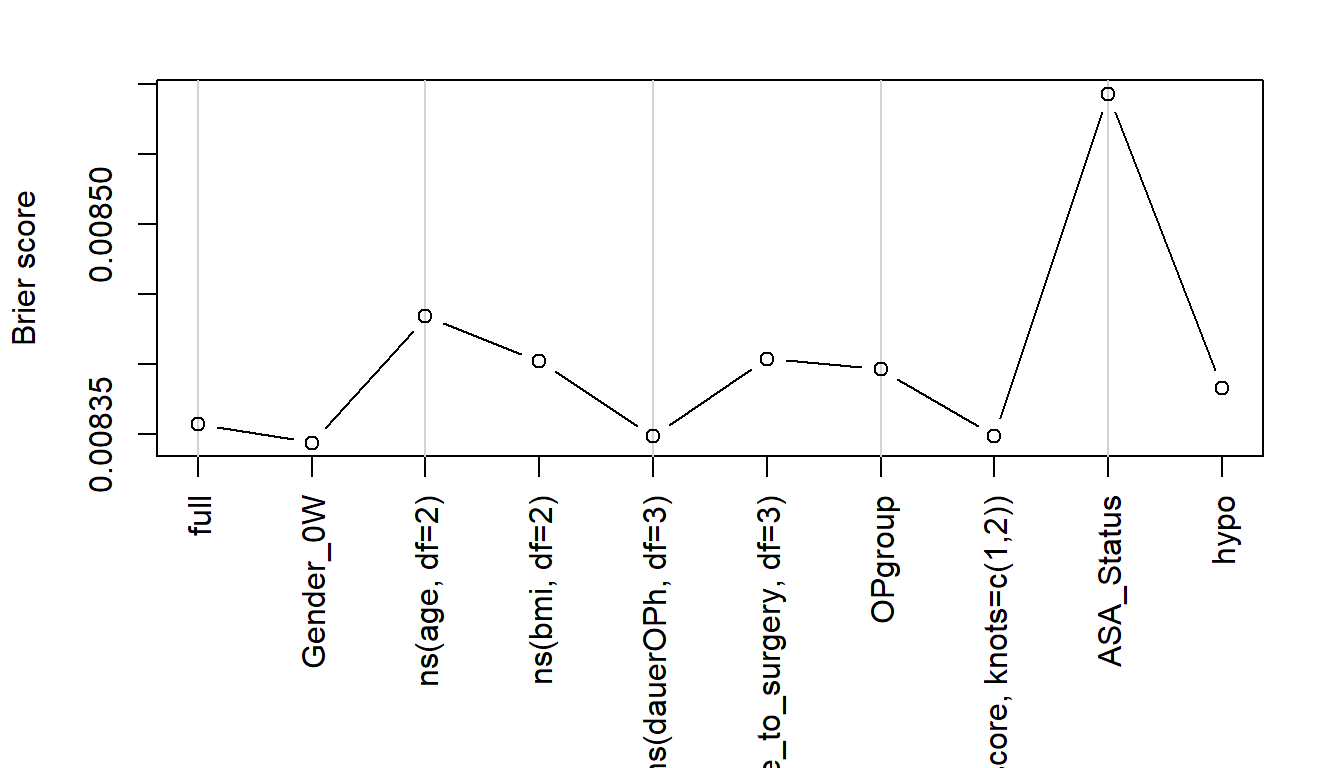


## Hospital - LOS

Plots of uni- and multivariate modells in the shaping dataset:
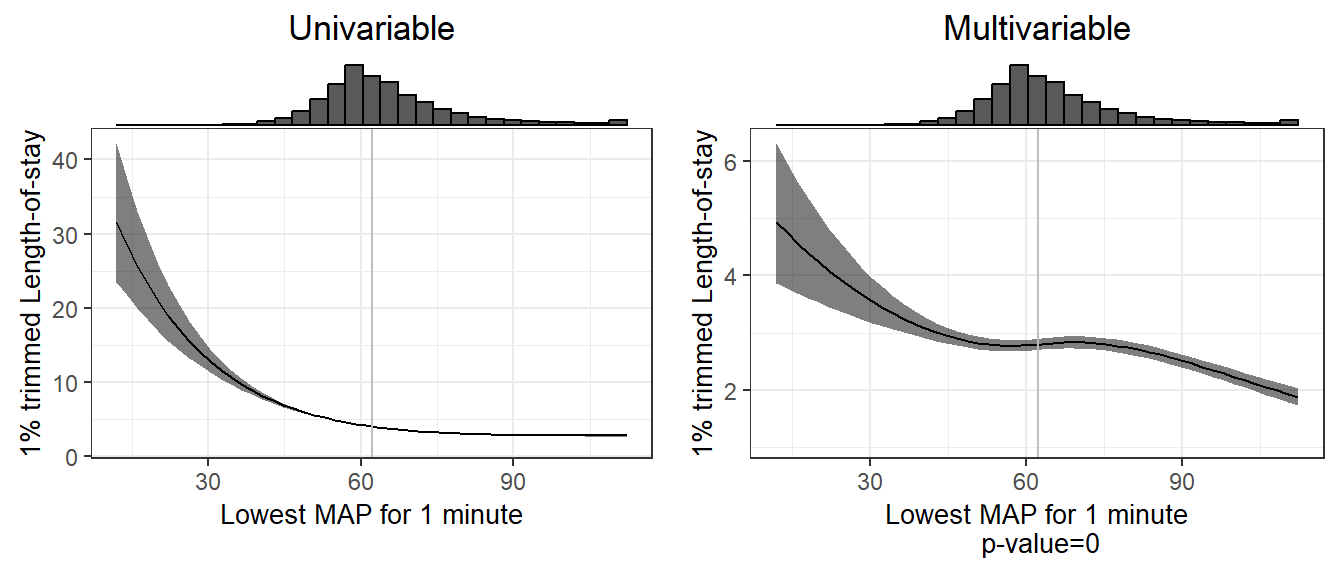

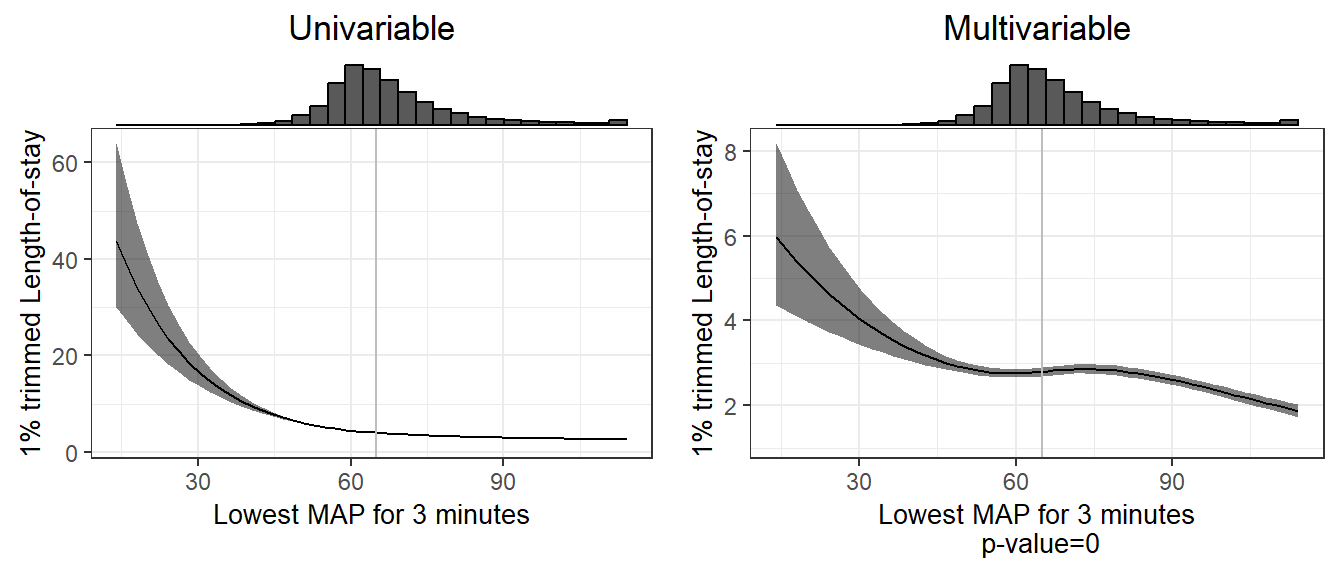

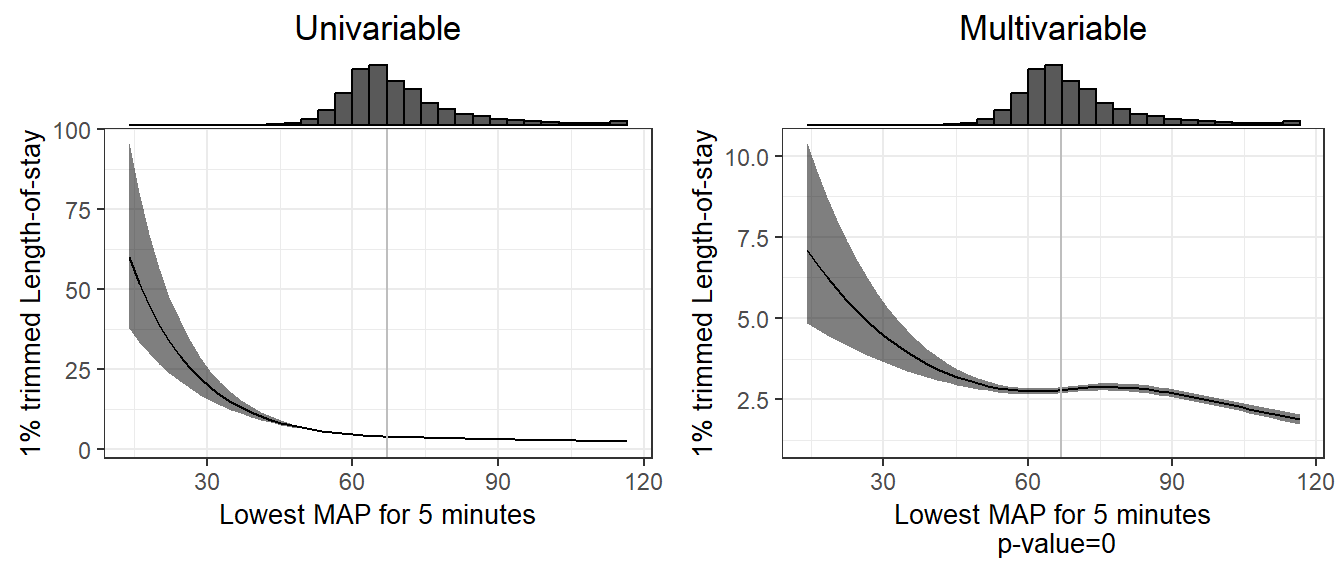

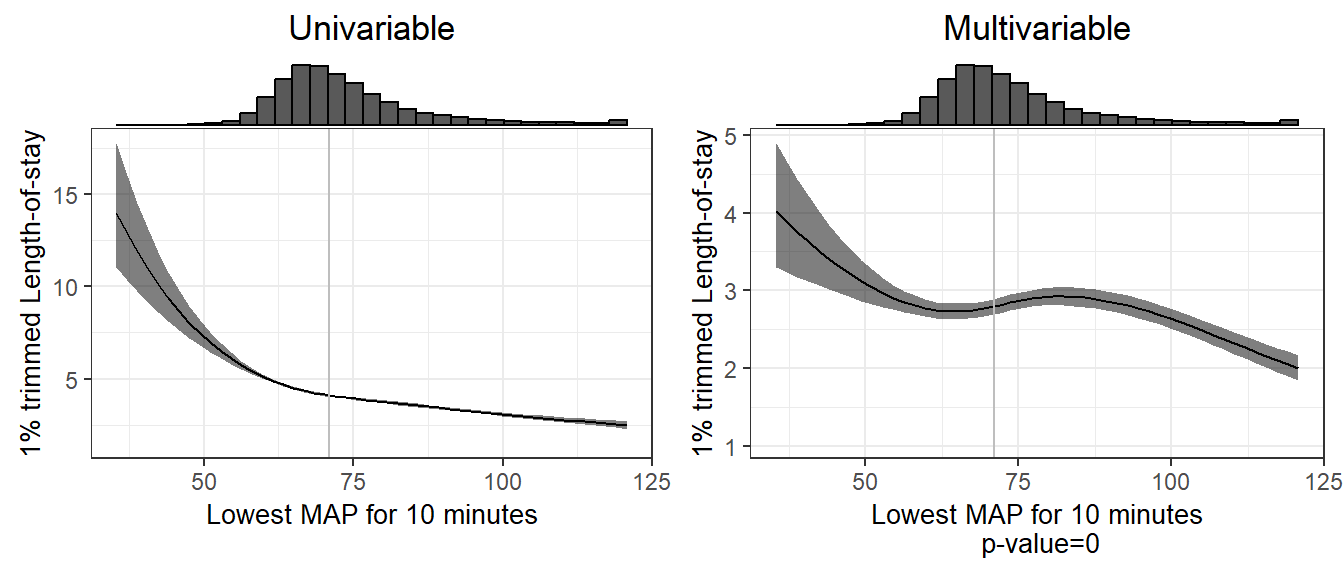

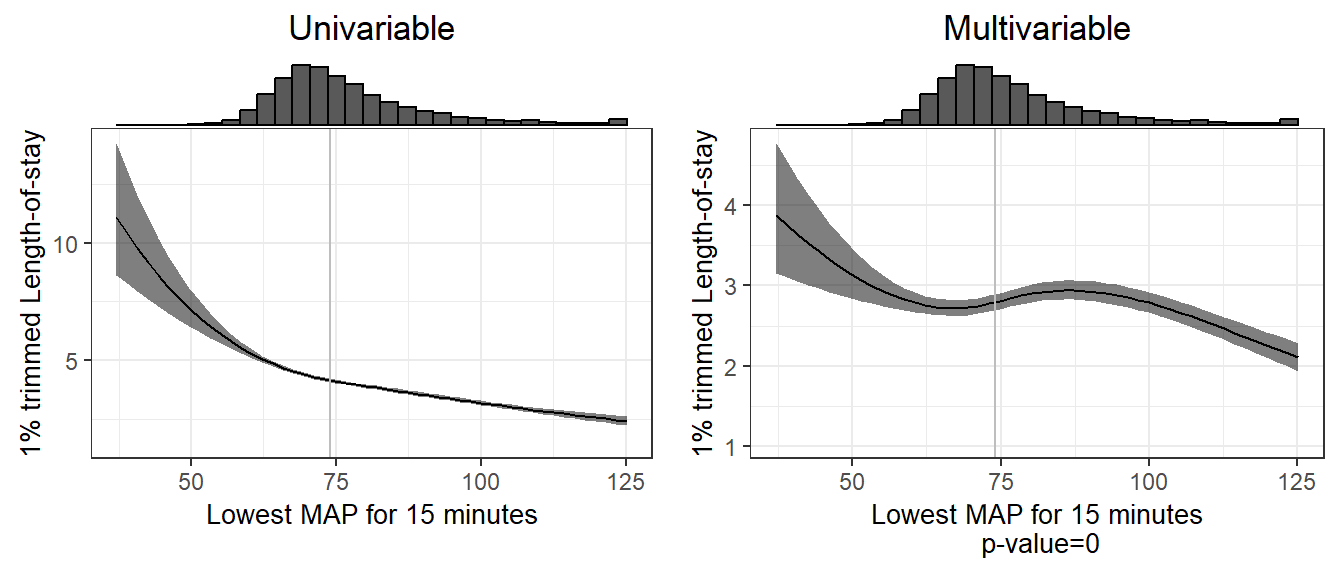

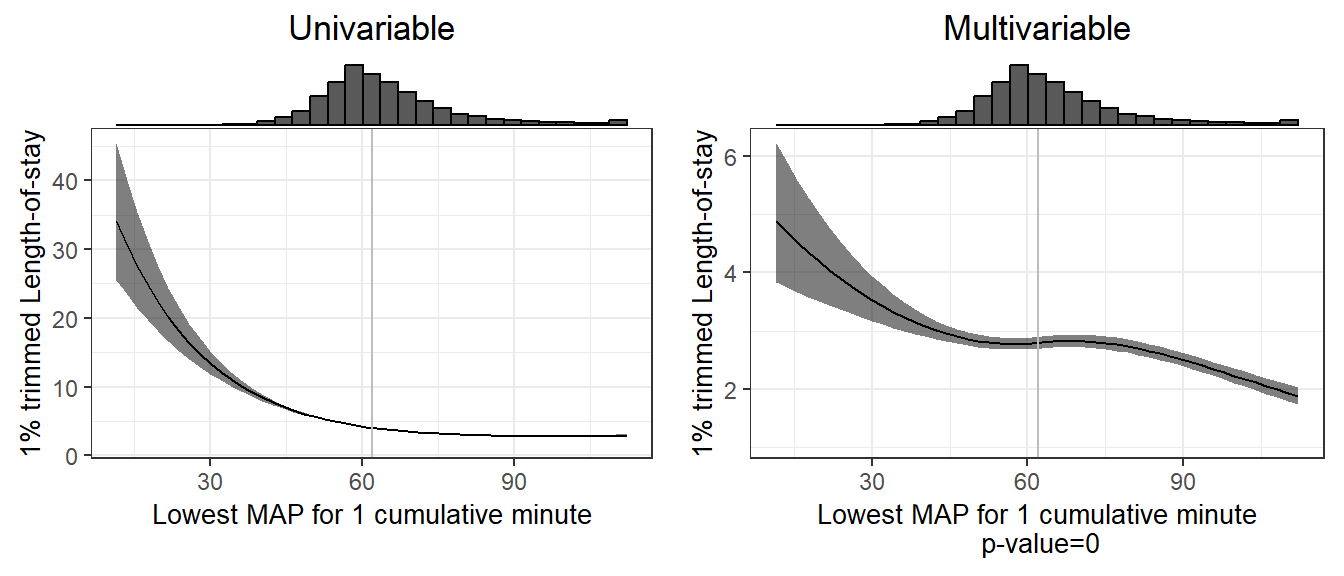

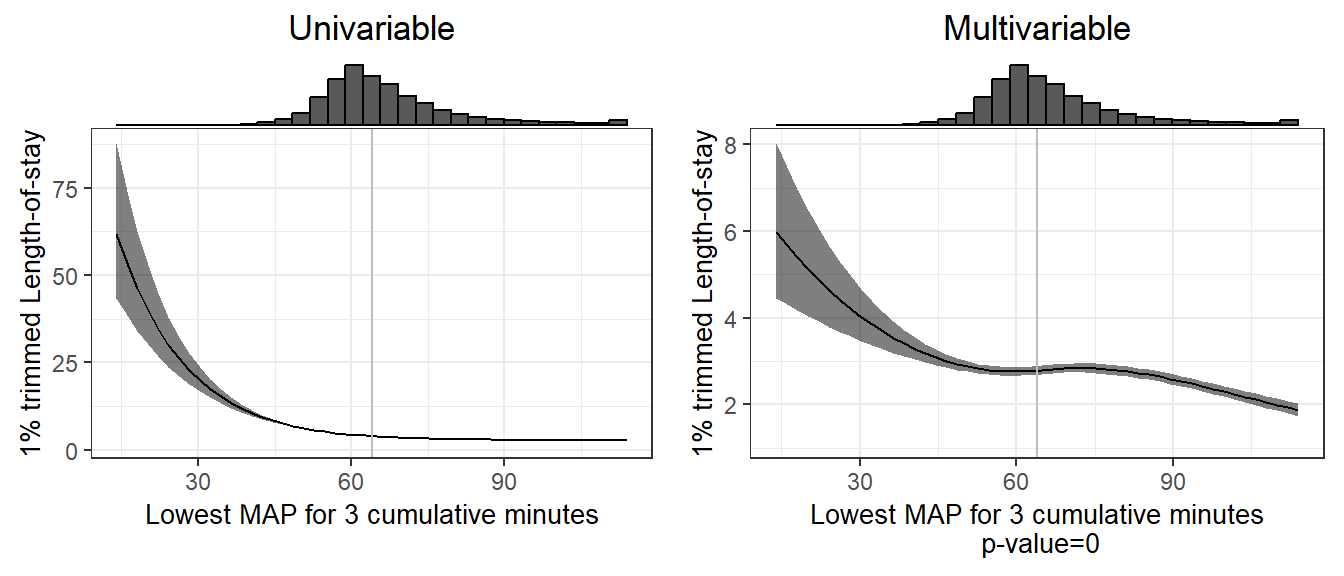

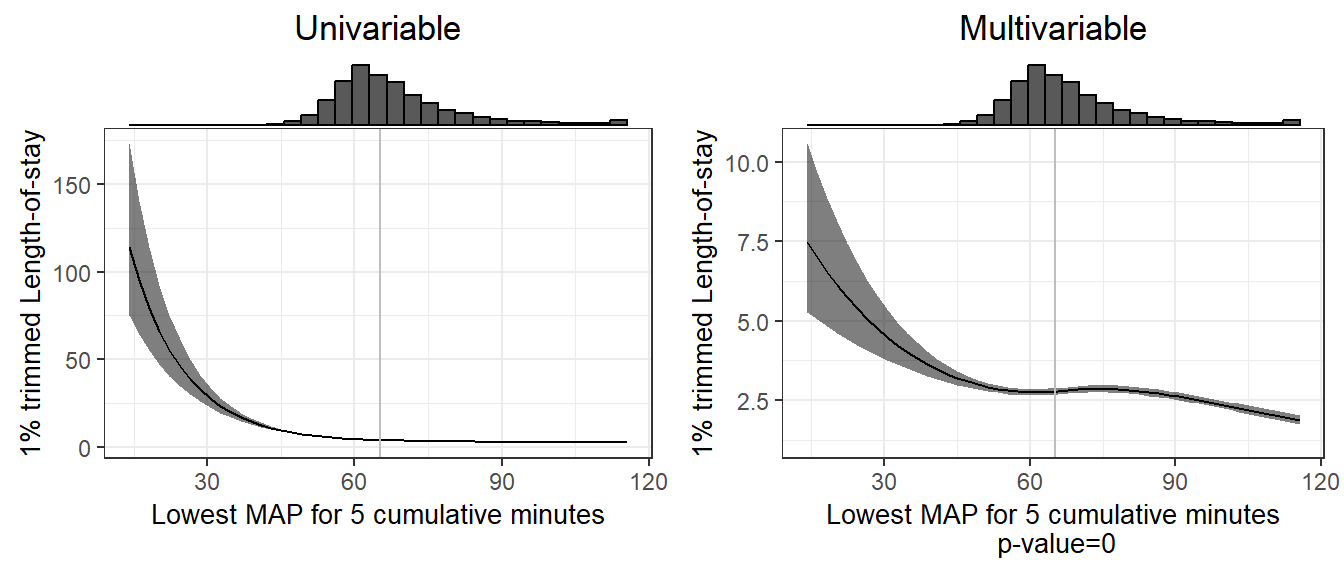

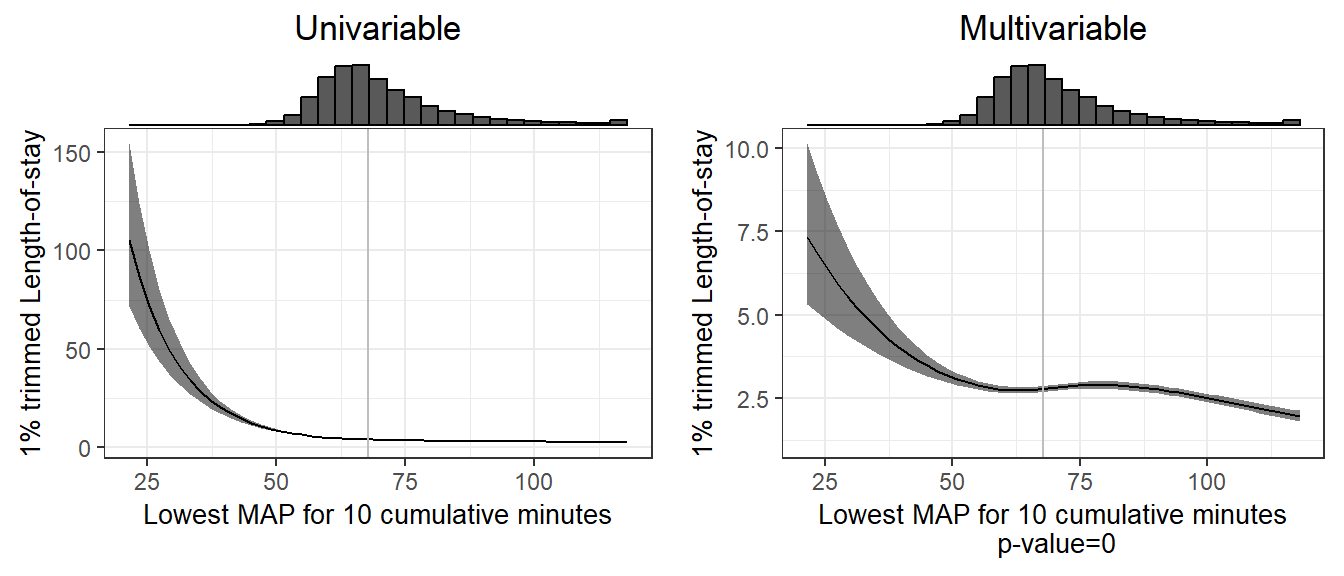

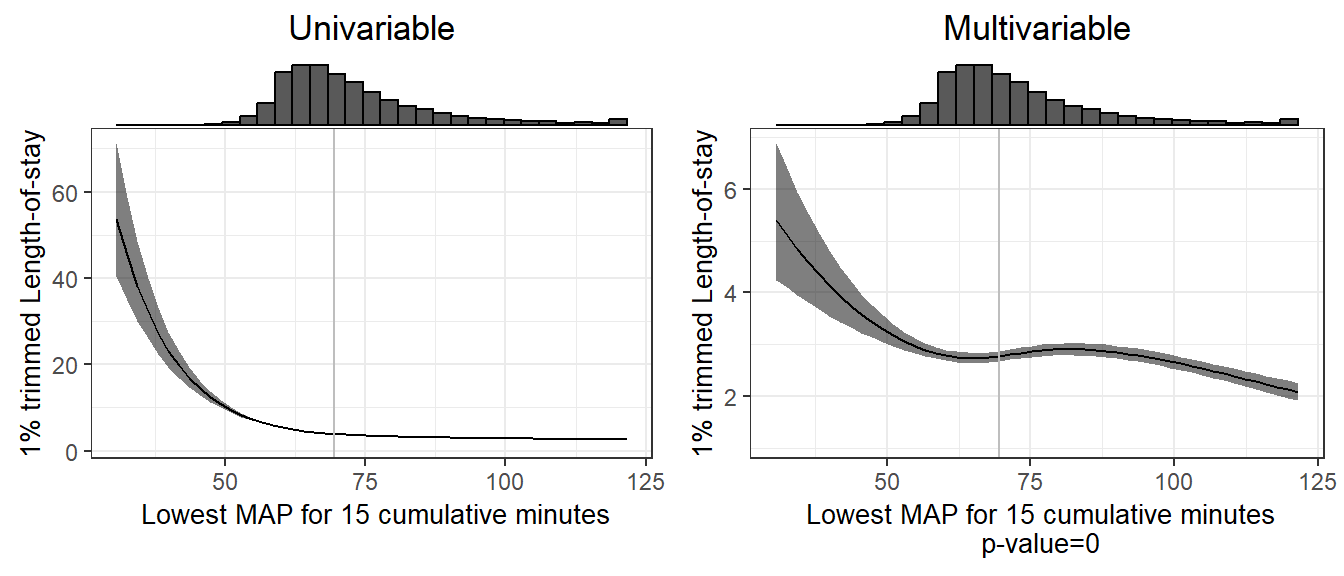

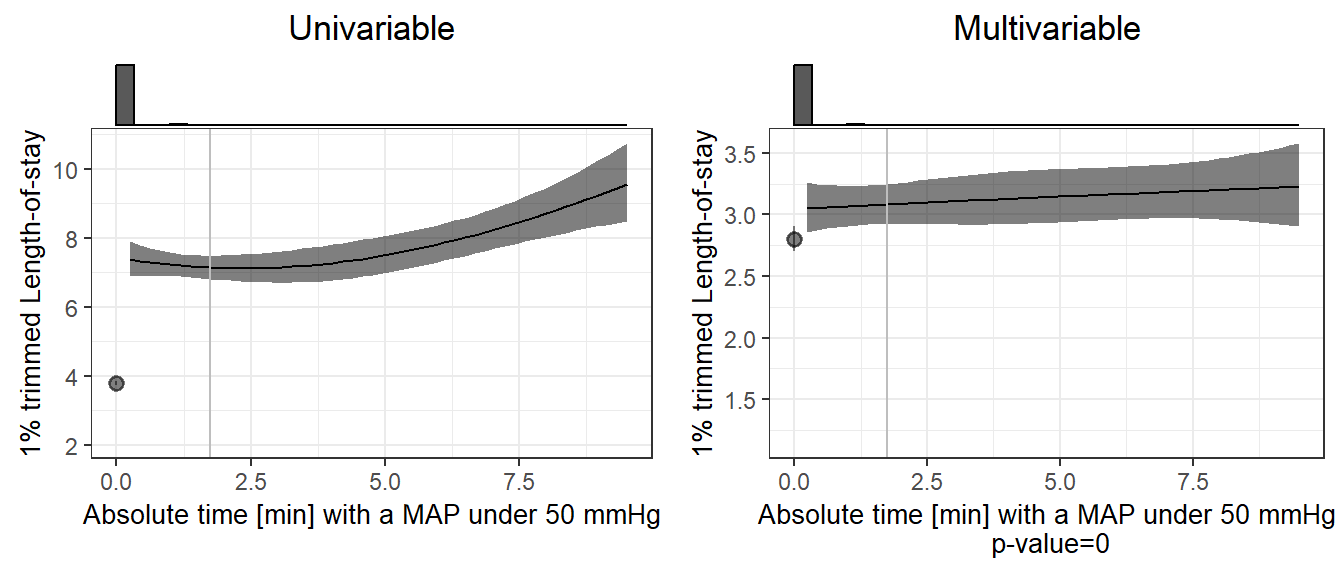

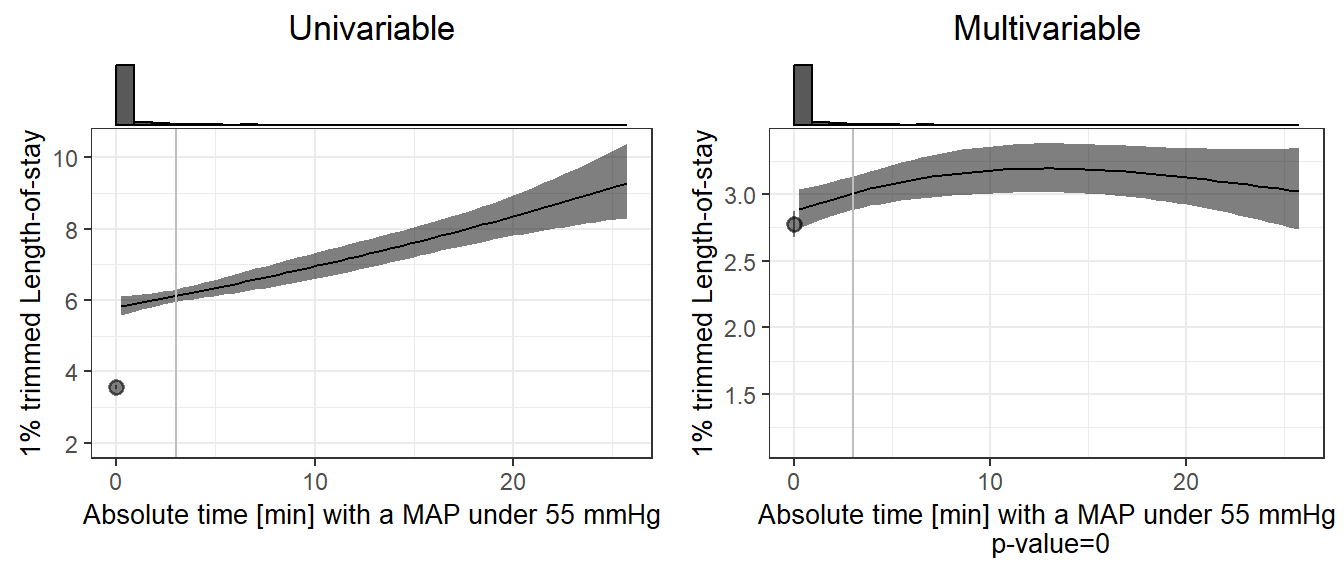

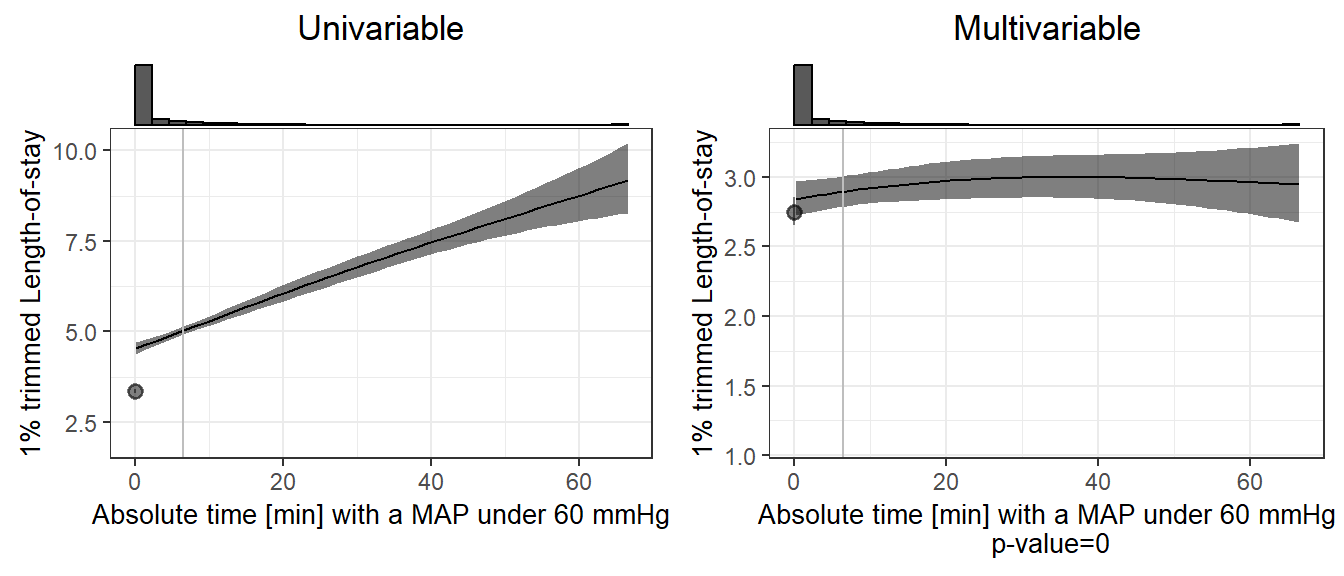

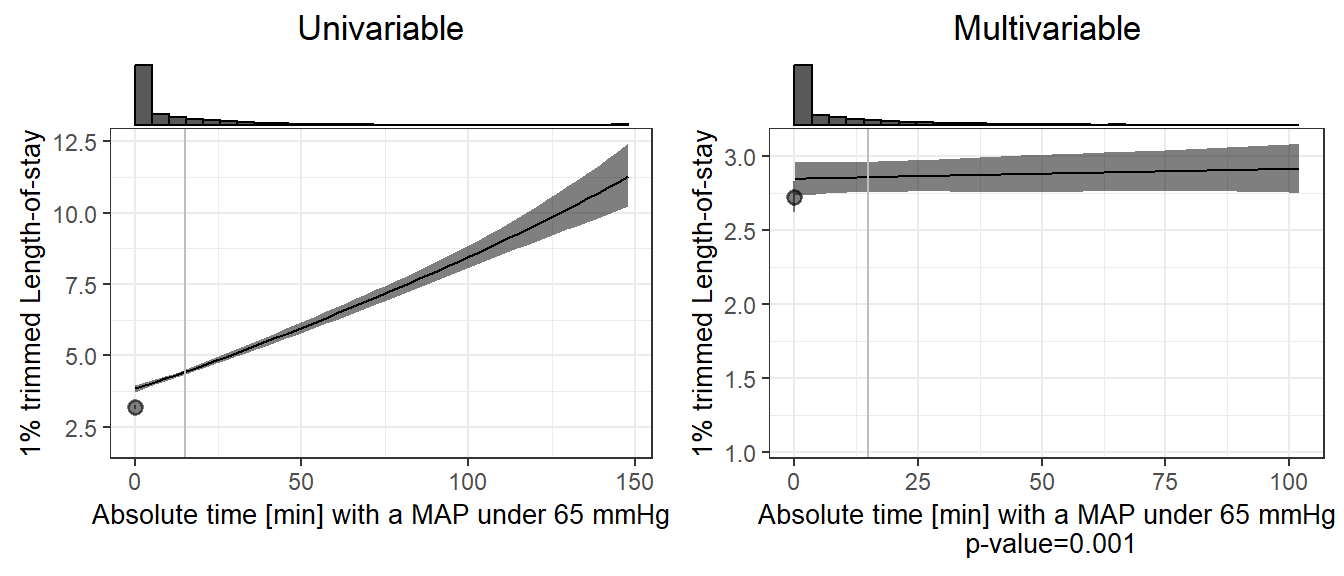

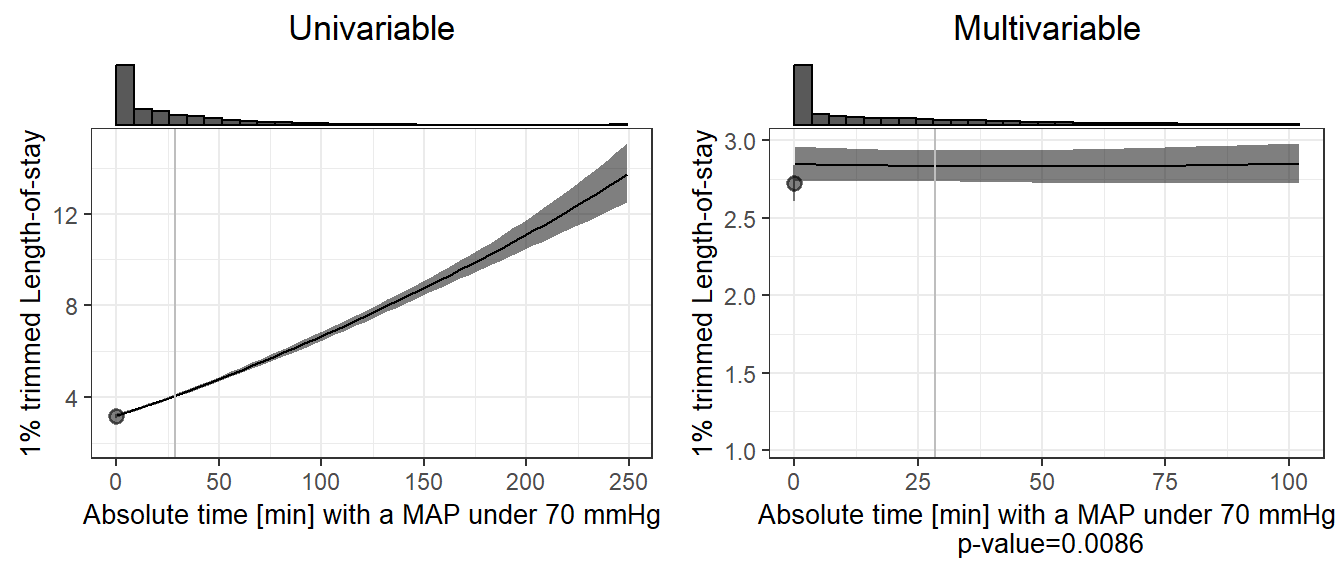

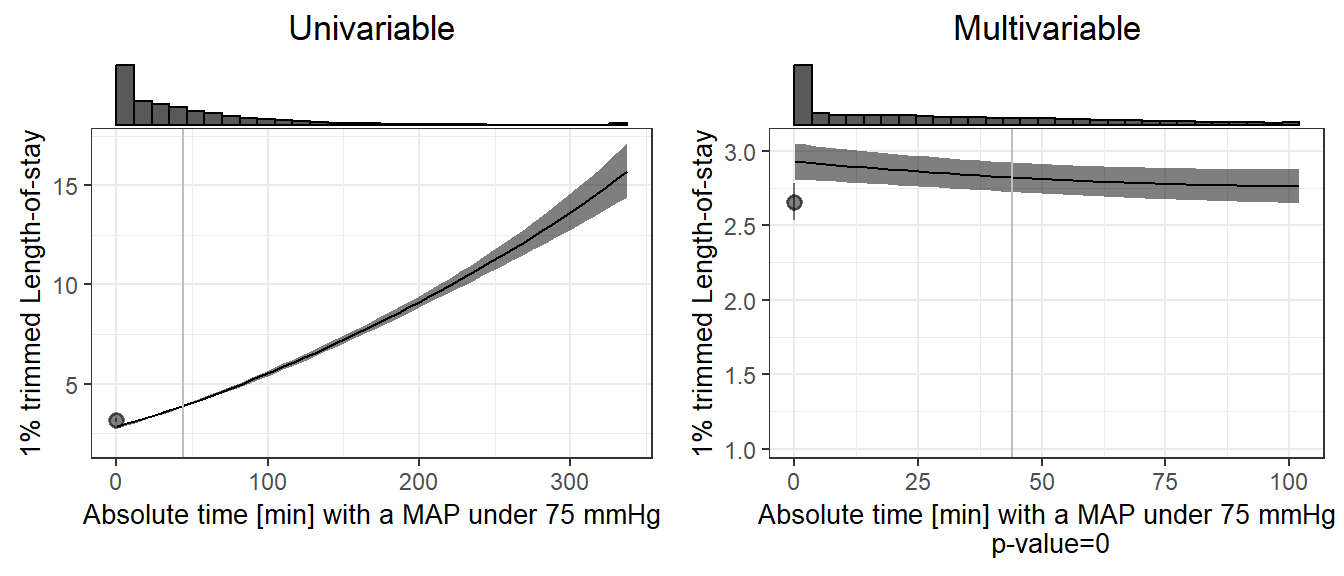

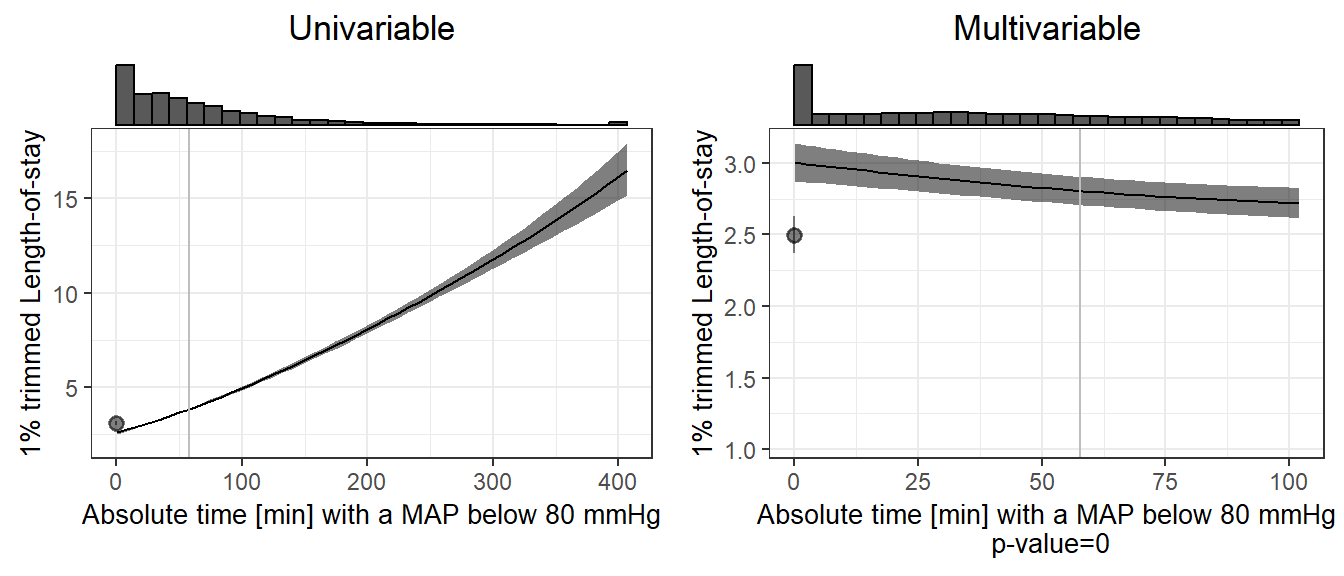

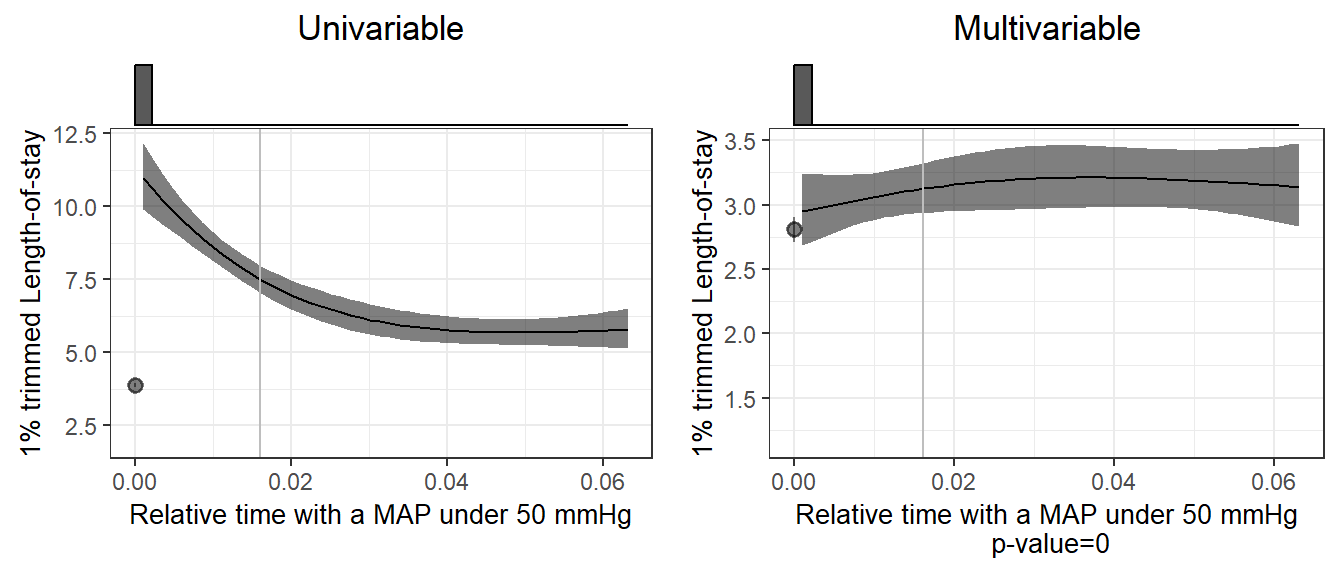

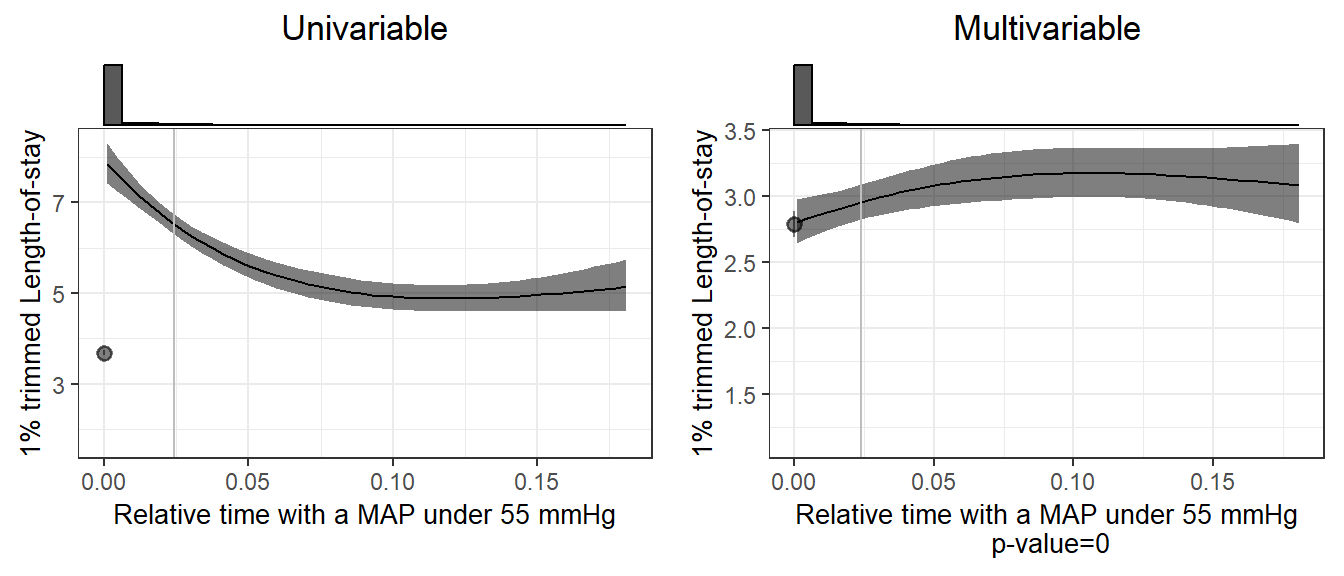

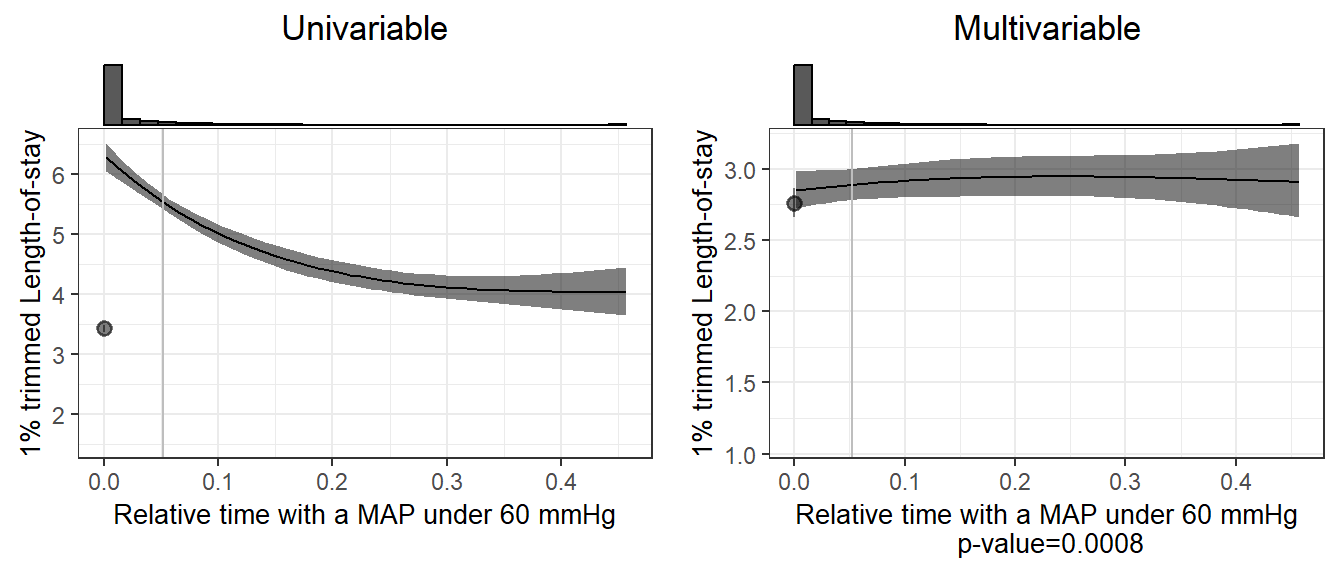

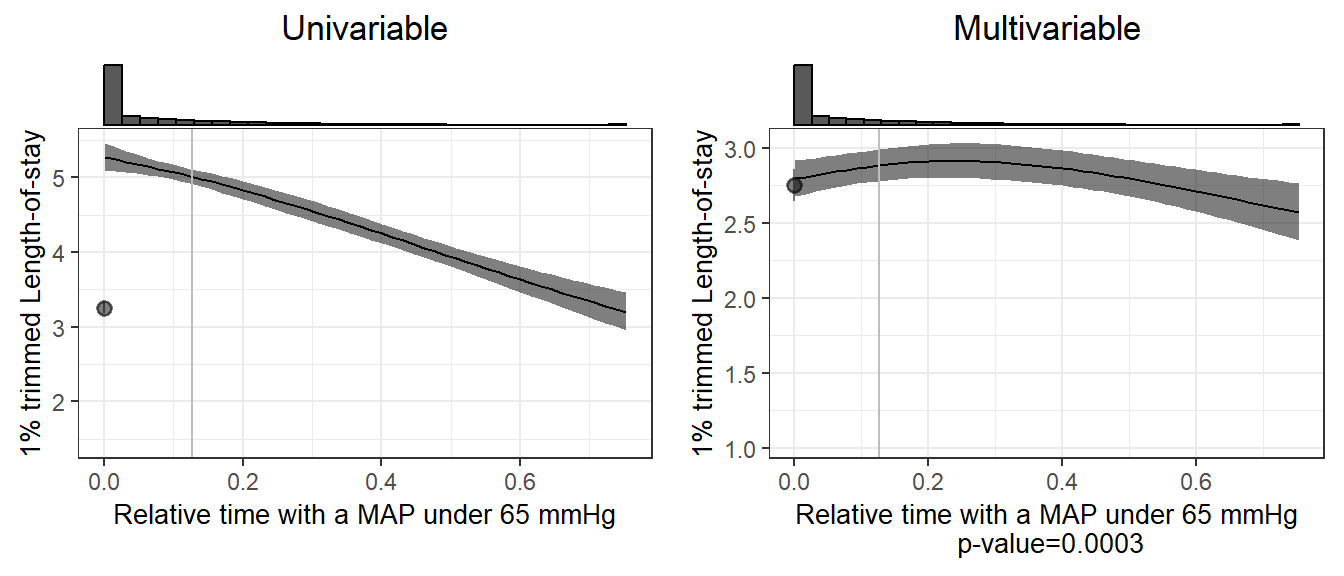

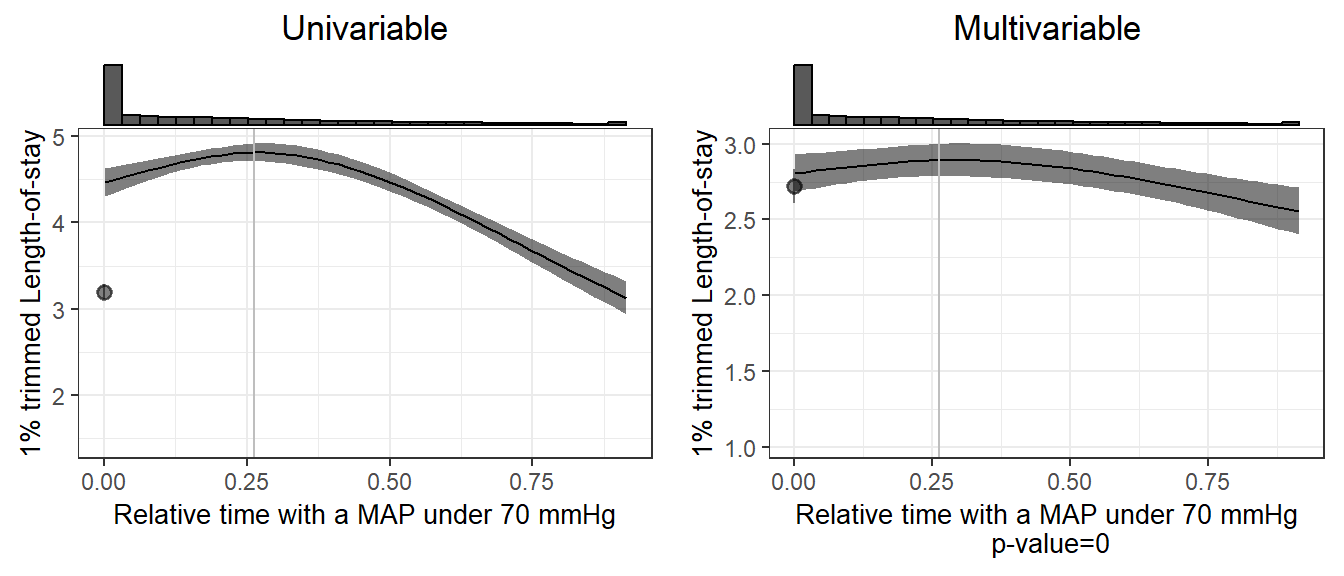

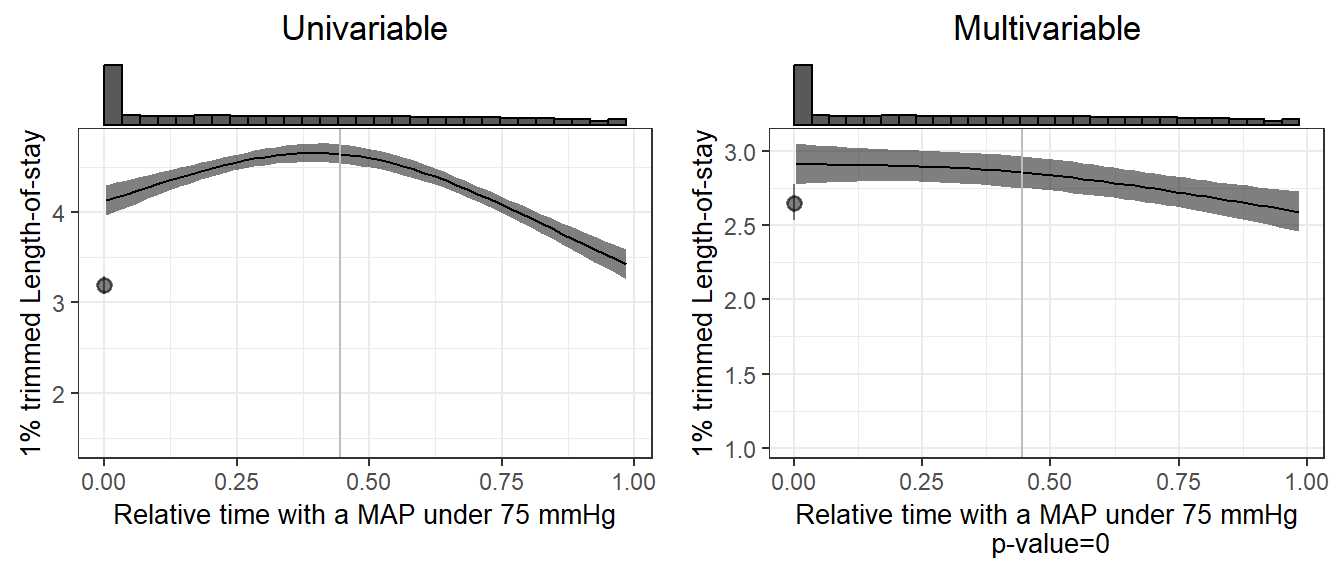

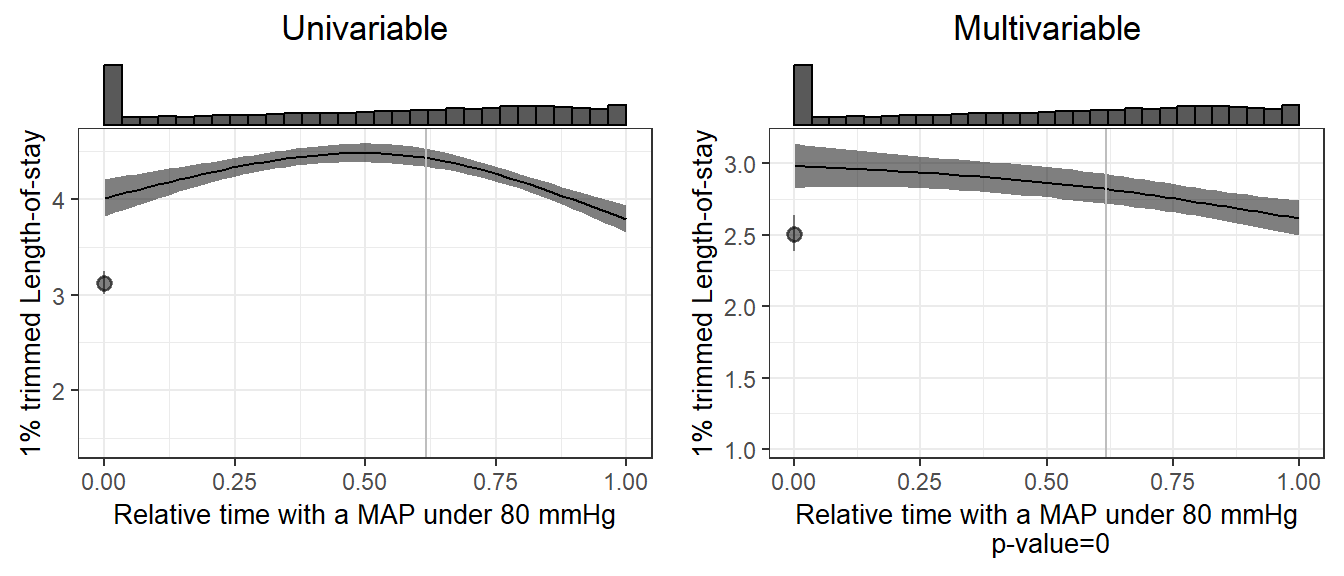


**Mean squared error of all characterizations:**

This figure shows, that low_map_min_1 (Lowest MAP for one minute) ha the least Mean squared error (MSE) and is therefore selected.


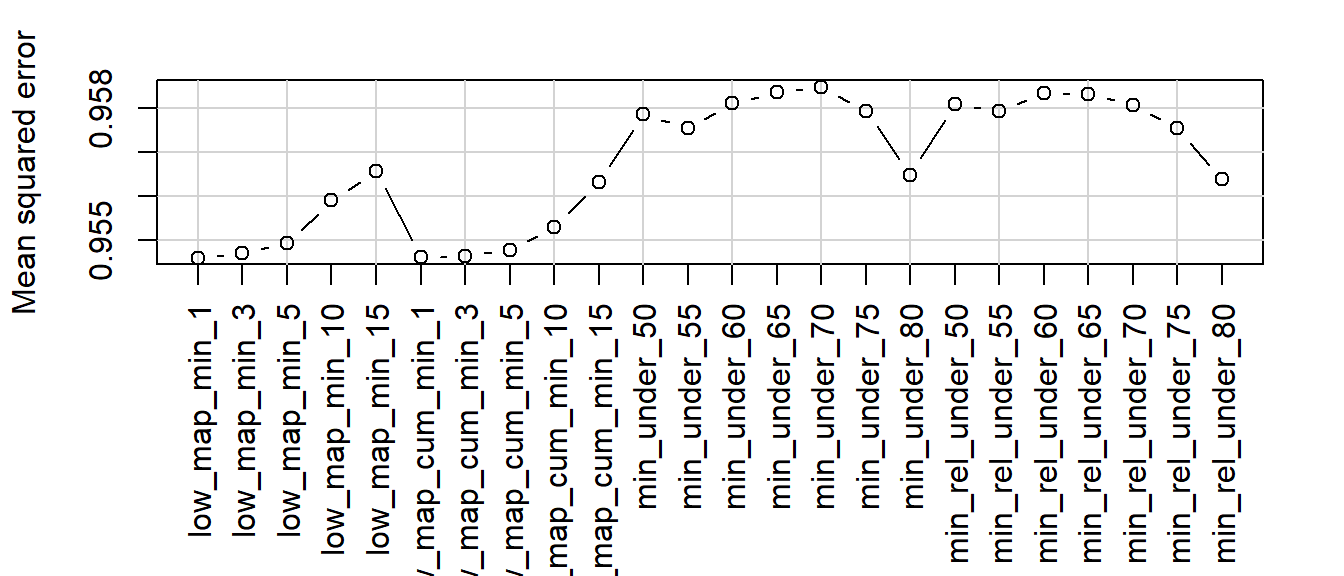


**Multivariate model’s plot (Lowest MAP for one minute) in the shaping dataest:**


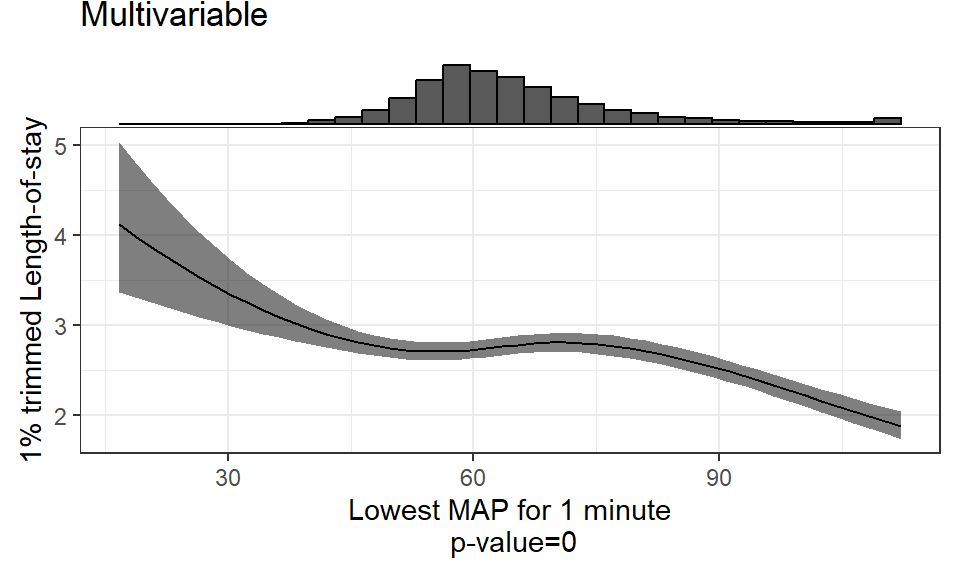


**MSE of the covariates**

Below, MSE of covariates from the modell using “Lowest MAP for one minute**”** in the Shaping dataset are shown.Gender_0W: female gender, ns: spline, dauerOPh: duration of surgery [h], e_to_surgery: time to surgery, OPgroup: Surgeries speciality, ASA_Status: ASA status, hypo: IOH characterization.


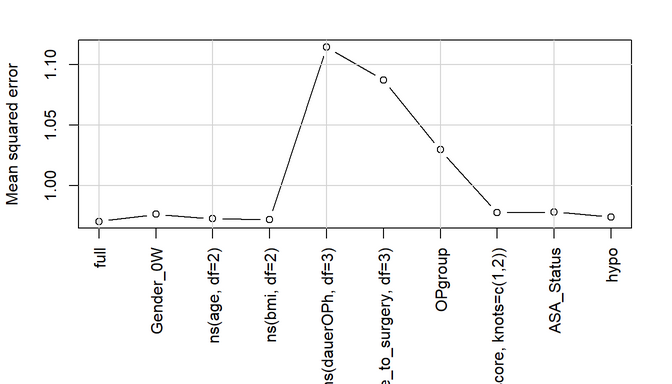


## PACU-LOS

Plots of uni- and multivariate modells in the shaping dataset:
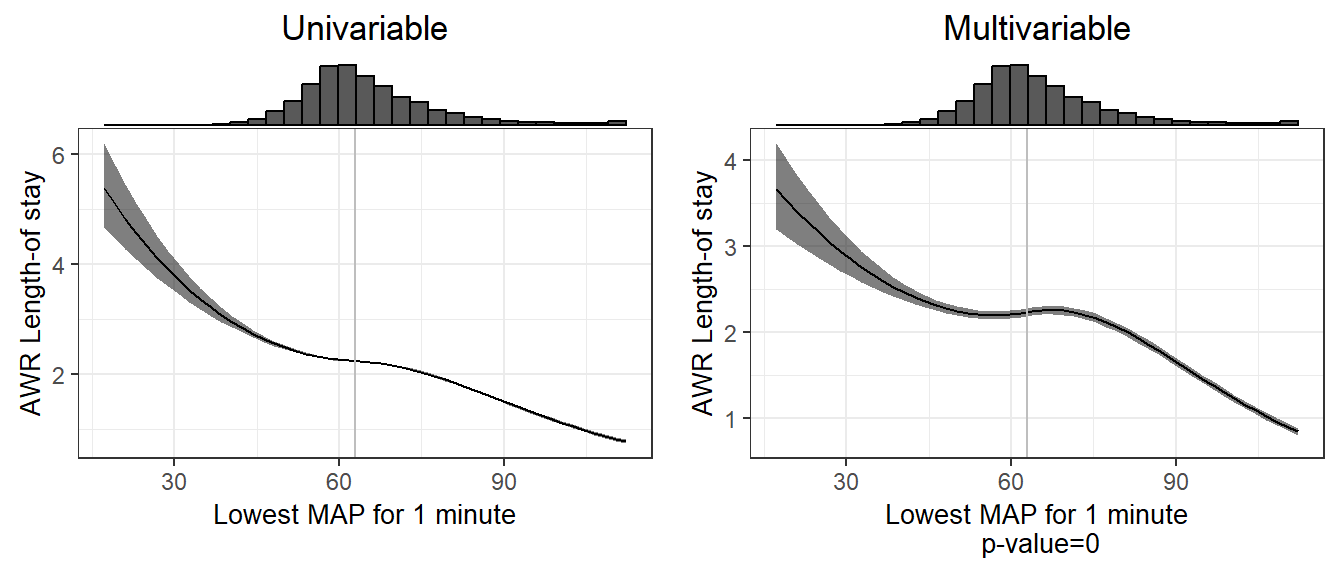

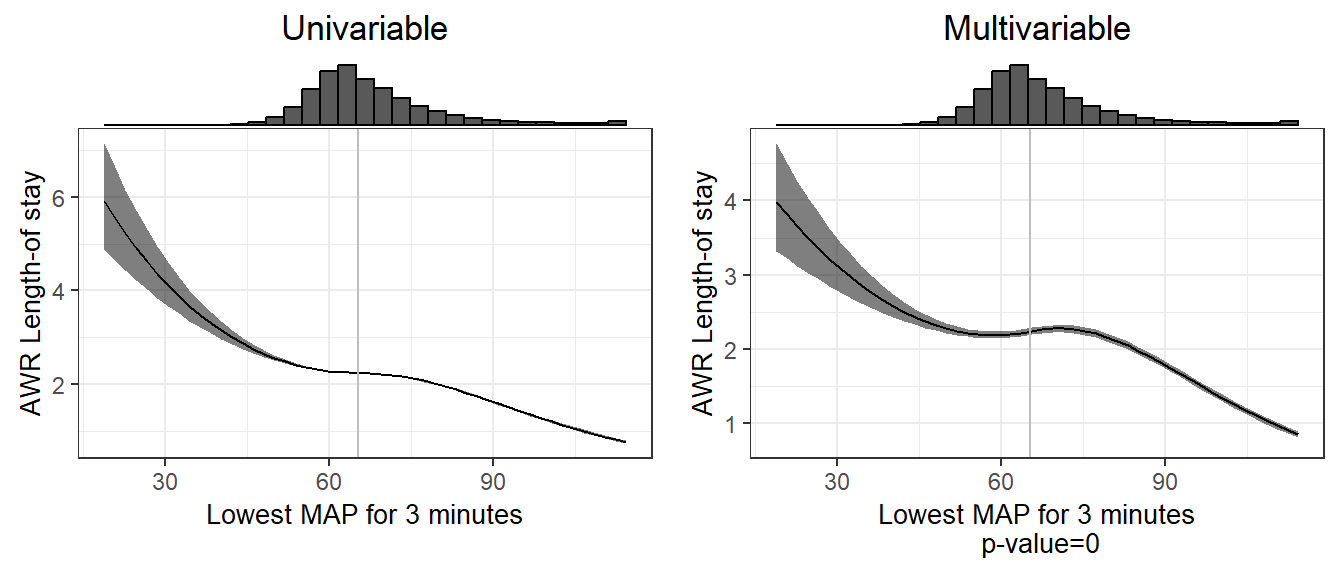

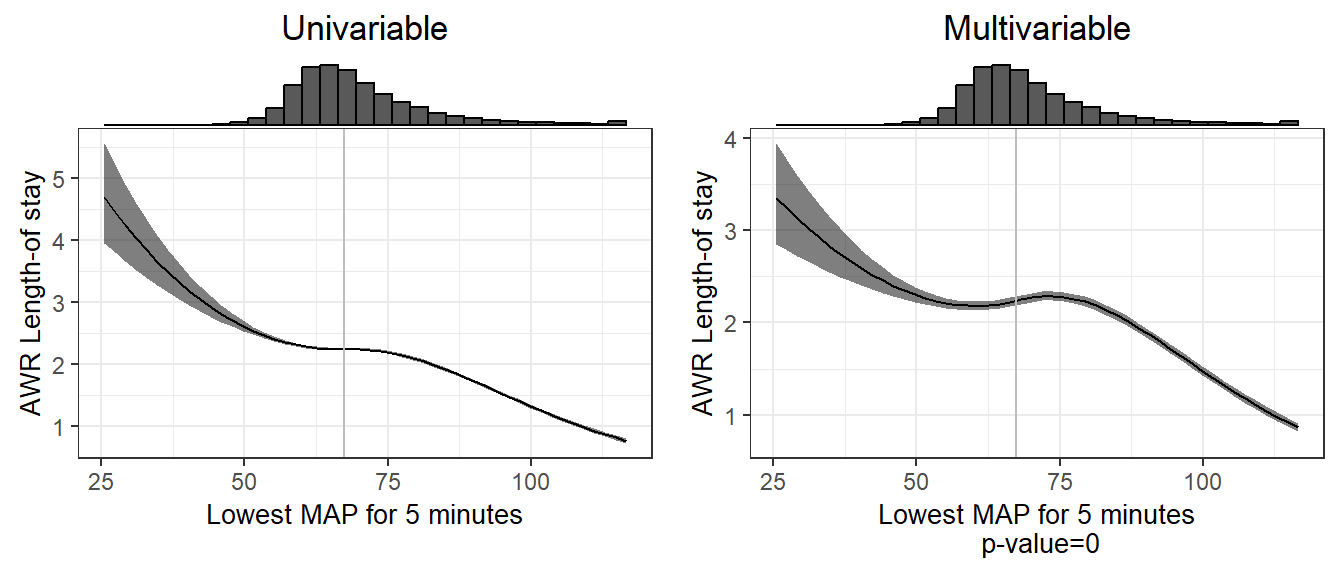

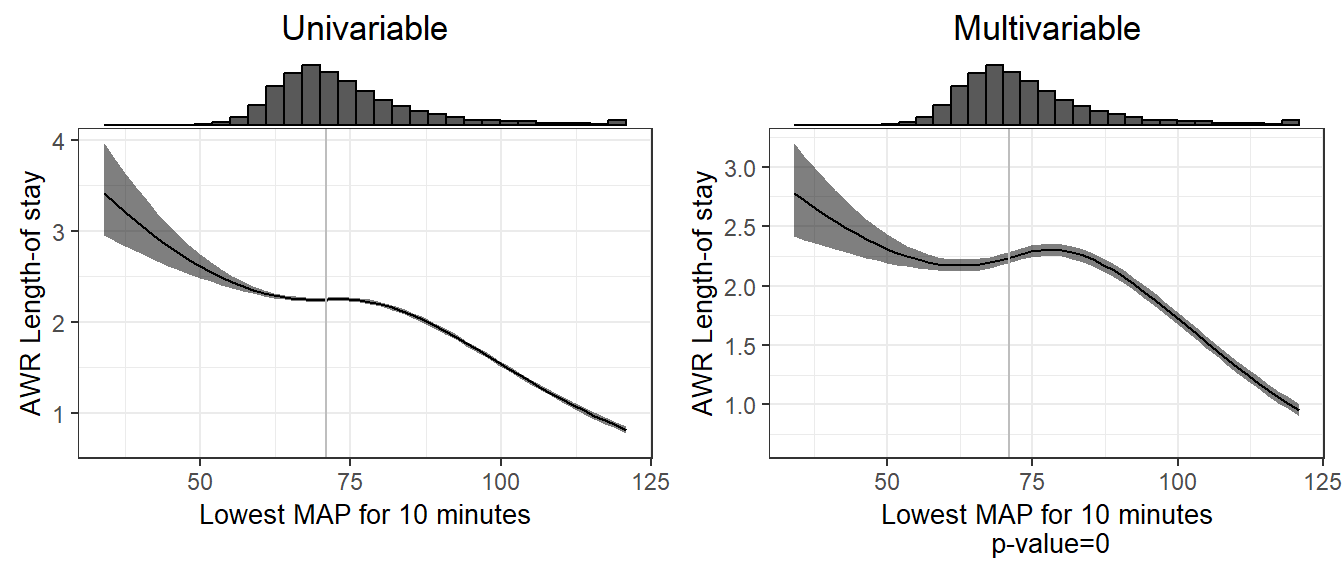

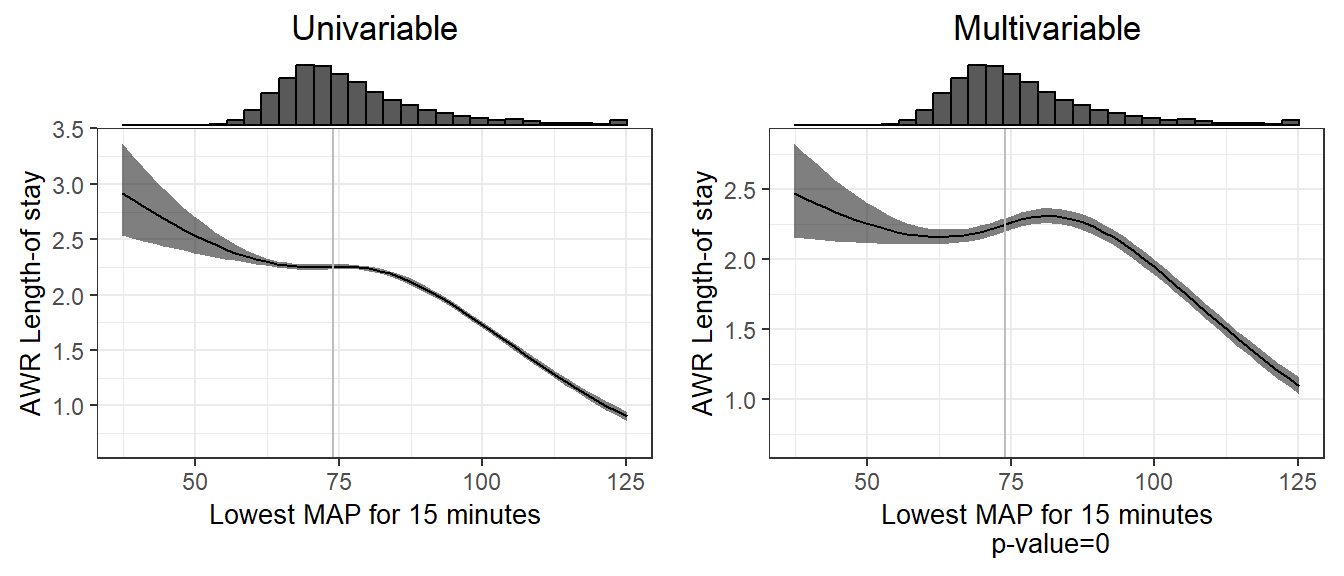

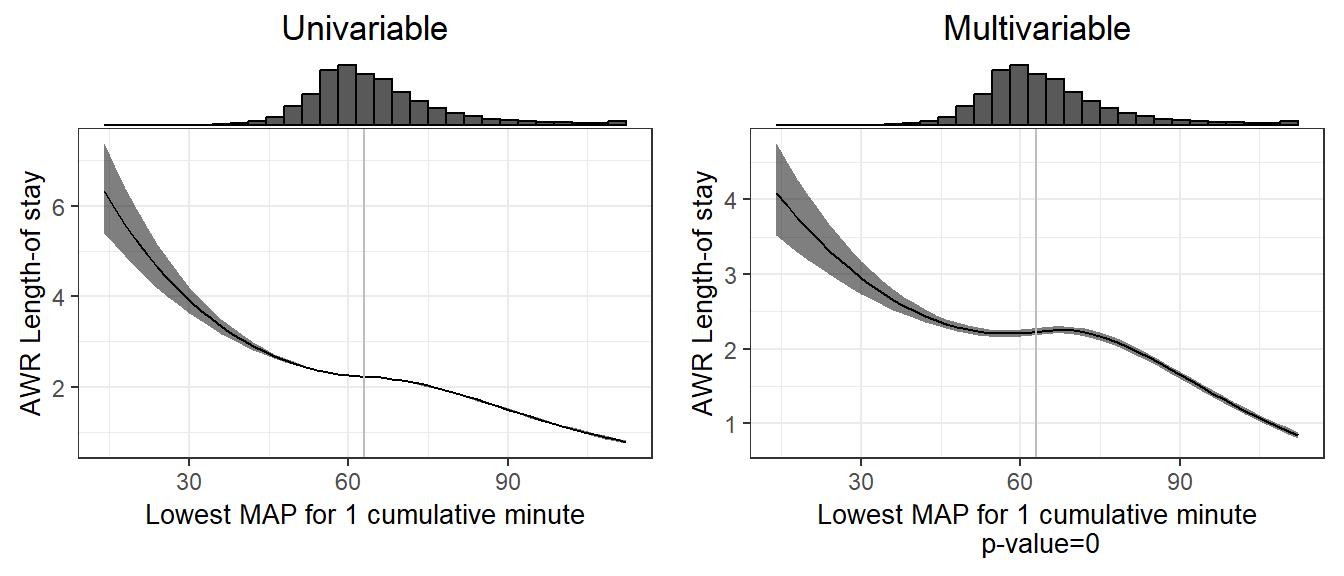

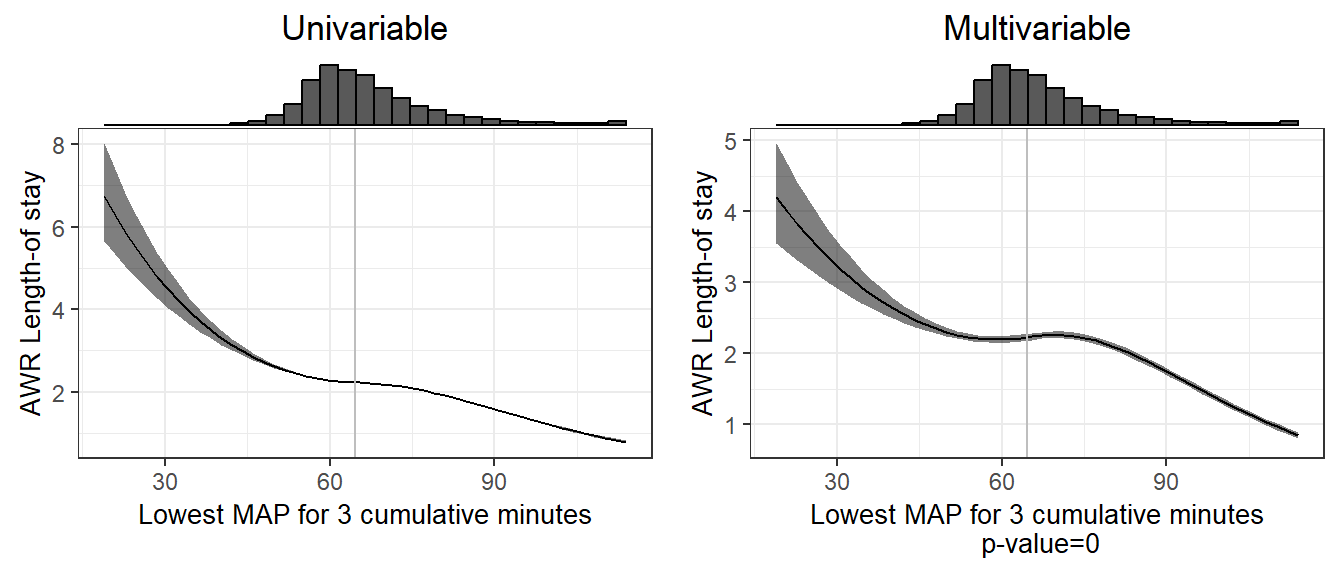

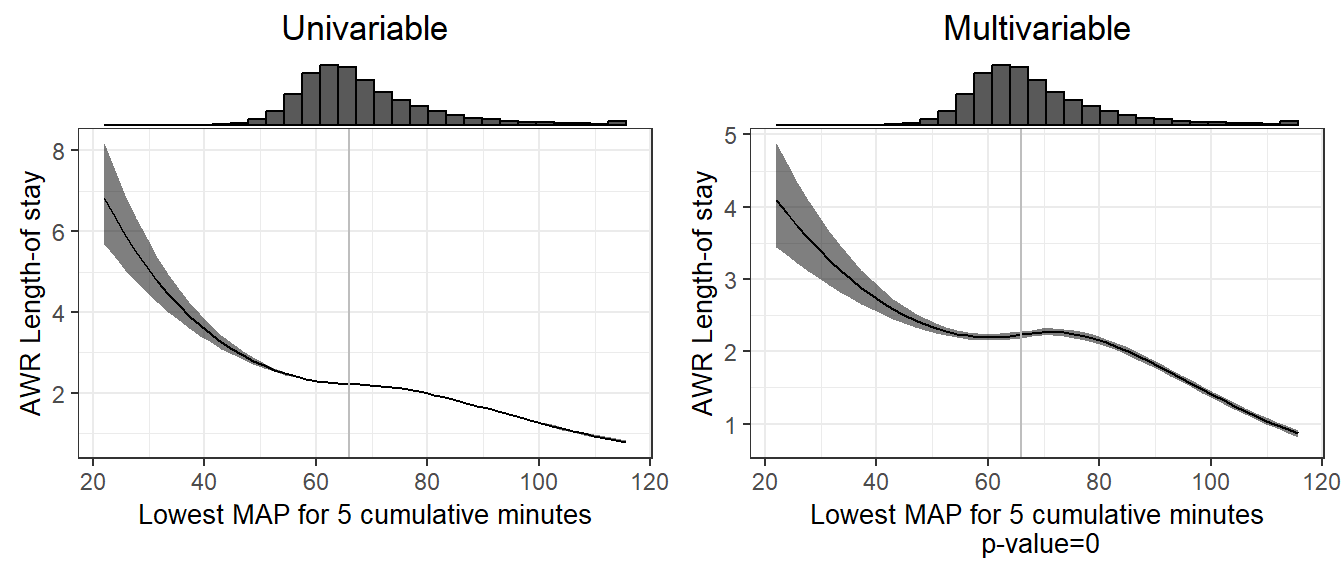

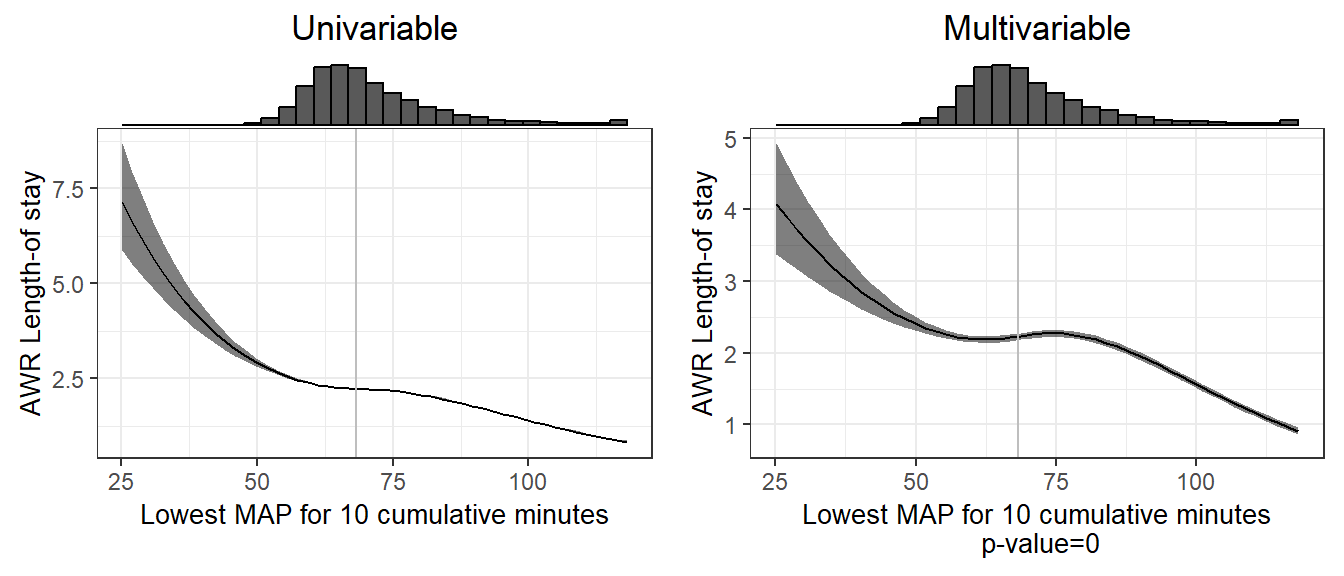

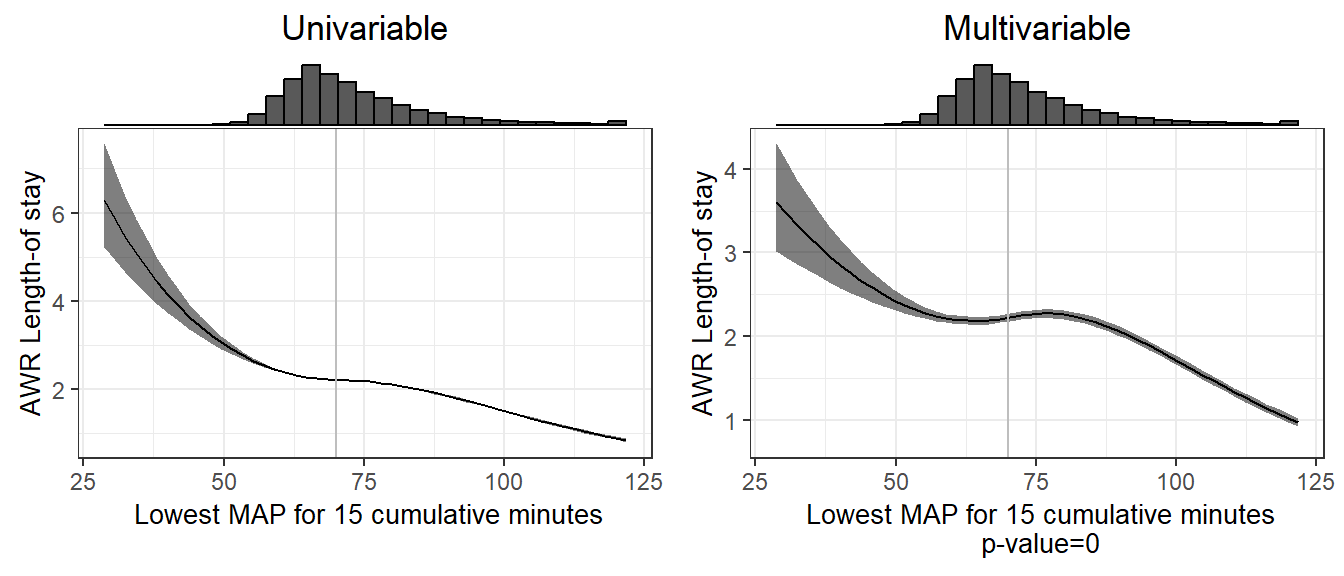

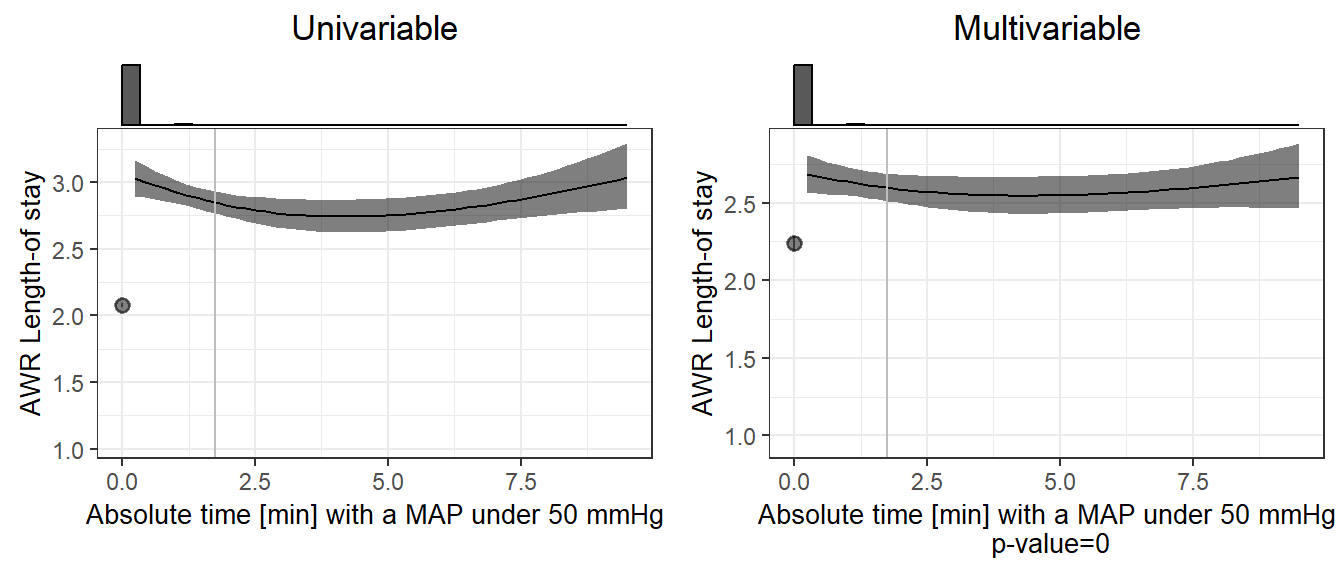

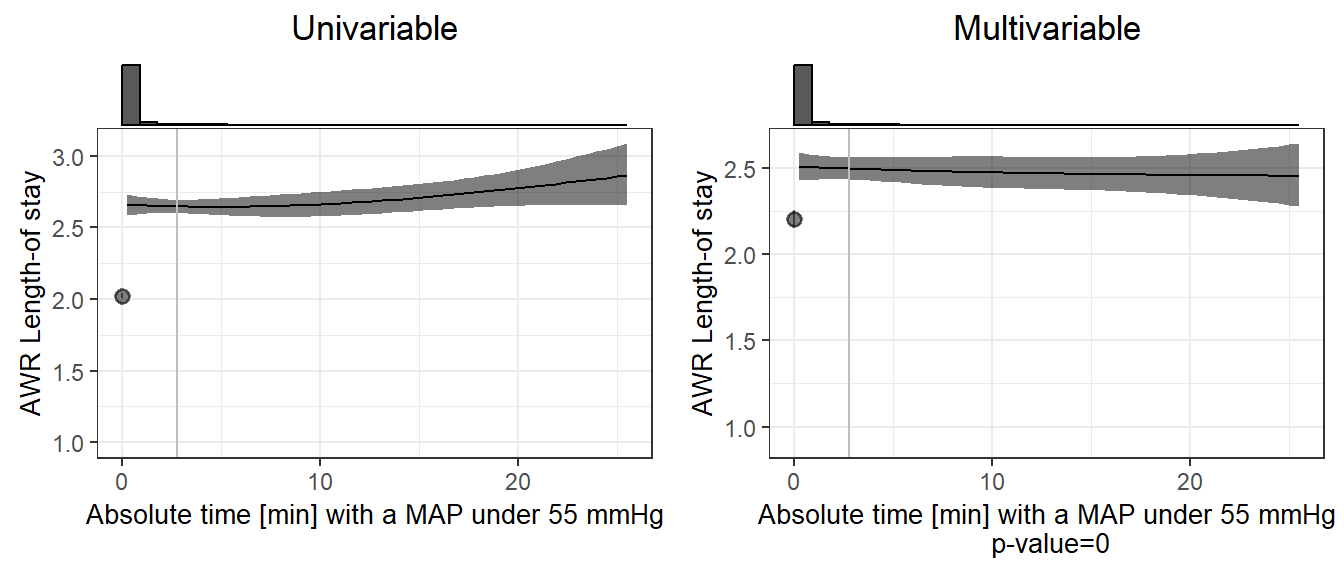

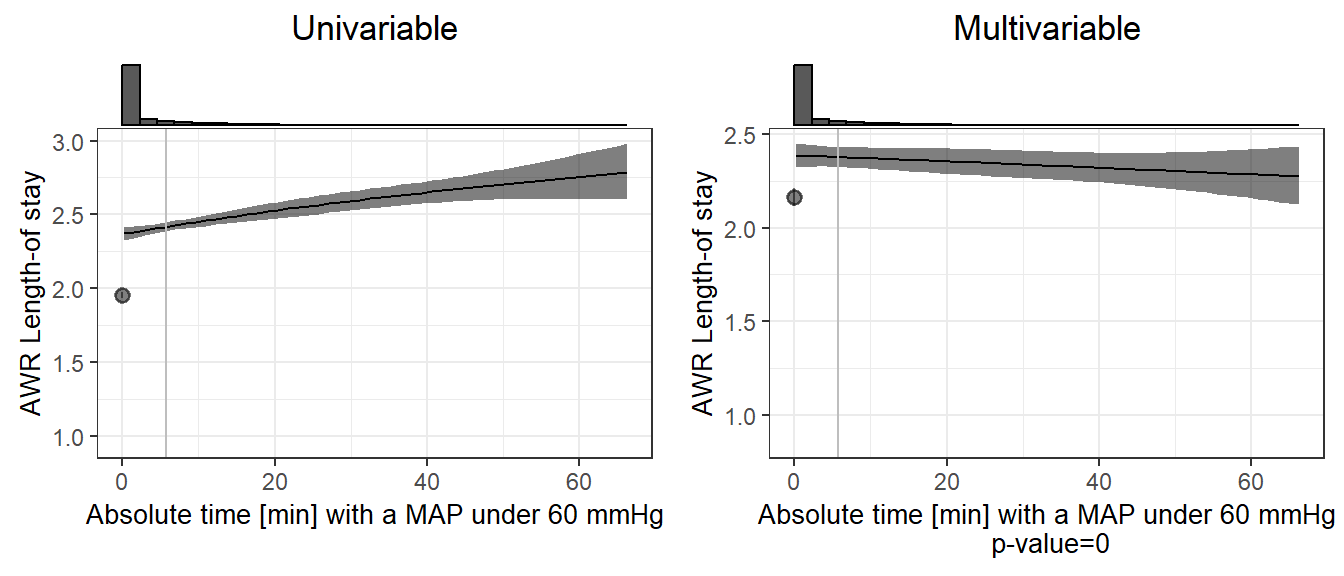

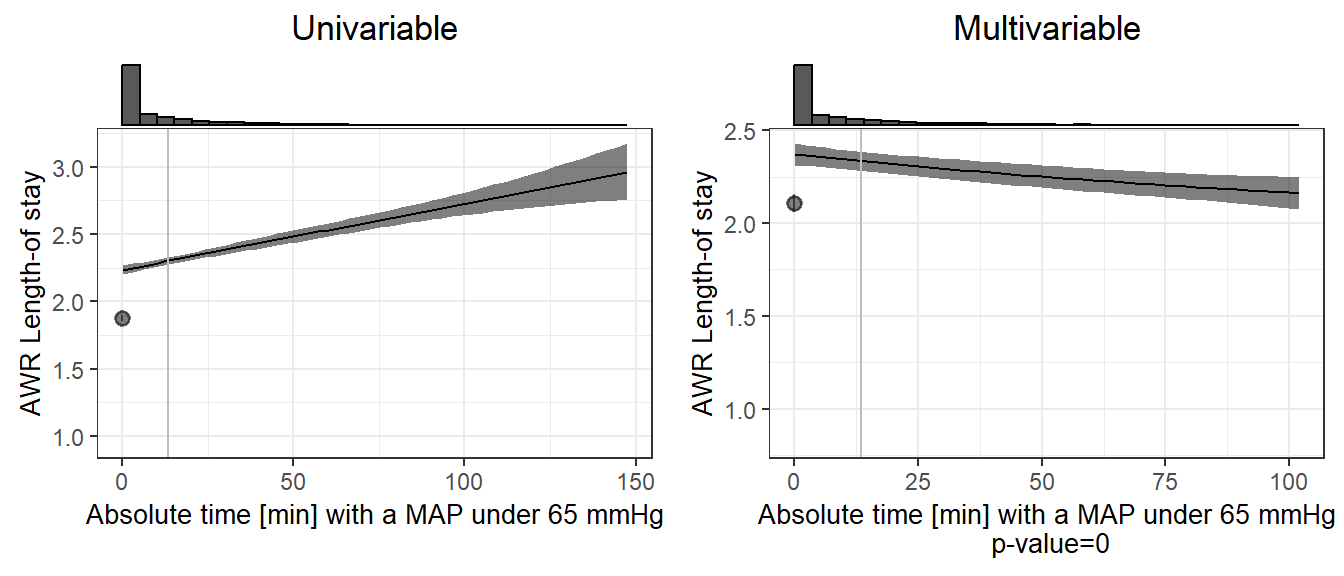

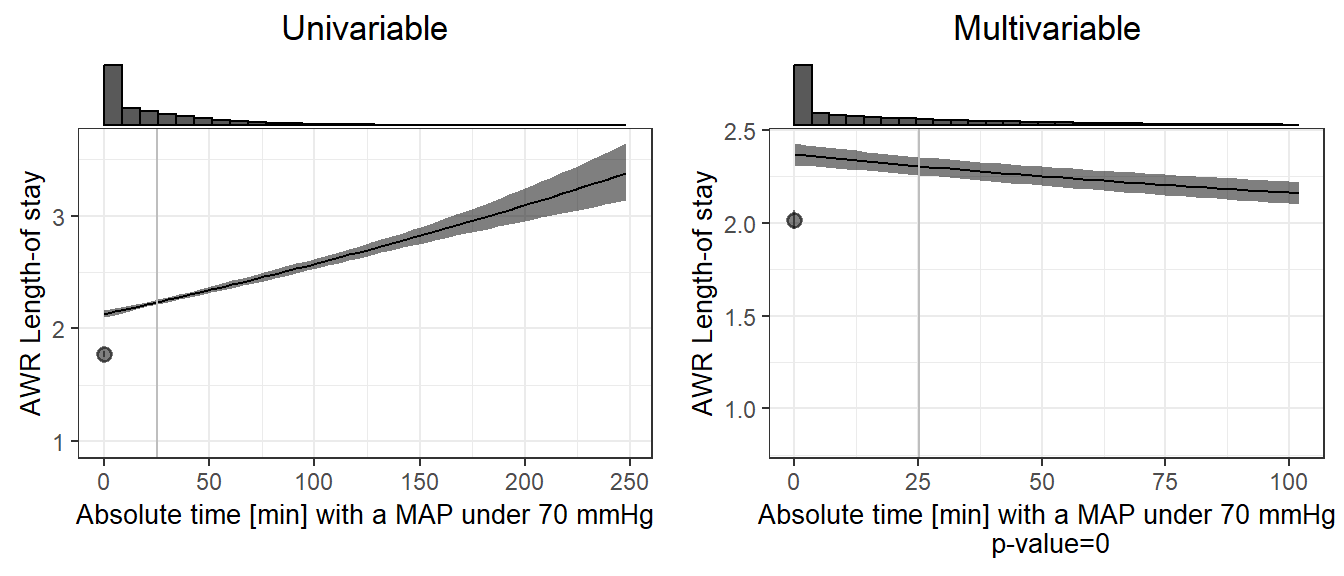

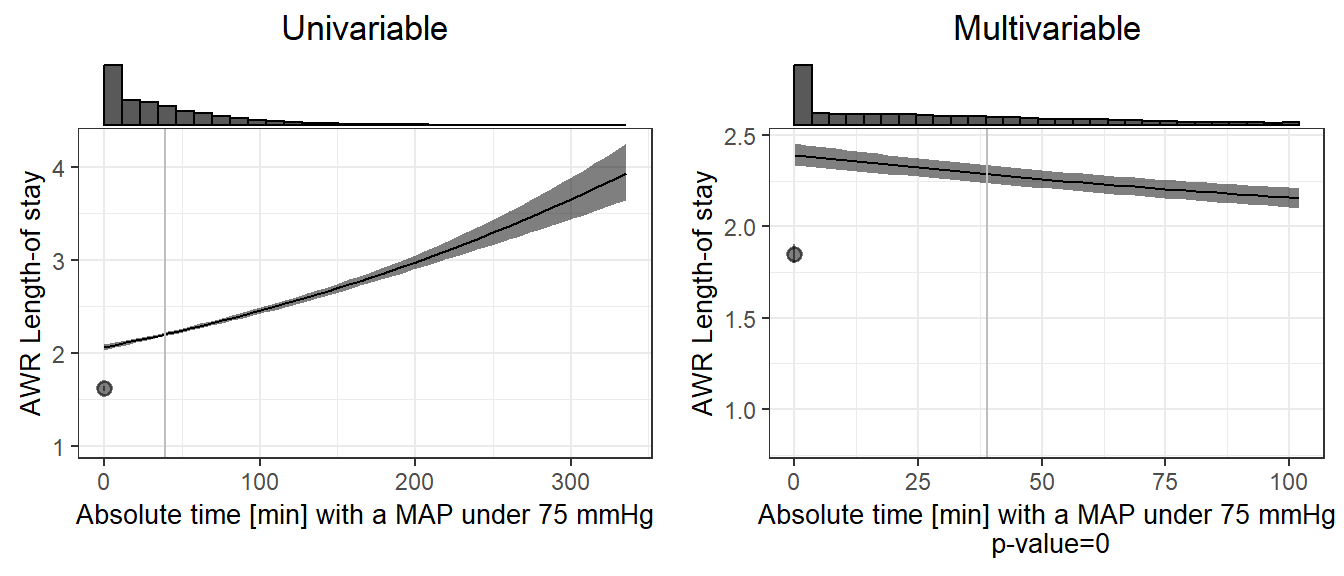

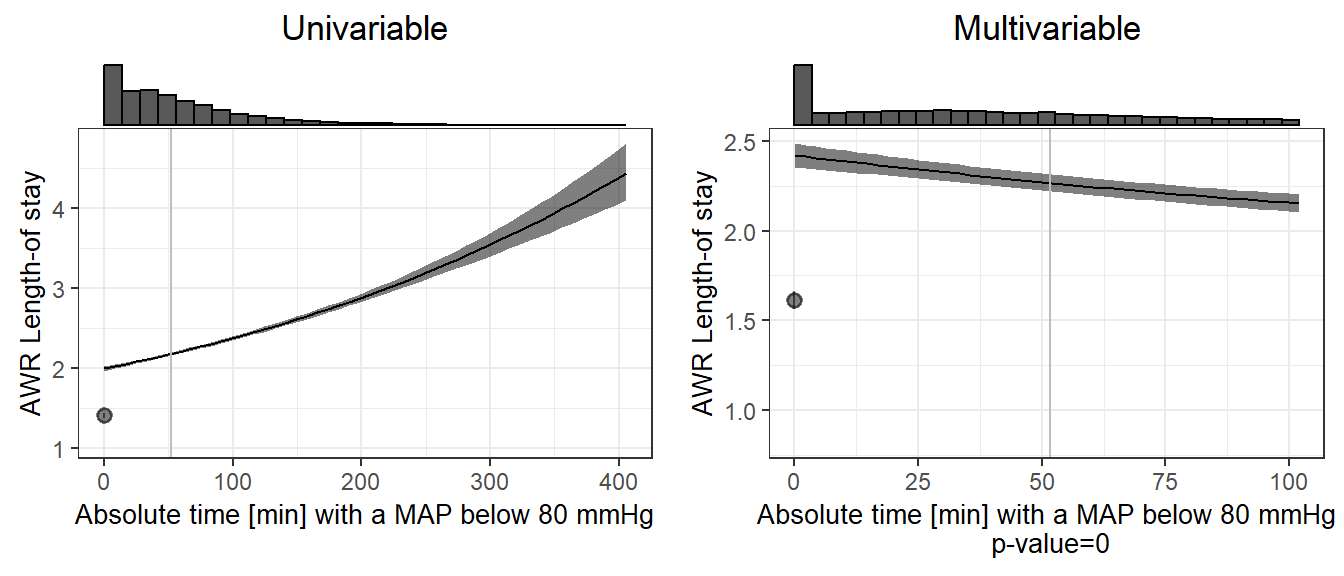

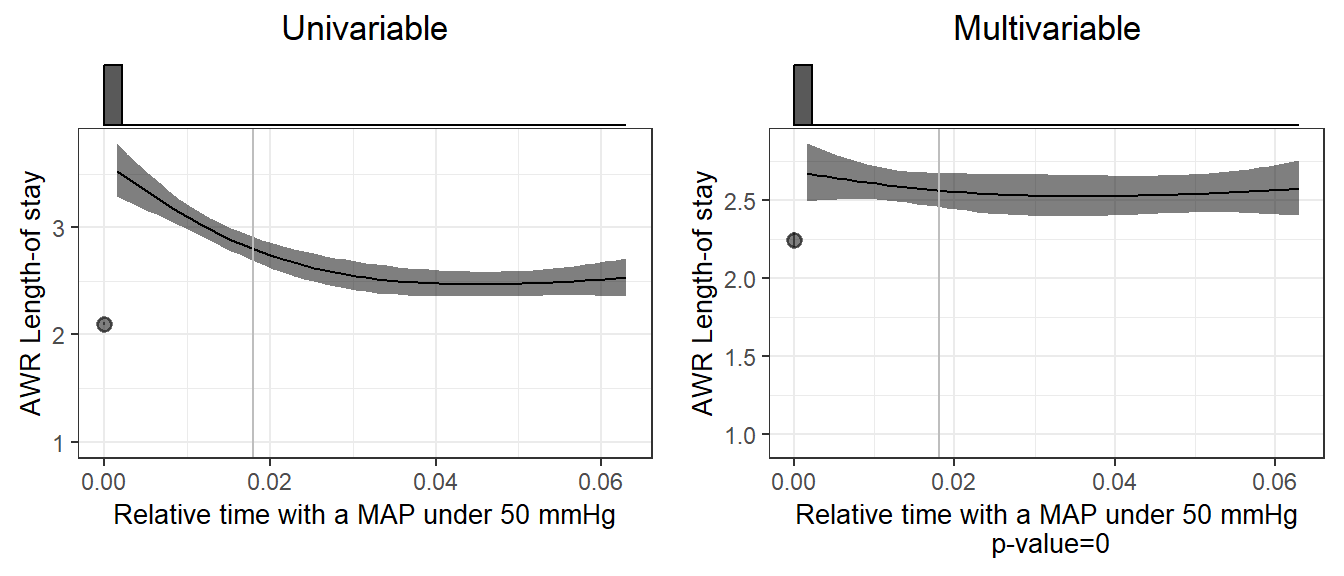

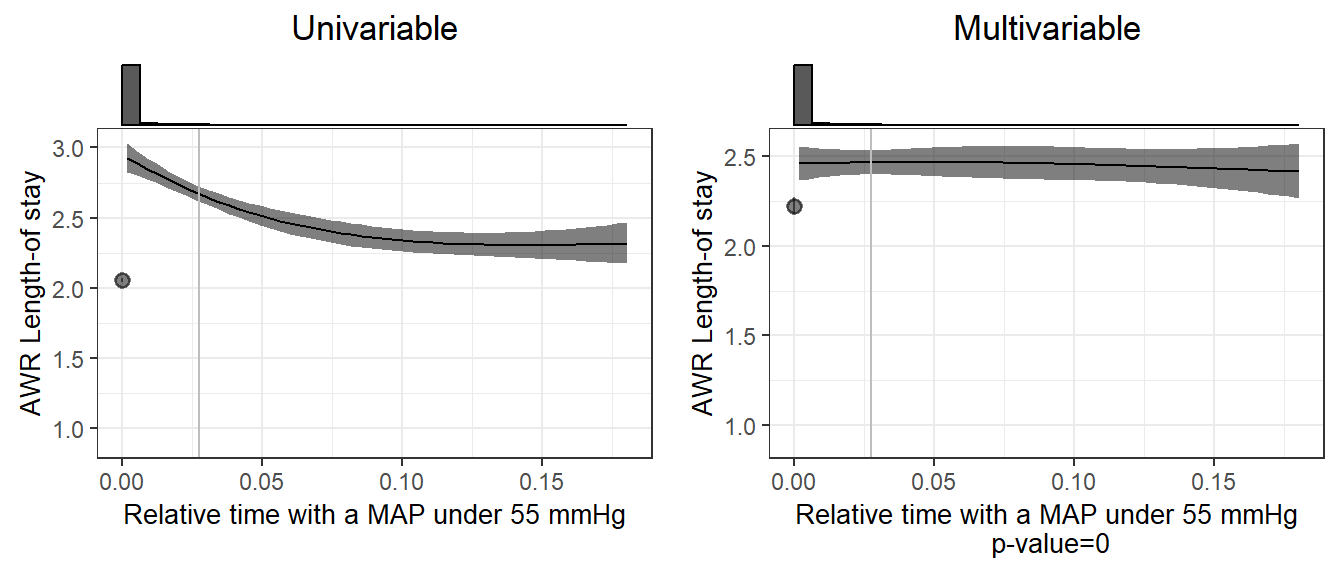

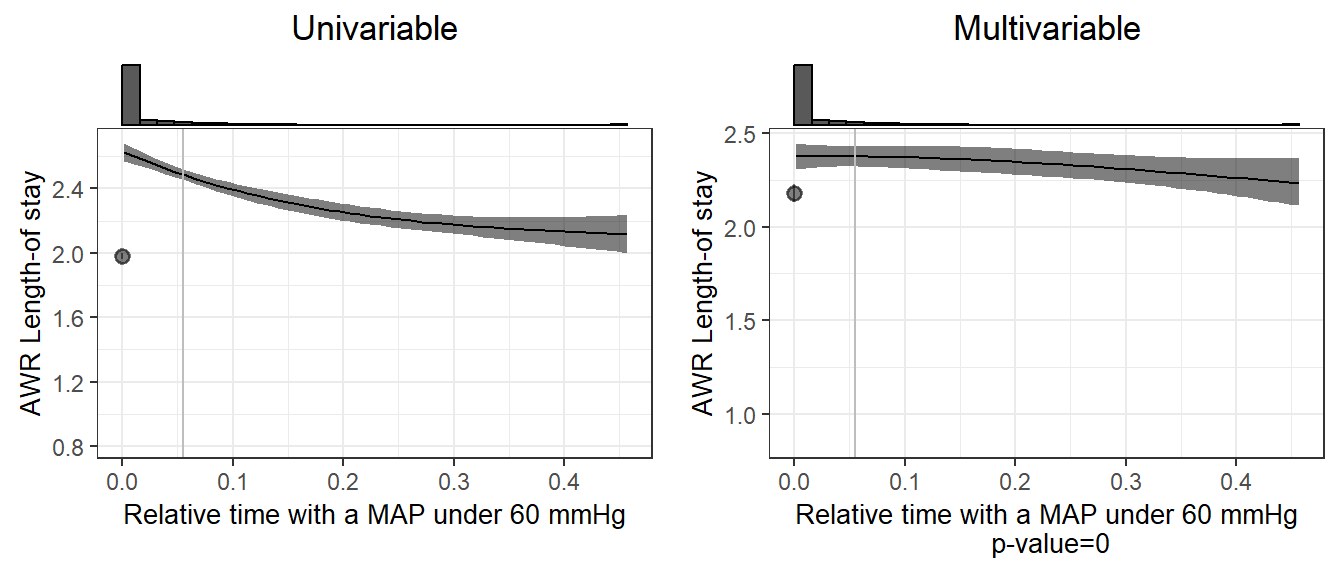

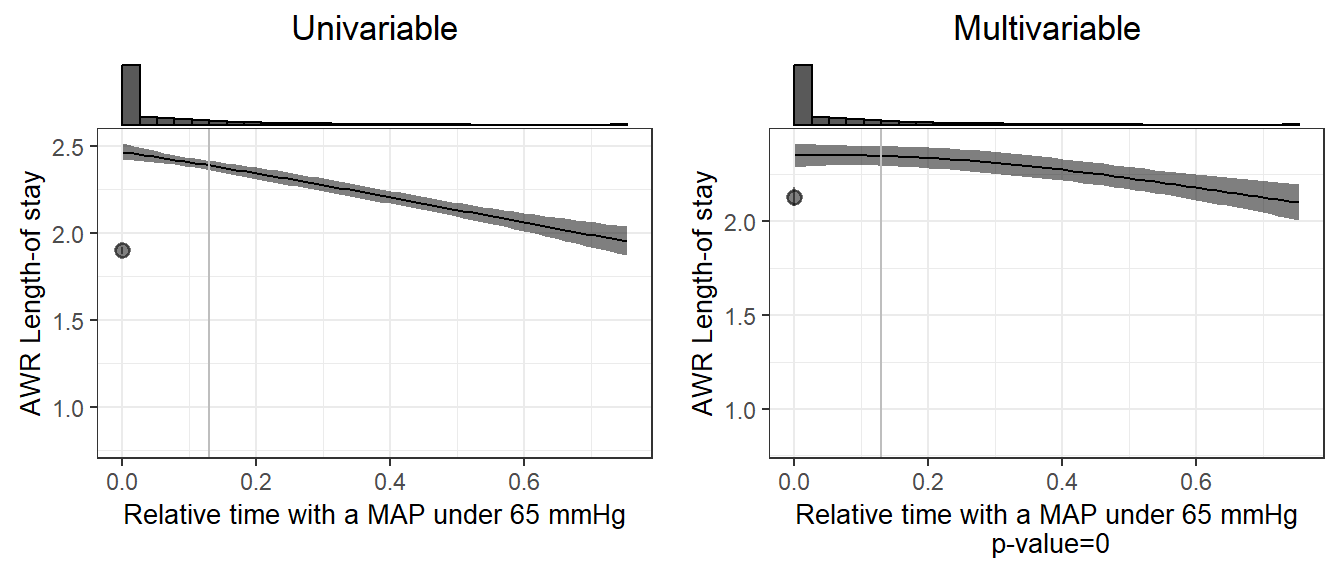

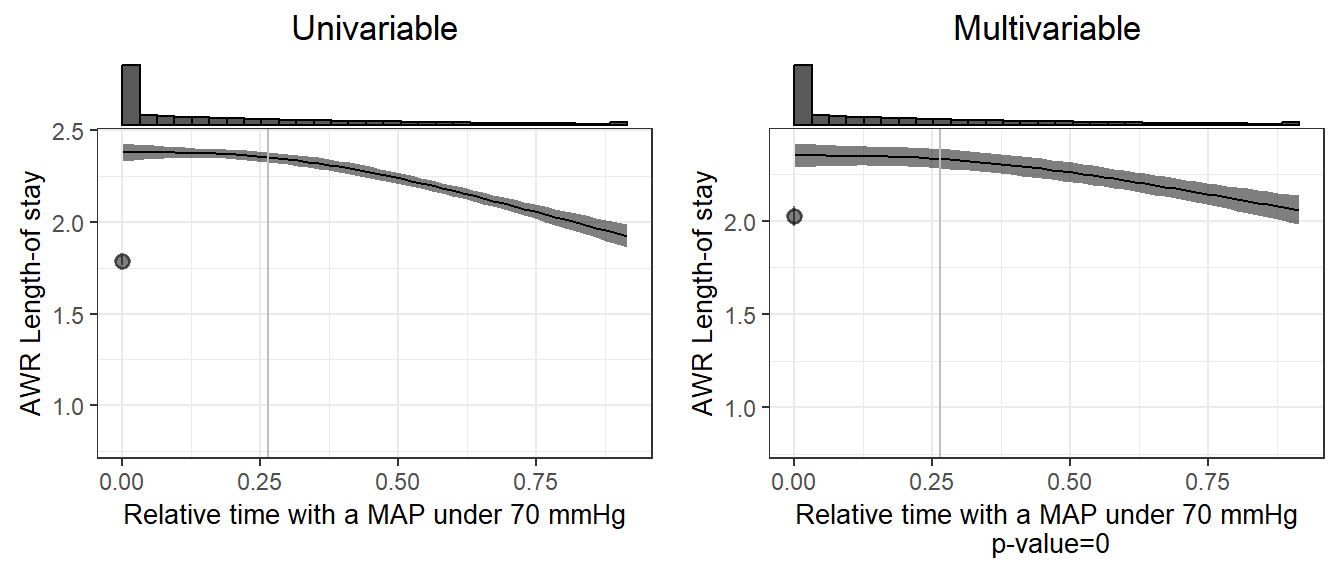

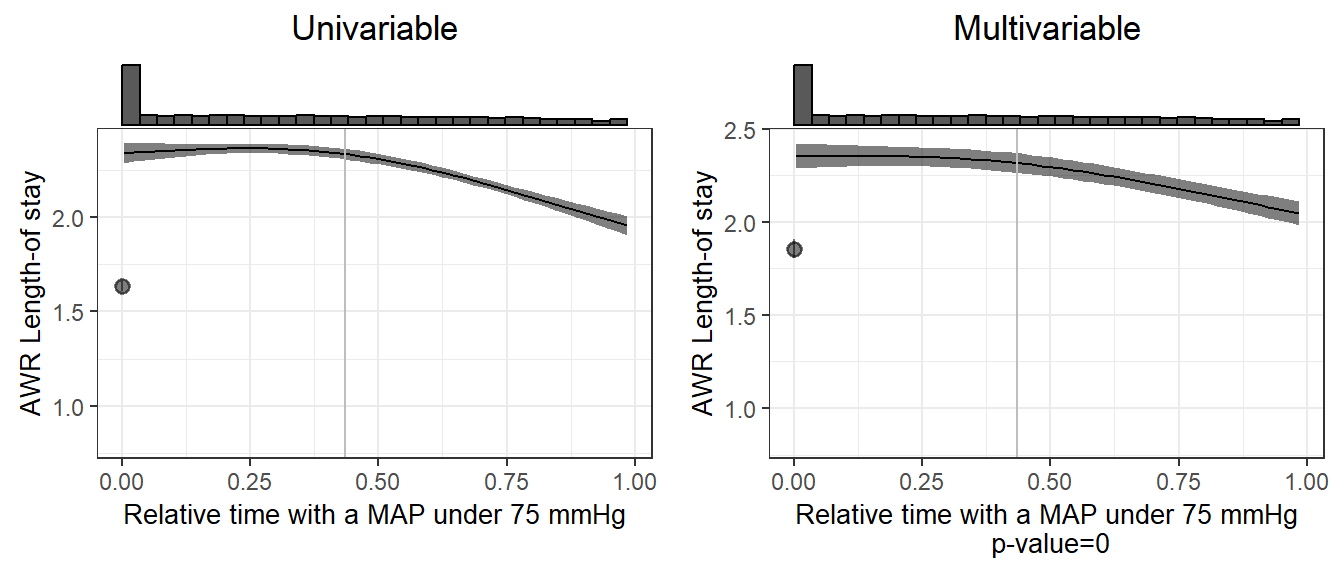

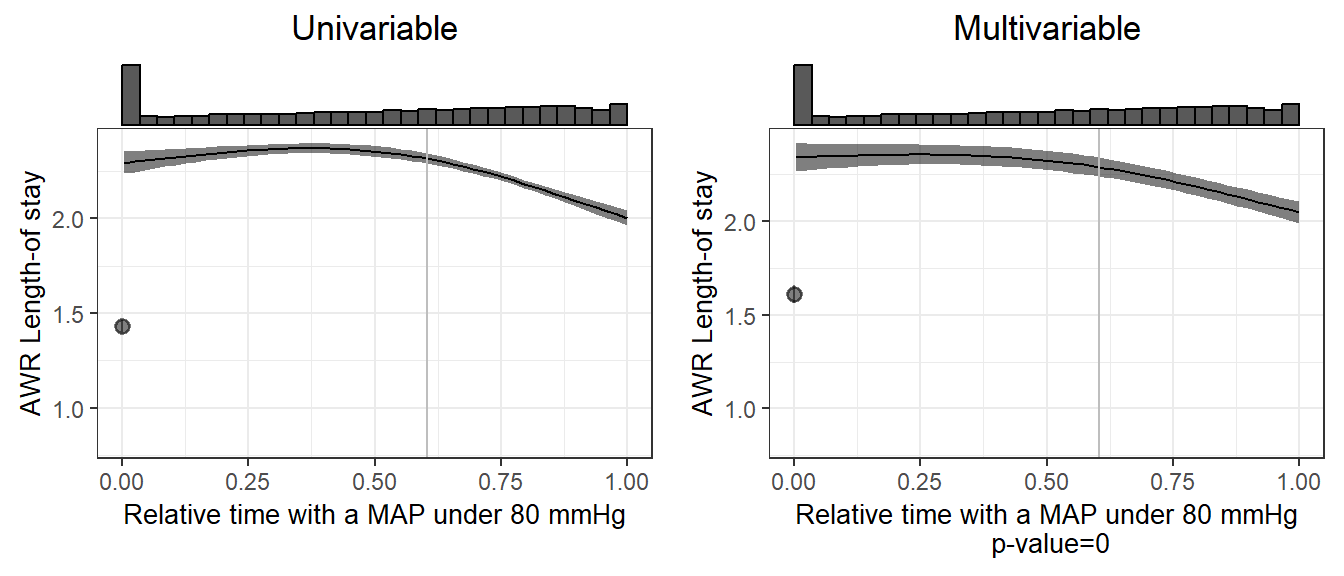


**Mean squared error of all characterizations:**

This figure shows, that low_map_min_1 (Lowest MAP for one cumulative minute) ha the least Mean squared error (MSE) and is therefore selected.


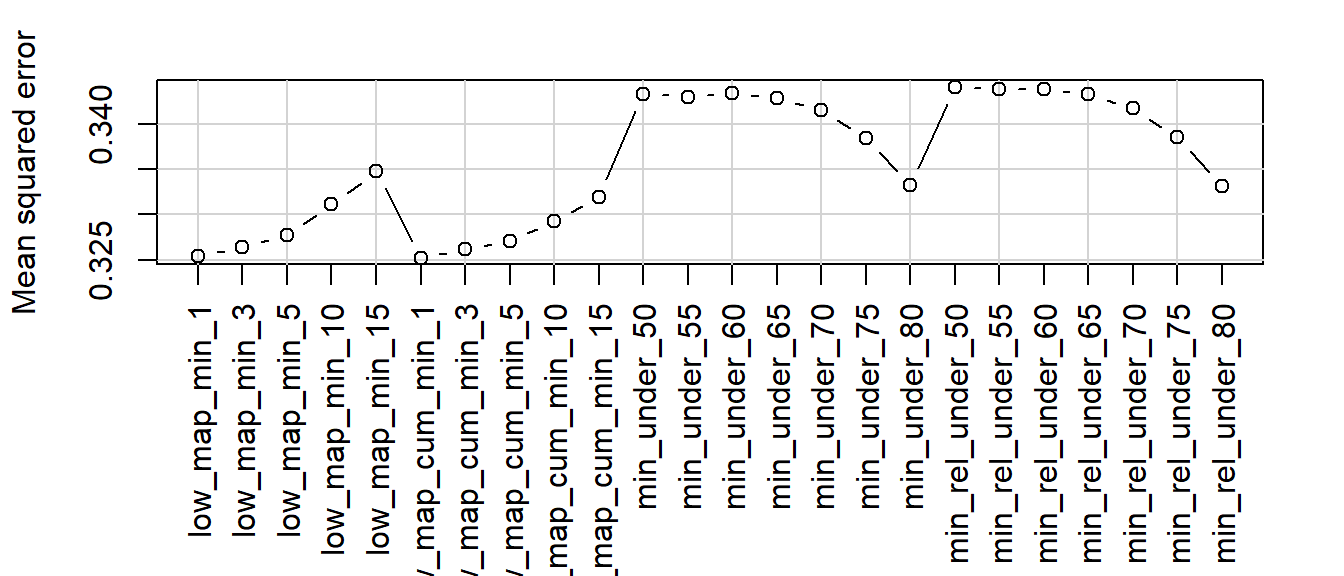


**Multivariate model’s plot (Lowest MAP for one cumulative minute) in the shaping dataest:**


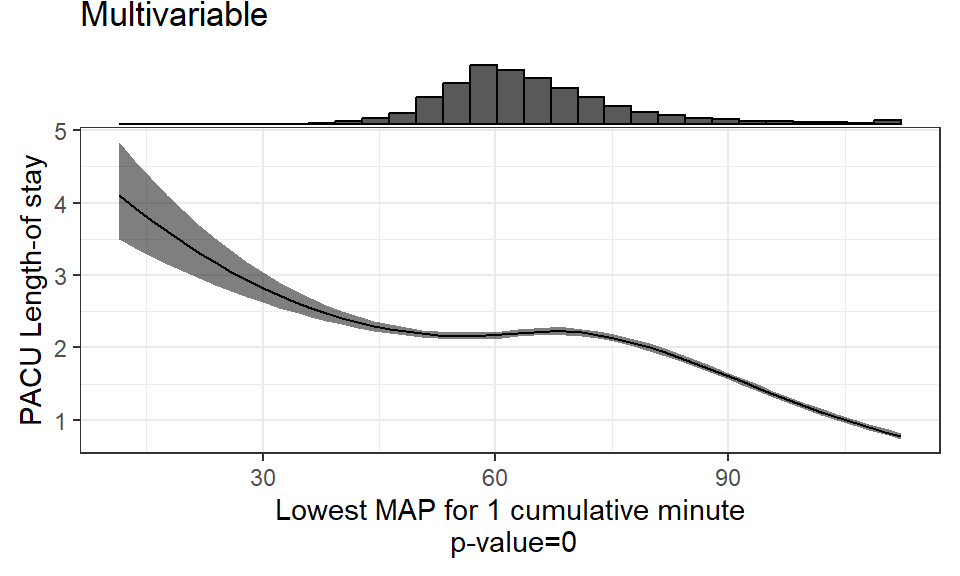


**MSE of the covariates**

Below, MSE of covariates from the modell using “Lowest MAP for one cumulative minute**”** in the Shaping dataset are shown.Gender_0W: female gender, ns: spline, dauerOPh: duration of surgery [h], e_to_surgery: time to surgery, OPgroup: Surgeries speciality, ASA_Status: ASA status, hypo: IOH characterization.


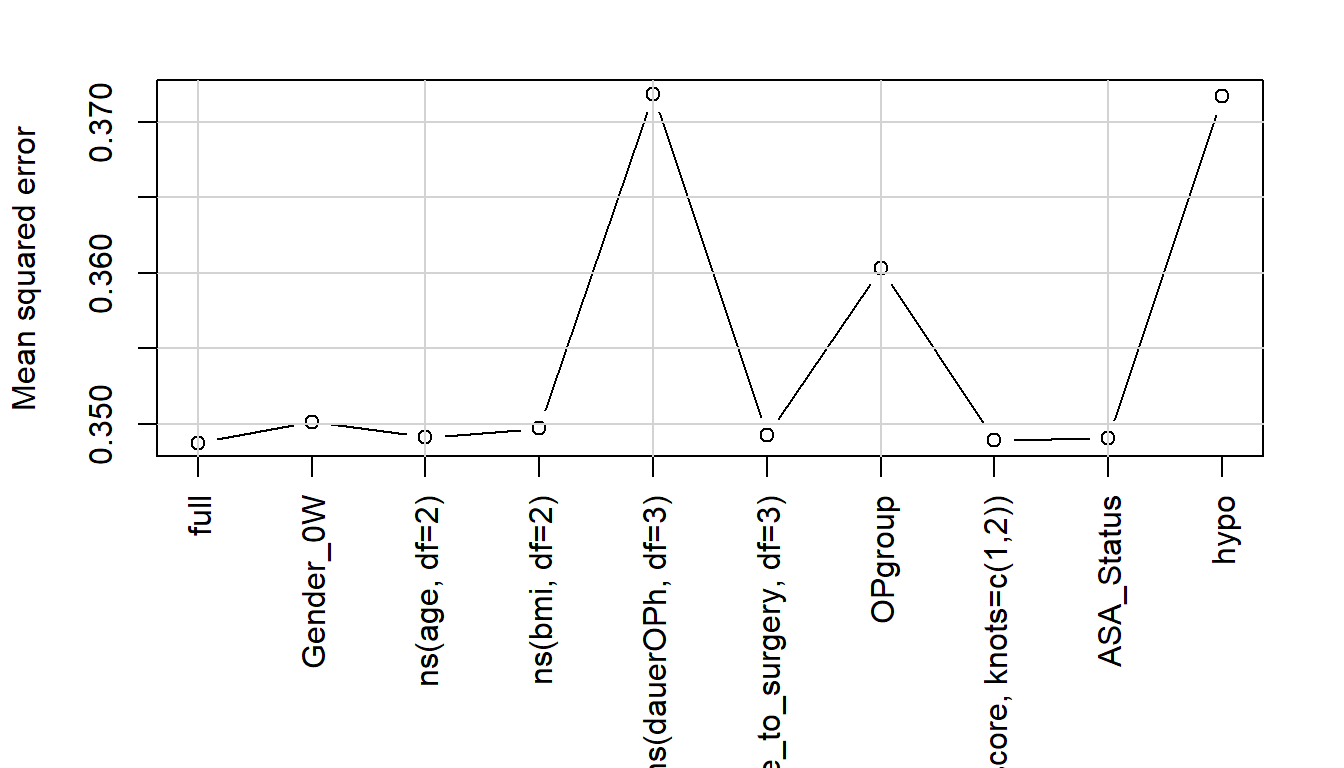

Supplement: S1 File — (DOCX) [file pone.0312966.s001.docx]
