# Supplementary material for: Definition of clinically relevant intraoperative hypotension: A data-driven approach
Source: PLoS One. 2024 Nov 1;19(11):e0312966. doi: 10.1371/journal.pone.0312966 (PMC11530086; doi:10.1371/journal.pone.0312966)
Supplement: S2 File — (DOCX) [file pone.0312966.s002.docx]

**Supplemental Table 2.1: Effect sizes Mortality**

In this table the effect sizes for mortality calculated for clinically relevant values of MAP and time can be found. Value shows the selected values for MAP and time. LCL: Lower confidence interval limit, UCL: Upper confidence interval limit

|  | Value | Probability | LCL | UCL |
| --- | --- | --- | --- | --- |
| Lowest MAP sustained for | [mmHg] |  |  |  |
| 1 minute | 50.00 | 0.067 | 0.037 | 0.120 |
| 1 minute | 65.00 | 0.051 | 0.029 | 0.090 |
| 1 minute | 75.00 | 0.044 | 0.024 | 0.080 |
| 3 minutes | 50.00 | 0.071 | 0.039 | 0.129 |
| 3 minutes | 65.00 | 0.054 | 0.031 | 0.094 |
| 3 minutes | 75.00 | 0.042 | 0.023 | 0.077 |
| 5 minutes | 50.00 | 0.076 | 0.041 | 0.141 |
| 5 minutes | 65.00 | 0.057 | 0.032 | 0.099 |
| 5 minutes | 75.00 | 0.045 | 0.025 | 0.081 |
| 10 minutes | 50.00 | 0.098 | 0.049 | 0.197 |
| 10 minutes | 65.00 | 0.064 | 0.036 | 0.113 |
| 10 minutes | 75.00 | 0.047 | 0.026 | 0.083 |
| 15 minutes | 50.00 | 0.087 | 0.038 | 0.199 |
| 15 minutes | 65.00 | 0.073 | 0.041 | 0.130 |
| 15 minutes | 75.00 | 0.053 | 0.030 | 0.092 |
| Lowest MAP sustained for | [mmHg] |  |  |  |
| 1 cumulative minute | 50.00 | 0.067 | 0.037 | 0.119 |
| 1 cumulative minute | 65.00 | 0.050 | 0.029 | 0.089 |
| 1 cumulative minute | 75.00 | 0.043 | 0.023 | 0.079 |
| 3 cumulative minutes | 50.00 | 0.070 | 0.039 | 0.126 |
| 3 cumulative minutes | 65.00 | 0.052 | 0.030 | 0.092 |
| 3 cumulative minutes | 75.00 | 0.043 | 0.023 | 0.079 |
| 5 cumulative minutes | 50.00 | 0.073 | 0.040 | 0.132 |
| 5 cumulative minutes | 65.00 | 0.054 | 0.031 | 0.095 |
| 5 cumulative minutes | 75.00 | 0.045 | 0.024 | 0.081 |
| 10 cumulative minutes | 50.00 | 0.084 | 0.045 | 0.156 |
| 10 cumulative minutes | 65.00 | 0.056 | 0.032 | 0.098 |
| 10 cumulative minutes | 75.00 | 0.048 | 0.026 | 0.085 |
| 15 cumulative minutes | 50.00 | 0.097 | 0.050 | 0.188 |
| 15 cumulative minutes | 65.00 | 0.058 | 0.033 | 0.103 |
| 15 cumulative minutes | 75.00 | 0.048 | 0.027 | 0.085 |
| Absolute time [min] with a MAP | [min] |  |  |  |
| under 50 mmHg | 5.00 | 0.059 | 0.029 | 0.121 |
| under 50 mmHg | 10.00 | 0.130 | 0.059 | 0.286 |
| under 55 mmHg | 5.00 | 0.056 | 0.030 | 0.103 |
| under 55 mmHg | 10.00 | 0.056 | 0.029 | 0.108 |
| under 60 mmHg | 5.00 | 0.066 | 0.037 | 0.117 |
| under 60 mmHg | 10.00 | 0.064 | 0.036 | 0.113 |
| under 65 mmHg | 5.00 | 0.063 | 0.035 | 0.112 |
| under 65 mmHg | 10.00 | 0.062 | 0.035 | 0.110 |
| under 70 mmHg | 5.00 | 0.055 | 0.031 | 0.098 |
| under 70 mmHg | 10.00 | 0.056 | 0.031 | 0.099 |
| under 75 mmHg | 5.00 | 0.046 | 0.025 | 0.085 |
| under 75 mmHg | 10.00 | 0.048 | 0.026 | 0.086 |
| under 80 mmHg | 5.00 | 0.044 | 0.023 | 0.081 |
| under 80 mmHg | 10.00 | 0.045 | 0.024 | 0.082 |
| Relative time with a MAP |  |  |  |  |
| under 50 mmHg | 0.05 | 0.089 | 0.045 | 0.176 |
| under 50 mmHg | 0.10 | 0.154 | 0.029 | 0.804 |
| under 55 mmHg | 0.05 | 0.056 | 0.030 | 0.107 |
| under 55 mmHg | 0.10 | 0.069 | 0.035 | 0.135 |
| under 60 mmHg | 0.05 | 0.063 | 0.035 | 0.112 |
| under 60 mmHg | 0.10 | 0.062 | 0.034 | 0.112 |
| under 65 mmHg | 0.05 | 0.055 | 0.030 | 0.098 |
| under 65 mmHg | 0.10 | 0.057 | 0.032 | 0.101 |
| under 70 mmHg | 0.05 | 0.050 | 0.028 | 0.092 |
| under 70 mmHg | 0.10 | 0.051 | 0.029 | 0.091 |
| under 75 mmHg | 0.05 | 0.040 | 0.021 | 0.077 |
| under 75 mmHg | 0.10 | 0.041 | 0.022 | 0.077 |
| under 80 mmHg | 0.05 | 0.039 | 0.020 | 0.079 |
| under 80 mmHg | 0.10 | 0.040 | 0.021 | 0.077 |

**Supplemental Table 2.2: Effect sizes Length of Stay**

In this table the effect sizes for mortality calculated for clinically relevant values of MAP and time can be found. Value shows the selected values for MAP and time. LCL: Lower confidence interval limit, UCL: Upper confidence interval limit

|  | Value | Probability | LCL | UCL |
| --- | --- | --- | --- | --- |
| Lowest MAP sustained for | [mmHg] |  |  |  |
| 1 minute | 50.00 | 2.84 | 2.73 | 2.95 |
| 1 minute | 65.00 | 2.81 | 2.72 | 2.92 |
| 1 minute | 75.00 | 2.80 | 2.69 | 2.92 |
| 3 minutes | 50.00 | 2.90 | 2.77 | 3.03 |
| 3 minutes | 65.00 | 2.79 | 2.70 | 2.89 |
| 3 minutes | 75.00 | 2.86 | 2.75 | 2.97 |
| 5 minutes | 50.00 | 2.97 | 2.82 | 3.13 |
| 5 minutes | 65.00 | 2.77 | 2.67 | 2.87 |
| 5 minutes | 75.00 | 2.88 | 2.77 | 2.99 |
| 10 minutes | 50.00 | 3.09 | 2.85 | 3.34 |
| 10 minutes | 65.00 | 2.73 | 2.63 | 2.83 |
| 10 minutes | 75.00 | 2.87 | 2.76 | 2.97 |
| 15 minutes | 50.00 | 3.13 | 2.84 | 3.46 |
| 15 minutes | 65.00 | 2.73 | 2.63 | 2.83 |
| 15 minutes | 75.00 | 2.81 | 2.71 | 2.91 |
| Lowest MAP sustained for | [mmHg] |  |  |  |
| 1 cumulative minute | 50.00 | 2.83 | 2.72 | 2.94 |
| 1 cumulative minute | 65.00 | 2.81 | 2.72 | 2.92 |
| 1 cumulative minute | 75.00 | 2.79 | 2.68 | 2.91 |
| 3 cumulative minutes | 50.00 | 2.90 | 2.77 | 3.02 |
| 3 cumulative minutes | 65.00 | 2.80 | 2.70 | 2.90 |
| 3 cumulative minutes | 75.00 | 2.84 | 2.73 | 2.95 |
| 5 cumulative minutes | 50.00 | 2.97 | 2.83 | 3.11 |
| 5 cumulative minutes | 65.00 | 2.78 | 2.69 | 2.88 |
| 5 cumulative minutes | 75.00 | 2.86 | 2.75 | 2.97 |
| 10 cumulative minutes | 50.00 | 3.13 | 2.94 | 3.32 |
| 10 cumulative minutes | 65.00 | 2.74 | 2.65 | 2.84 |
| 10 cumulative minutes | 75.00 | 2.88 | 2.78 | 2.99 |
| 15 cumulative minutes | 50.00 | 3.24 | 3.01 | 3.48 |
| 15 cumulative minutes | 65.00 | 2.74 | 2.64 | 2.84 |
| 15 cumulative minutes | 75.00 | 2.86 | 2.76 | 2.96 |
| Absolute time [min] with a MAP | [min] |  |  |  |
| under 50 mmHg | 5.00 | 3.15 | 2.94 | 3.37 |
| under 50 mmHg | 10.00 | 3.23 | 2.88 | 3.63 |
| under 55 mmHg | 5.00 | 3.08 | 2.94 | 3.22 |
| under 55 mmHg | 10.00 | 3.18 | 3.01 | 3.36 |
| under 60 mmHg | 5.00 | 2.88 | 2.78 | 3.00 |
| under 60 mmHg | 10.00 | 2.92 | 2.81 | 3.04 |
| under 65 mmHg | 5.00 | 2.85 | 2.75 | 2.96 |
| under 65 mmHg | 10.00 | 2.86 | 2.75 | 2.96 |
| under 70 mmHg | 5.00 | 2.85 | 2.74 | 2.96 |
| under 70 mmHg | 10.00 | 2.84 | 2.74 | 2.95 |
| under 75 mmHg | 5.00 | 2.91 | 2.80 | 3.03 |
| under 75 mmHg | 10.00 | 2.90 | 2.79 | 3.01 |
| under 80 mmHg | 5.00 | 2.98 | 2.86 | 3.11 |
| under 80 mmHg | 10.00 | 2.97 | 2.85 | 3.09 |
| Relative time [min] with a MAP |  |  |  |  |
| under 50 mmHg | 0.05 | 3.19 | 2.97 | 3.43 |
| under 50 mmHg | 0.10 | 2.99 | 2.30 | 3.89 |
| under 55 mmHg | 0.05 | 3.08 | 2.93 | 3.24 |
| under 55 mmHg | 0.10 | 3.18 | 3.00 | 3.37 |
| under 60 mmHg | 0.05 | 2.89 | 2.78 | 3.00 |
| under 60 mmHg | 0.10 | 2.92 | 2.80 | 3.04 |
| under 65 mmHg | 0.05 | 2.83 | 2.73 | 2.94 |
| under 65 mmHg | 0.10 | 2.87 | 2.77 | 2.97 |
| under 70 mmHg | 0.05 | 2.83 | 2.72 | 2.94 |
| under 70 mmHg | 0.10 | 2.85 | 2.74 | 2.96 |
| under 75 mmHg | 0.05 | 2.91 | 2.78 | 3.04 |
| under 75 mmHg | 0.10 | 2.91 | 2.79 | 3.03 |
| under 80 mmHg | 0.05 | 2.97 | 2.83 | 3.12 |
| under 80 mmHg | 0.10 | 2.96 | 2.83 | 3.10 |

**Supplemental Table 2.3: Effect sizes Length of PACU-Stay**

In this table the effect sizes for mortality calculated for clinically relevant values of MAP and time can be found. Value shows the selected values for MAP and time. LCL: Lower confidence interval limit, UCL: Upper confidence interval limit

|  | Value | Probability | LCL | UCL |
| --- | --- | --- | --- | --- |
| Lowest MAP sustained for | [mmHg] |  |  |  |
| 1 minute | 50.00 | 2.25 | 2.20 | 2.31 |
| 1 minute | 65.00 | 2.25 | 2.21 | 2.30 |
| 1 minute | 75.00 | 2.18 | 2.13 | 2.23 |
| 3 minutes | 50.00 | 2.28 | 2.22 | 2.35 |
| 3 minutes | 65.00 | 2.23 | 2.19 | 2.28 |
| 3 minutes | 75.00 | 2.25 | 2.20 | 2.30 |
| 5 minutes | 50.00 | 2.30 | 2.22 | 2.38 |
| 5 minutes | 65.00 | 2.21 | 2.16 | 2.26 |
| 5 minutes | 75.00 | 2.29 | 2.23 | 2.34 |
| 10 minutes | 50.00 | 2.31 | 2.19 | 2.43 |
| 10 minutes | 65.00 | 2.17 | 2.13 | 2.22 |
| 10 minutes | 75.00 | 2.29 | 2.24 | 2.34 |
| 15 minutes | 50.00 | 2.25 | 2.11 | 2.40 |
| 15 minutes | 65.00 | 2.16 | 2.12 | 2.21 |
| 15 minutes | 75.00 | 2.26 | 2.22 | 2.31 |
| Lowest MAP sustained for | [mmHg] |  |  |  |
| 1 cumulative minute | 50.00 | 2.26 | 2.21 | 2.31 |
| 1 cumulative minute | 65.00 | 2.25 | 2.20 | 2.30 |
| 1 cumulative minute | 75.00 | 2.17 | 2.12 | 2.22 |
| 3 cumulative minutes | 50.00 | 2.30 | 2.24 | 2.36 |
| 3 cumulative minutes | 65.00 | 2.24 | 2.19 | 2.28 |
| 3 cumulative minutes | 75.00 | 2.22 | 2.17 | 2.28 |
| 5 cumulative minutes | 50.00 | 2.34 | 2.27 | 2.41 |
| 5 cumulative minutes | 65.00 | 2.22 | 2.18 | 2.27 |
| 5 cumulative minutes | 75.00 | 2.25 | 2.20 | 2.30 |
| 10 cumulative minutes | 50.00 | 2.40 | 2.31 | 2.50 |
| 10 cumulative minutes | 65.00 | 2.20 | 2.15 | 2.24 |
| 10 cumulative minutes | 75.00 | 2.28 | 2.23 | 2.33 |
| 15 cumulative minutes | 50.00 | 2.42 | 2.31 | 2.54 |
| 15 cumulative minutes | 65.00 | 2.19 | 2.14 | 2.23 |
| 15 cumulative minutes | 75.00 | 2.27 | 2.22 | 2.32 |
| Absolute time [min] with a MAP | [min] |  |  |  |
| under 50 mmHg | 5.00 | 2.55 | 2.43 | 2.67 |
| under 50 mmHg | 10.00 | 2.68 | 2.46 | 2.93 |
| under 55 mmHg | 5.00 | 2.49 | 2.42 | 2.56 |
| under 55 mmHg | 10.00 | 2.47 | 2.39 | 2.57 |
| under 60 mmHg | 5.00 | 2.38 | 2.33 | 2.44 |
| under 60 mmHg | 10.00 | 2.37 | 2.32 | 2.43 |
| under 65 mmHg | 5.00 | 2.36 | 2.31 | 2.41 |
| under 65 mmHg | 10.00 | 2.35 | 2.29 | 2.40 |
| under 70 mmHg | 5.00 | 2.36 | 2.30 | 2.41 |
| under 70 mmHg | 10.00 | 2.35 | 2.29 | 2.40 |
| under 75 mmHg | 5.00 | 2.38 | 2.32 | 2.44 |
| under 75 mmHg | 10.00 | 2.37 | 2.31 | 2.42 |
| under 80 mmHg | 5.00 | 2.40 | 2.34 | 2.47 |
| under 80 mmHg | 10.00 | 2.39 | 2.33 | 2.45 |
| Relative time [min] with a MAP | |  |  |  |
| under 50 mmHg | 0.05 | 2.54 | 2.42 | 2.67 |
| under 50 mmHg | 0.10 | 2.67 | 2.22 | 3.22 |
| under 55 mmHg | 0.05 | 2.47 | 2.39 | 2.55 |
| under 55 mmHg | 0.10 | 2.46 | 2.37 | 2.55 |
| under 60 mmHg | 0.05 | 2.38 | 2.32 | 2.43 |
| under 60 mmHg | 0.10 | 2.37 | 2.31 | 2.43 |
| under 65 mmHg | 0.05 | 2.35 | 2.30 | 2.41 |
| under 65 mmHg | 0.10 | 2.35 | 2.30 | 2.40 |
| under 70 mmHg | 0.05 | 2.35 | 2.29 | 2.41 |
| under 70 mmHg | 0.10 | 2.35 | 2.30 | 2.40 |
| under 75 mmHg | 0.05 | 2.35 | 2.29 | 2.42 |
| under 75 mmHg | 0.10 | 2.35 | 2.30 | 2.41 |
| under 80 mmHg | 0.05 | 2.35 | 2.28 | 2.41 |
| under 80 mmHg | 0.10 | 2.35 | 2.29 | 2.41 |

**Supplemental Table 2.4: Missing information:**

| Missing information | |
| --- | --- |
| Surgical specialty | 505 |
| Gender | 38 |
| ASA status | 233 |
| BMI | 31 |
| SUM | 807 |

This table shows the details of reasons for patient exclusion due to missing information.

**Supplemental Figure 2.5: Interactions Age, ASA and PACU-LOS**
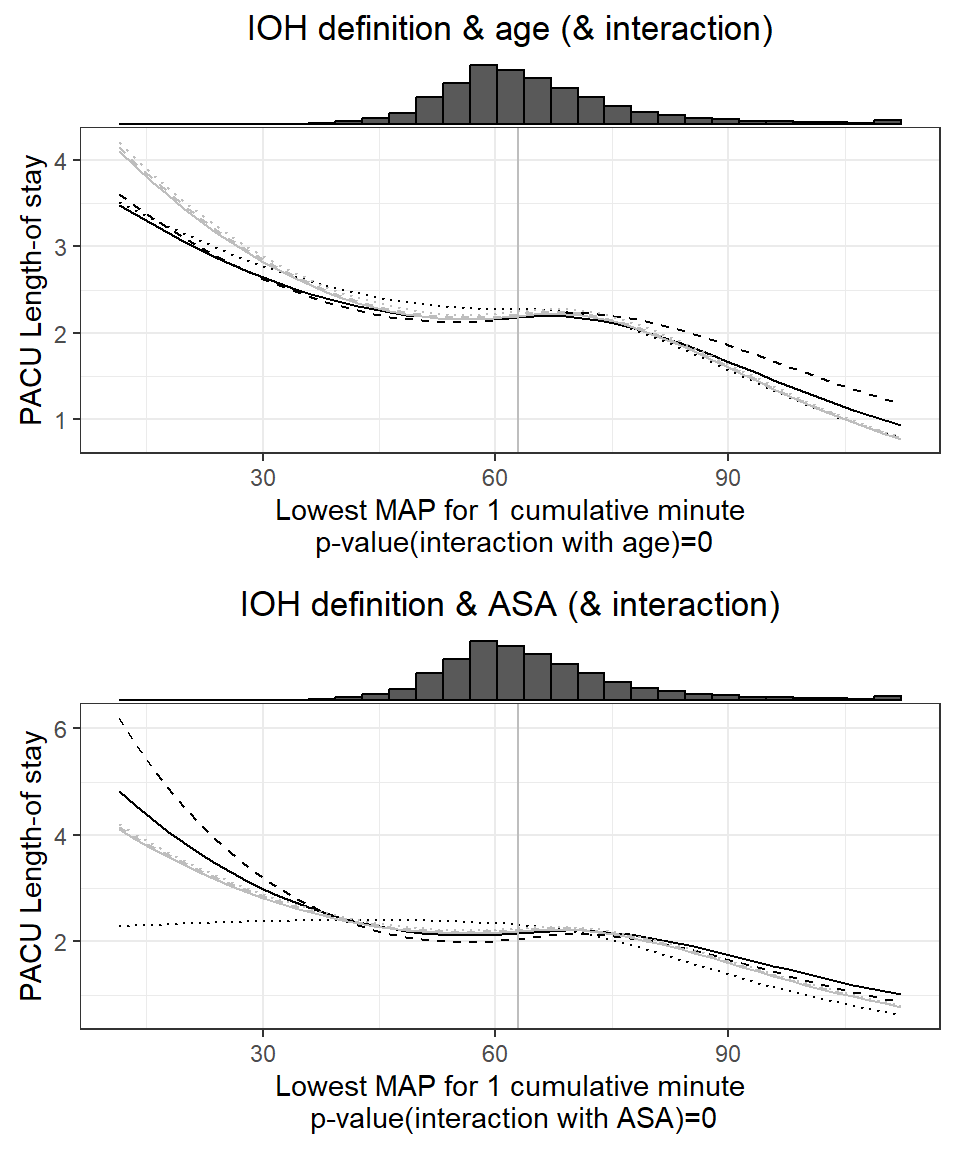


We test the clinically plausible interactions of the chosen operationalization with age (grey = model prediction without interaction, black = model prediction with interaction, solid = at median age/ASA2, dashed = at Q1 of age/ASA1, dotted = at Q3 of age/ASA3)

**Supplemental Figure 2.6: Interactions Age, ASA and PACU-LOS**


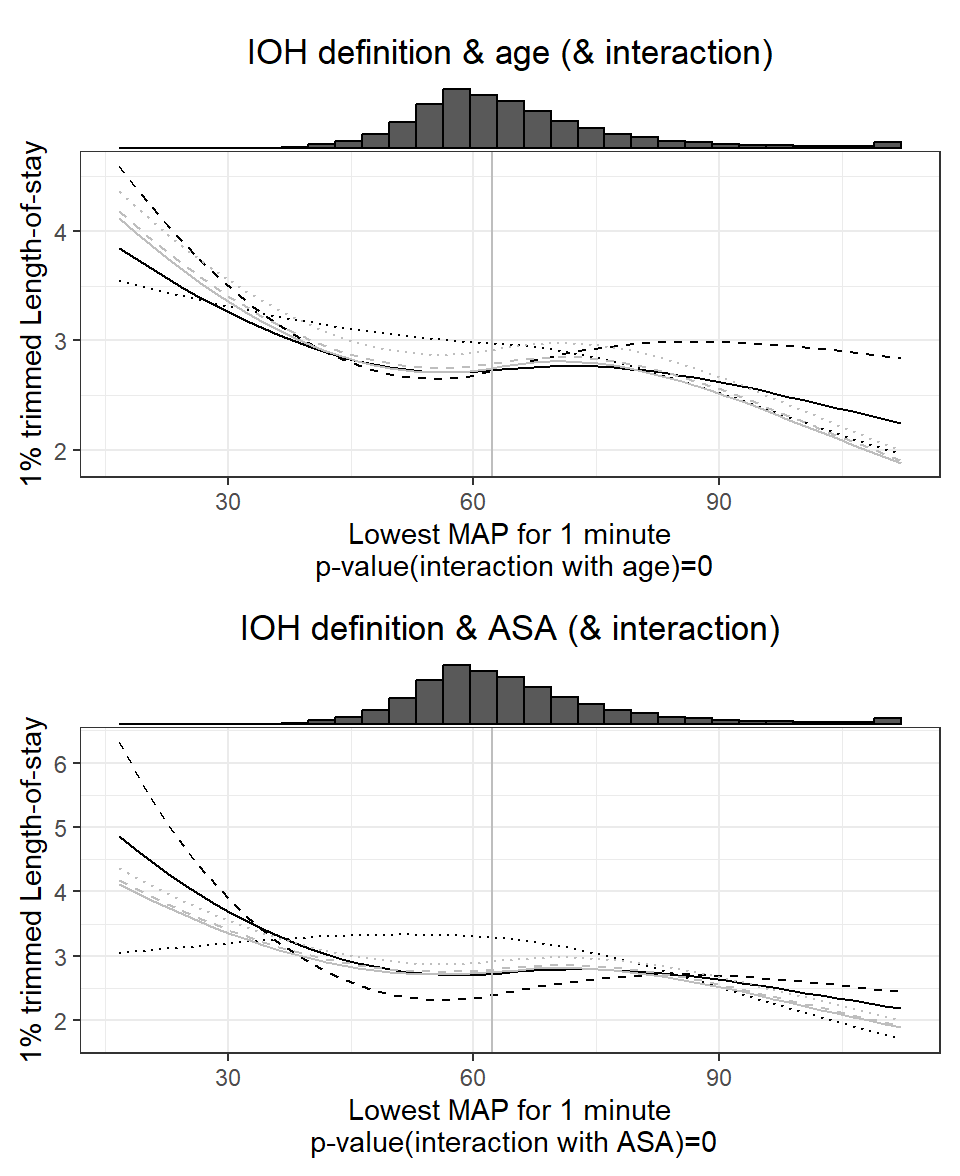


We test the clinically plausible interactions of the chosen operationalization with age (grey = model prediction without interaction, black = model prediction with interaction, solid = at median age/ASA2, dashed = at Q1 of age/ASA1, dotted = at Q3 of age/ASA3)
